# Variation in prostaglandin metabolism during growth of the diatom *Thalassiosira rotula*

**DOI:** 10.1038/s41598-020-61967-3

**Published:** 2020-03-25

**Authors:** Valeria Di Dato, Roberta Barbarinaldi, Alberto Amato, Federica Di Costanzo, Carolina Fontanarosa, Anna Perna, Angela Amoresano, Francesco Esposito, Adele Cutignano, Adrianna Ianora, Giovanna Romano

**Affiliations:** 1 Stazione Zoologica Anton Dohrn, Villa Comunale, 80121 Napoli, Italy; 2 Laboratoire de Physiologie Cellulaire Végétale, Université Grenoble Alpes, CEA, CNRS, INRA, IRIG-LPCV 38054 Grenoble Cedex 9, France; 3 Dipartimento di Scienze Chimiche, Università degli Studi di Napoli, Monte Sant’Angelo, 80126 Napoli, Italy; 4 Istituto di Chimica Biomolecolare-CNR, Via Campi Flegrei 34, 80078 Pozzuoli Napoli, Italy

**Keywords:** Fatty acids, RNA, Phylogenomics, Data mining, Gene expression profiling

## Abstract

Prostaglandins (PGs) are hormone-like mediators in many physiological and pathological processes that are present in all vertebrates, in some terrestrial and aquatic invertebrates, and have also been identified in some macroalgae. They have recently been reported also in marine microalgae but their role as chemical mediators is largely unknown. Here we studied the expression pattern of the PG biosynthetic pathway during different growth phases of the centric diatom *Thalassiosira rotula* and assessed the release of PGs in the surrounding environment for the first time. We show that enzymes responsible for PGs formation such as cyclooxygenase, prostaglandin E synthase 2-like and prostaglandin F synthase are mainly expressed at the end of the exponential phase and that PGs are released especially during the stationary and senescent phases, suggesting a possible signaling function for these compounds. Phylogenetic analysis of the limiting enzyme, COX, indicate the presence in diatoms of more than one enzyme related to the oxidative metabolism of fatty acids belonging to the peroxidase-cyclooxygenase superfamily. These findings suggest a more complex evolution and diversity of metabolic pathways leading to the synthesis of lipid mediators in diatoms.

## Introduction

Arachidonic acid (ARA), eicosapentaenoic acid (EPA), eicosatrienoic acid (ETrA) and docosahexaenoic acid (DHA) are polyunsaturated fatty acids that are physiologically important for animals at all taxonomic levels, including humans. EPA and DHA contribute to the healthy functioning of the cardiovascular system^1^ and are precursors of important classes of fatty acid-derivatives playing multiple signaling roles in inflammation and immune responses, platelet aggregation and tumor growth^2,3^. Among these, the inflammation process is one of the most important mechanisms adopted by organisms in response to various external stimuli^3^. Inflammation involves a complex interplay of signaling molecules whose final goal is to restore the healthy status of a cell or tissue. Consequences of sustained inflammation are indeed the development of serious diseases such as cancer and autoimmune disorders^4^.

Included in the eicosanoids are prostaglandins (PGs) synthesized principally from arachidonic acid (ARA) in animals, but also from EPA and ETrA, through the enzymatic route initiated by cyclooxygenase (COXs) enzymes^5^. PGs are molecules with a hormone-like behavior playing a prominent role in many physiological processes that have been principally studied in animals^3^. The expression of the COXs enzymes is mandatory for their synthesis. COXs exist in two isoforms that differ for their subcellular localization and for their expression timing. COX-1 is located in the endoplasmic reticulum and is constitutively expressed at constant levels in many tissues unless external cues, such as tumor promoting factor, cytokine and growth factor, induce an increase in its expression level. COX-2 is the inducible form, which is not detectable unless a trigger similar to those that stimulate COX-1 expression occurs. COX-2 is located in the nuclear envelope and appears to be a target for cancer therapy^6^.

PGs synthesis, in mammals, is initiated by phospholipases (PLAs), a family of enzymes that hydrolyze membrane phospholipids liberating the precursors, ARA, EPA, and ETrA. These are rapidly converted, through cyclization and inclusion of molecular oxygen, into the unstable metabolite PGG_2_ by the action of COXs enzymes that subsequently reduce it into PGH_2_. The PGH_2_ is then transformed into the ultimate prostaglandin E_2_, D_2_, F_2α_, prostacyclin or tromboxanes by the successive action of PGE, PGD, PGF, prostacyclin and tromboxanes A synthases^7^, respectively. Depending on which precursor is used, three series of PGs are produced, each having its own receptor series whose binding determine the type of message transduced^8^.

Plants synthesize molecules similar in structure to PGs, such as jasmonic acid, which regulates plant reproduction and fruit ripening processes^9^, responses to biotic and abiotic stresses, responses to external damage due to mechanical injury, herbivore and insect attack^10^. However, the presence of PGs has also been reported in some plant species, such as onions and poplar trees^11^.

In the marine environment, PGs have been isolated from the animal and plant kingdoms. In vertebrates such as sharks, carps and salmons they are involved in osmoregulation, regulation of branchial ion fluxes, ovulation and spawning^7^, while in invertebrates such as crustaceans, corals, and molluscs their role is still unknown. Among marine photoautotrophs, macroalgae and microalgae (e.g. *Euglena gracilis*) have shown PGs synthesis under stress conditions, however it is unclear what role they play in the mediation of stress response in these organisms^7^. Bacterial representatives have also been discovered with some cyanobacteria species synthesizing PGs^7^.

Despite the fact that only mammals have been deeply studied to understand PGs metabolism and functions, the discovery of PGs in other organisms should not be surprising. Indeed, evolutionary studies of the peroxidase-cyclooxygenase superfamily, to which COX enzymes belong, demonstrate its wide distribution in all the domains of life^12^, with seven main families that are well conserved. Family 5, containing the shortest bacterial sequence, named peroxicins, is supposed to be the most ancestral form, from which the first heme-peroxidases able to cope with reactive oxygen species might have evolved^12^. Its physiological role is however still completely unknown.

Family 4 represents a second evolutionary step towards bacterial and animal cyclooxygenases, with bacterial sequences that are still putative while many of the animal non-mammalian phyla, such as Cnidaria, Mollusca, Arthropoda and Chordata, have been cloned and studied even if the role of PGs also in these organisms is still unclear^12–14^. A peculiar branch of this family that diverged early from cyclooxygenases is the plant alpha dioxygenases (α-DOX) that partly lost the peroxidase activity maintaining instead the oxygenase activity responsible for the transformation of fatty acids to hydroperoxides.

In a previous study, we explored a diatom species for PGs metabolism and demonstrated the synthesis of all three series of PGs^15^. Diatoms are a class of stramenopiles characterized by a peculiar cell organization^16,17^, cell division mode^18^ and complex genomes^19^. They originated from a secondary endosymbiotic event^20^ which likely led to organisms displaying intricate metabolic pathways retrieved from the different entities that coexist in diatom genomes. Marine diatoms play a crucial role in the world carbon cycle being responsible for a non-negligible part of CO_2_ fixation on a yearly basis^21^. We demonstrated that two clones of *Skeletonema marinoi* differed for the expression level of the PG enzymatic pathway and for the amount of molecules produced^15^. In addition, *in-silico* analysis demonstrated that COX was present also in other diatom species and that diatom and animal COXs were remarkably similar^15^. In *Phaeodactylum tricornutum*, a model diatom species, compounds similar to PGs, called isoprostanoids, have been reported, despite the absence of COX sequence annotated in the genome. This finding suggests the existence of an alternative, non-enzymatic, origin of this class of compounds^22^.

Very recently, we found that the PGs pathway^23^ was up-regulated in the diatom *Thalassiosira rotula* under silica deprivation stress, in comparison with control conditions. *T. rotula* is a major diatom species blooming in many areas of the world’s oceans. This finding has fostered the current more thorough investigation on the expression and activity of the PG pathway in *T. rotula* during the different phases of its growth, measuring for the first time the release of PGs outside the cells into the surrounding growth medium. In addition, we conducted an evolutionary and *in silico* study of the *T. rotula* COX (TrotCOX) demonstrating that it actually belongs to the peroxidase-cyclooxygenase superfamily.

## Results

### *In-silico* structural reconstruction of TrotCOX

Since COX is the most relevant enzyme in the pathway, essential for the initiation of PGs synthesis, we used Phyre^2^ (Protein Homology/analogY Recognition Engine V 2.0)^24^ to predict the possible structure of TrotCOX protein and to confirm that it belongs to peroxidase-cyclooxygenase superfamily. The multiple alignments developed by the Phyre program retrieved as best hits 13 structures of proteins belonging to the peroxidase-cyclooxygenase family (Table 1, Supplementary Fig. 1). All of them shared 100% confidence and 94–99% coverage (Table 1). The first structure in the list shared a 19% identity and corresponded to a *Bos taurus* lactoperoxidase (BtLPO) already reported as representative of the peroxidase-cyclooxygenase superfamily^12^ (Table 1, Supplementary Fig. 2). Interestingly the second to the fifth structures corresponded to an *Ovis aries* prostaglandin G/H synthase (OaCOX1) all sharing a 28% identity, confirming the cyclooxygenase structure of the TrotCOX protein (Table 1, Supplementary Fig. 3). Structures from line 6 to 13, sharing lower identity (26% to 19%, Table 1), were however representative of cyclooxygenases, myeloperoxidase or oxidoreductase protein structures from *Homo sapiens*, *Mus musculus*, *Arabidopsis thaliana* and *Oryza sativa*, all of which are representative of the peroxidase-cyclooxygenase superfamily.

**Table 1:** Results of multi alignments of TrotCOX protein sequences with available protein structures.

Template	Template id/DOI/web link	Alignment Coverage	Confidence	% of identity
Buffalo lactoperoxidase	c2gjmA 10.2210/pdb2GJM/pdb	97	100	19
Sheep prostaglandin g/h synthase 1	c2oyup 10.2210/pdb2OYU/pdb	99	100	28
Sheep prostaglandin g/h synthase 1	c1pggB 10.2210/pdb1PGG/pdb	99	100	28
Sheep prostaglandin g/h synthase 1	c1ht8B 10.2210/pdb1HT8/pdb	99	100	28
Sheep prostaglandin g/h synthase 2	d1q4ga1 http://scop.berkeley.edu/search/?oldURL=1&key=%22d1q4ga1%22&key=d1q4ga1	99	100	28
*Dictyostelium discoideum* Peroxidase	c6ercA 10.2210/pdb6ERC/pdb	97	100	26
human promyeloperoxidase	c5mfaA 10.2210/pdb5MFA/pdb	97	100	20
Mouse Cycloxygenase 2	c1ddxA 10.2210/pdb1DDX/pdb	99	100	27
Mouse Cycloxygenase 2	c3pghD 10.2210/pdb3PGH/pdb	99	100	27
Mouse Cycloxygenase 2	d1cvua1 http://scop.berkeley.edu/search/?oldURL=1&key=%22d1cvua1%22&key=d1cvua1	99	100	27
Human Myeloperoxidase isoform C	c1d2vD 10.2210/pdb1D2V/pdb	94	100	20
Arabidopsis fatty acid alpha-dioxygenase (arabidopsis2 thaliana)	c4hhsA 10.2210/pdb4HHS/pdb	98	100	19
*Oryza sativa* fatty acid alpha-dioxygenase	c4kvjA 10.2210/pdb4KVJ/pdb	99	100	19

Refer to Supplementary Table 1 for a complete list.

**Figure 1:**
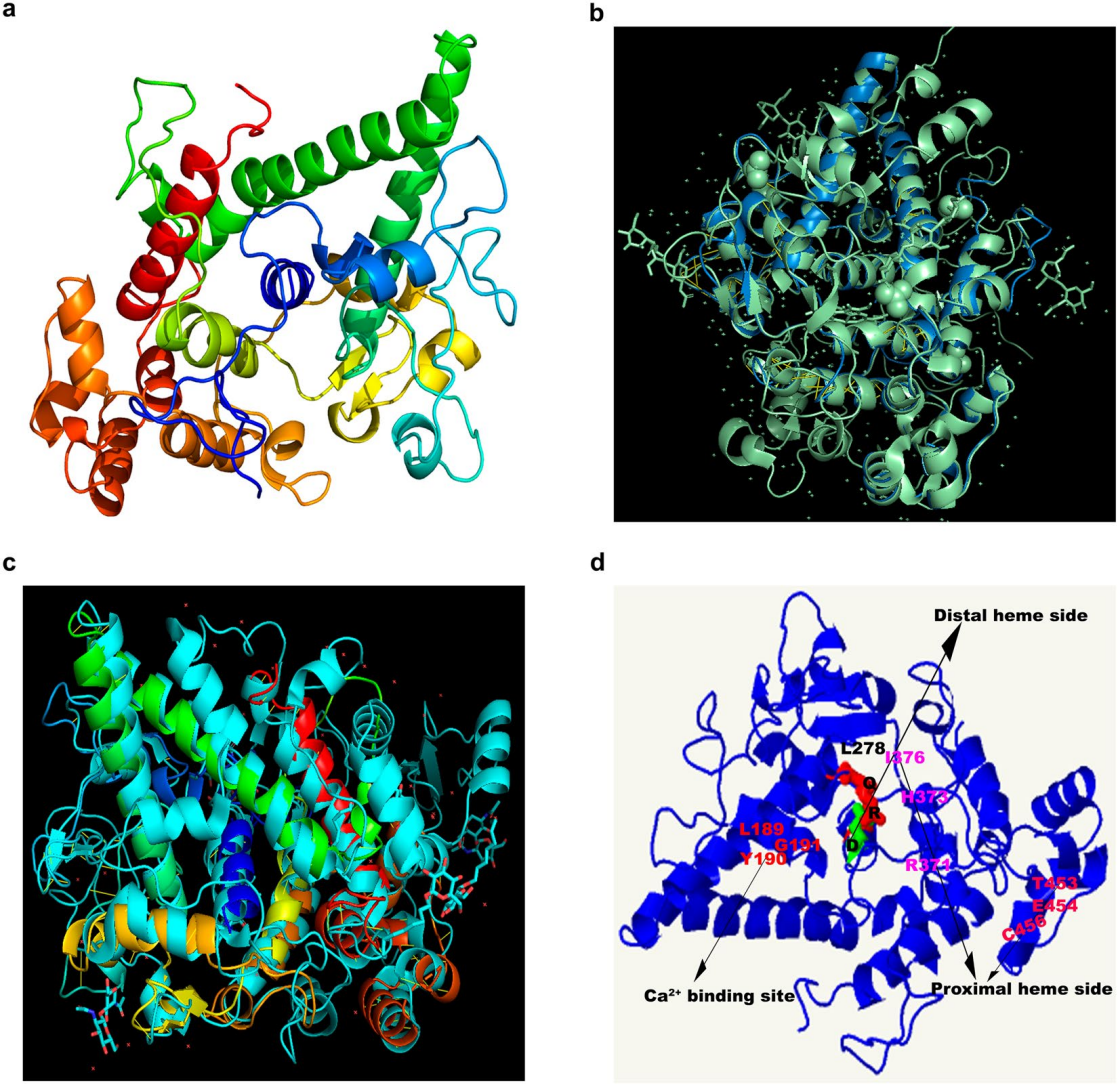
*In silico* reconstruction of the TrotCOX protein structure compared with the known reference proteins for the peroxidase-cyclooxygenase superfamily. (**a**) TrotCOX structure based on the 2.75 Å resolved x-ray diffraction of BtLPO. (**b**) Comparison of the *in silico* structure of the TrotCOX protein and the 2.75 Å resolved x-ray diffraction of BtLPO showing the perfect overlap of the alpha-helices embedding the heme sides. In blue the TrotCOX, in light green the BtLPO. (**c**) Comparison of the *in silico* structure of the TrotCOX protein and the 2.75 Å resolved x-ray diffraction of OaCOX1 showing the perfect overlap of the alpha-helices embedding the heme sides. Rainbow colored helices correspond to TrotCOX, the light-blue colored helices correspond to OaCOX1. (**d**) Illustration of the conserved catalytic sites described in the text. Q, R, D and L278 black letters refer to the conserved amino acids of the two distal heme sides with L278 being the mutated original E. Pink and light red letters indicate the conserved amino acids in the two proximal heme sides. The red letters indicate the amino acids forming the calcium binding site.

Figure 1b,c show the results of the comparison among TrotCOX (Fig. 1a) predicted structure and known models of the peroxidase-cyclooxygenase family, alias the BtLPO (3BXI.pdb, 10.2210/pdb3bxi/pdb)^12,25^ and OaCOX1 structures. The main alpha helices that characterize the cyclooxygenases and accommodate the heme prosthetic group are present and almost perfectly overlapped with the references BtLPO and OaCOX1.

**Figure 2:**
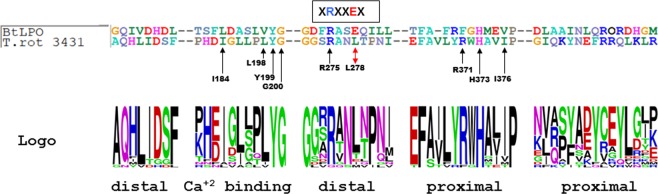
TrotCOX vs BtLPO amino acid sequence alignment at the distal and proximal heme sides and at the calcium binding sites^32^. The sequence Logo of the same portion of the alignment for the diatom specific clade is shown below. The sequence Logo is a resumed representation of an alignment among two or more sequences. The height of each letter is proportional to its frequency and the most common one is placed on the top^55^. Important amino acidic residues reported in the text are indicated on the *T. rotula* sequence. BtLPO = *Bos taurus* LPO; T. rot = *Thalassiosira rotula*.

The *in silico* predicted model constructed on the BtLPO showed conserved domain sites in the diatom sequences (Fig. 1d and Fig. 2): catalytic arginine was found (R275) in the distal heme side, embedded in the characteristic motif XRXXEX, while glutamate that is usually conserved, was not in TrotCOX but was mutated to leucine (L278). The latter seems to be a characteristic of diatoms as none of the diatom sequences (Supplementary Fig. 4) here analyzed showed a glutamate in that position. The proximal heme sides are relatively conserved in TrotCOX with the highly conserved arginine (R371), histidine (H373), isoleucine (I376) and glutamic acid (E454) unvaried in TrotCOX and in most of the other sequences. Surprisingly, the conserved asparagine and arginine that are normally bonded via a hydrogen bond were substituted with a threonine (T453) and a cysteine (C456), respectively. The calcium binding site in TrotCOX shows the relatively conserved Isoleucine (I184) and LYG motif (L198, Y199, G200) (Figs. 1d and Fig. 2). The sequence representation by Logo (Fig. 2) confirmed this residue conservation also among all the diatom sequences considered for the phylogenetic analysis.

**Figure 3:**
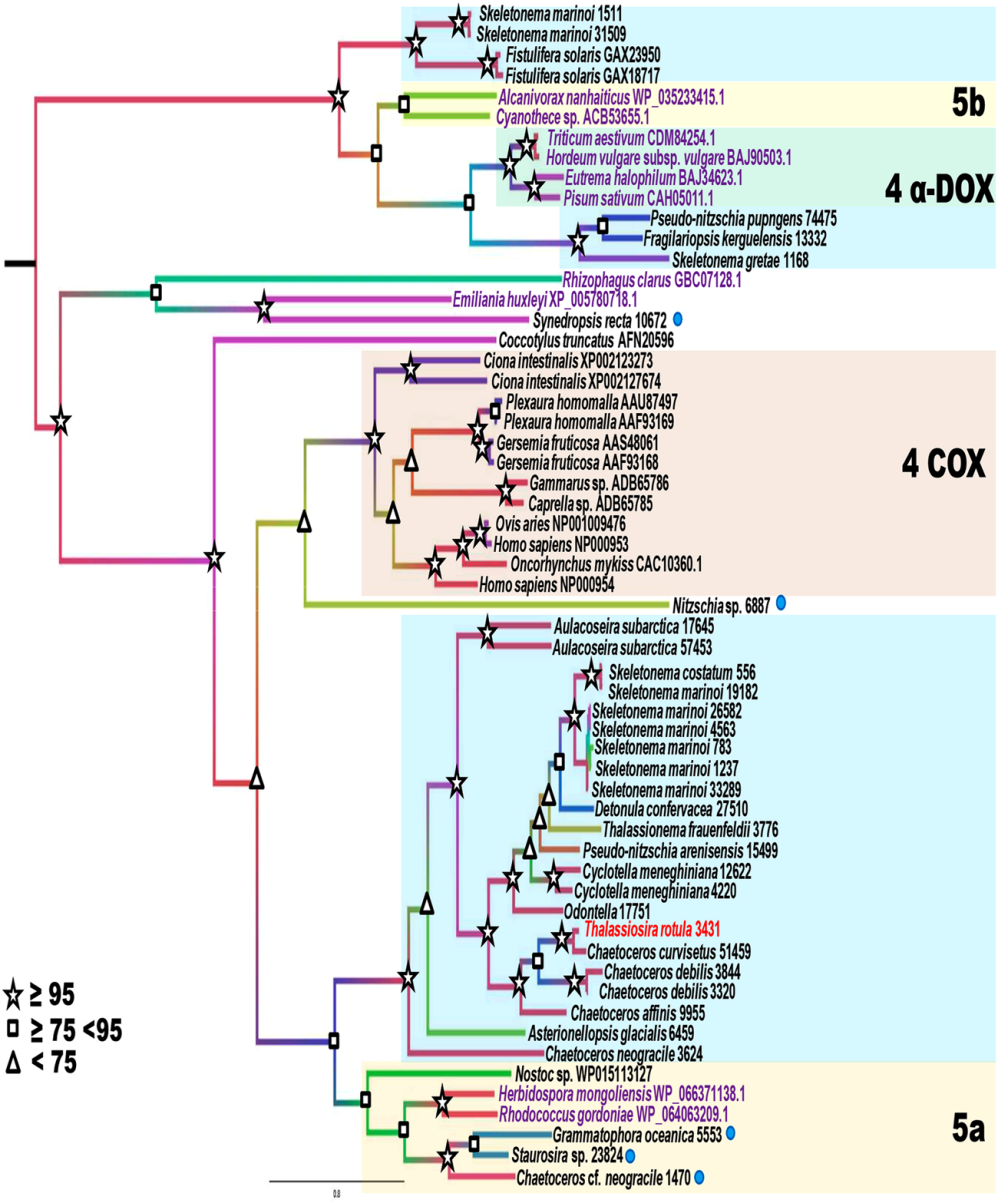
Phylogenetic analysis of the *Thalassiosira rotula* COX. Diatom sequences are scattered in the ML phylogenetic tree, either in diatom specific clades, or clustered together with sequences from other taxa. Legend: TrotCOX is indicated in red; cyan dot: diatom sequences not clustering the diatoms-specific clade; cyan-shaded clade: diatom specific clade; 5a: 5a-family in the diatom/bacterial clade; 4 COX: animal COX clade (family 4, cyclooxygenases); 4 α-DOX: plant α-DOX proteins (family 4, cyclooxygenases); 5b: 5b-family cyanobacteria. The nomenclature used for the family classification follows Zámocký *et al*.^12^.

Using a previous alignment^15^, populated with other sequences retrieved from NCBI via a pBLAST, we performed a refined phylogenetic analysis comparing the COX sequence from *T. rotula* with that of other organisms (Supplementary Tables 1 and 2). Most of the diatom sequences clustered in a large well-supported clade (Maximum Likelihood (ML) bootstrap value 100) closely related to the animal COX clade (family 4, cyclooxygenases), although the phylogenetic relationship was not well resolved (ML 35) (Fig. 3).

This large diatom clade was divided into a main well-supported clade including the vast majority of diatom sequences, among which the TrotCOX sequence (Fig. 3), and in an end-clade (ML 94) grouping both prokaryotic (*Nostoc* sp., a cyanobacterium, the actinomycetales *Herbidospora mongoliensis* and *Rhodococcus gordoniae*) and diatom (*Grammatophora oceanica*, *Staurosira* sp., and *Chaetoceros* cf. *neogracile*) sequences. This diatom/bacterial clade can be hypothesized as being the 5a family within the peroxidase-cyclooxygenase super family^12^. Noteworthy, the pennate biraphid diatom sequence from *Nitzschia* sp. clusters basally to the animal COX clade (ML 56). In addition to that large clade, a second very well resolved (ML 100) super clade was composed of two clades. One of these forks includes only diatom sequences (*Fistulifera solaris* and *S. marinoi* 31509 and 1511); whereas the other bifurcates in two sister clades. One of these sister clades groups only two prokaryotic sequences, and the other bifurcates in two branches. The first branch groups plant α-DOX proteins (family 4 cyclooxygenase^12^) and the other groups diatoms (*Skeletonema gretae*, *Pseudo-nitzschia pungens* and *Fragilariopsis kerguelensis*) (Fig. 3).

### Gene expression analysis

**Figure 4:**
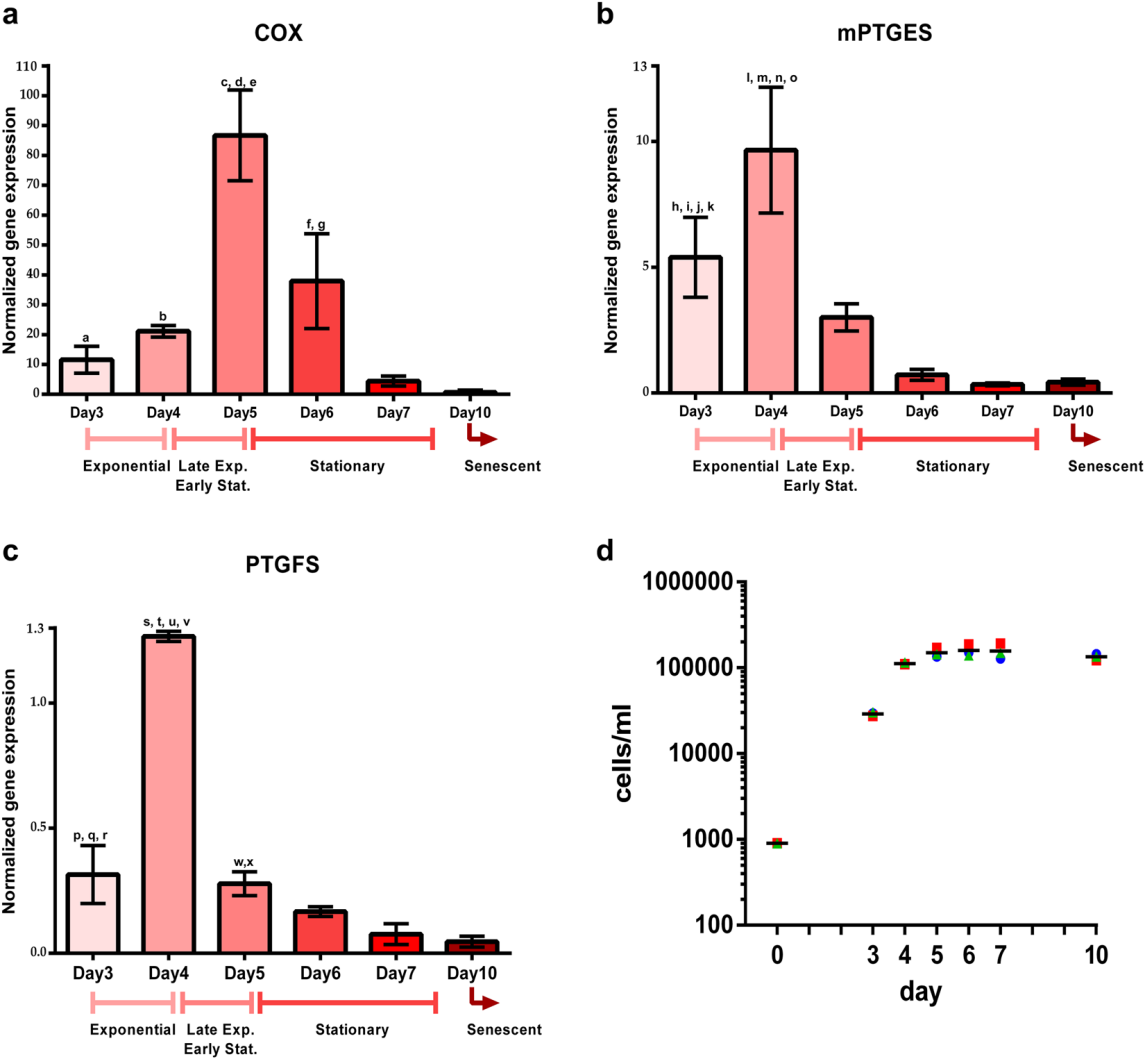
Gene expression analysis of the three genes identified in the *Thalassiosira rotula* prostaglandin pathway. Expression levels of COX (**a**), mPTGES (**b**) and PTGFS (**c**) genes during the growth of *T. rotula* from day 3 to day 10. Results are based on TBP housekeeping gene normalized value. Normalization based on Actin as housekeeping gene gave the same results. Panels a, b and c report the corresponding growth phase (Abbreviations: Exp.: Exponential phase; Stat.: Stationary phase). Y axis reports the Mean Normalized Expression (MNE) of three biological and technical replicates using TBP as reference gene. Letters on bars refer to significant daily comparisons. Refer to Table 2 for the levels of significance and the corresponding comparisons. (**d**) Growth curves of the 3 independent *T. rotula* cultures utilized to analyze gene expression. Horizontal lines over the symbols indicate the mean value of the three values for each time point. Y axis is in logarithmic scale.

Figure 4 illustrates differential expression analysis by qPCR of the three genes involved in the PG pathway during *T. rotula* growth (Fig. 4d). One-way ANOVA analysis shows, for each gene, a statistically significant difference (0.0001 < p-value < 0.0002) among the six time points considered along the 10-day *T. rotula* growth curve (Fig. 4, Table 2).

**Table 2:** One-way ANOVA analysis over the time points along the 10 day *T. rotula* growth curve showing day by day comparisons for each gene with the corresponding significance.

	Tukey’s multiple comparisons test	Adjusted p-value	Significant	Reference symbol in Fig. 4	Adjusted p-value	Significant	Reference symbol in Fig. 4	Adjusted p-value	Significant	Reference symbol in Fig. 4
gene		COX			mPTGES			PTGFS		
ANOVA P value		0.0002			0.0002			<0.0001		
	Day3 vs Day4	0,8823	ns		0,0412	*	h	<0,0001	****	p
	Day3 vs Day5	0,0005	***	a	0,4826	ns		0,9745	ns	
	Day3 vs Day6	0,1282	ns		0,0247	*	i	0,1358	ns	
	Day3 vs Day7	0,9720	ns		0,0298	*	j	0,0079	**	q
	Day3 vs Day10	0,8710	ns		0,0328	*	k	0,0038	**	r
	Day4 vs Day5	0,0007	***	b	0,0056	**	l	<0,0001	****	s
	Day4 vs Day6	0,3705	ns		0,0003	***	m	<0,0001	****	t
	Day4 vs Day7	0,4802	ns		0,0005	***	n	<0,0001	****	u
	Day4 vs Day10	0,3069	ns		0,0005	***	o	<0,0001	****	v
	Day5 vs Day6	0,0053	**	c	0,5259	ns		0,3359	ns	
	Day5 vs Day7	0,0003	***	d	0,4658	ns		0,0207	*	w
	Day5 vs Day10	0,0002	***	e	0,4979	ns		0,0094	**	x
	Day6 vs Day7	0,0442	*	f	0,9996	ns		0,4457	ns	
	Day6 vs Day10	0,0263	*	g	0,9999	ns		0,2070	ns	
	Day7 vs Day10	0,9988	ns		>0,9999	ns		0,9740	ns	

Abbreviations: ns: not significant; *adjusted p-value < 0.05; **adjusted p-value < 0.001; ***0.0001 < adjusted p-value < 0.0005; ****p-value < 0.0001.

Each gene showed a statistically significant expression peak around the late exponential/early stationary phase, i.e. day 5 for COX and at day 4 for mPTGES and PTGFS (Fig. 4a and Table 2). In addition, COX-day 6 expression persisted with significant difference versus COX-day 7 and COX-day 10 expressions. mPTGES-day 3 expression was statistically significantly higher than mPTGES-days 6, 7 and 10 expressions (Fig. 4b and Table 2) but had levels lower than mPTGES-day 4 expression. Similarly, PTGFS-day 3 expression was statistically significantly lower than day 4 but higher than PTGFS-days 7 and 10 expressions. Analogously, PTGFS-day 5 expression was still statistically significantly higher than PTGFS-day 7 and 10 expressions (Fig. 4c and Table 2).

The expression peaks were only transient because a quick decrease in the expression levels occurred soon afterwards, reaching very low levels at the end of the stationary (day 7) and senescent (day 10) phases. Moreover, the initial basal expression levels of the three enzymes were completely different. COX expression was much higher compared to mPTGES and PTGFS, and mPTGES was more expressed compared to PTGFS (Fig. 4, Day3). However, from day 7 to 10, the expression of all three genes decreased, reaching similar levels. The reference genes^23^ utilized were stable, indicating that the down regulation of the analyzed genes was not due to senescence but rather to a specific pathway response.

### LC-MS/MS analysis

**Figure 5:**
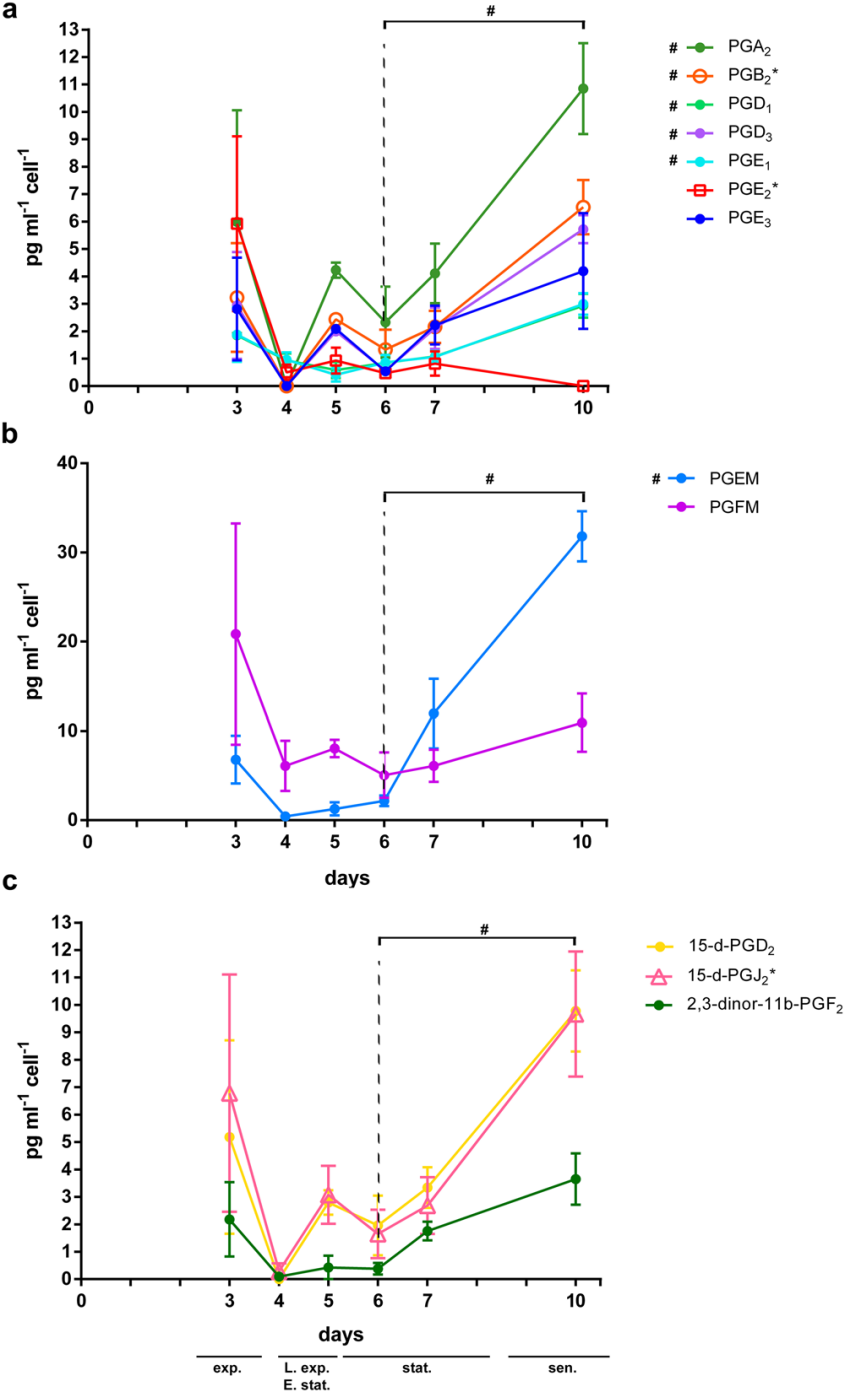
LC-MS/MS identification and quantification of prostaglandins in the culture medium during *Thalassiosira rotula* growth. Concentration trends of the prostaglandins in pg mL^−1^ cell^−1^ during the 10 days were identified and measured by either comparison with pure standards (asterisks) or by comparison with fragmentation data reported in the literature. # indicates PGs for which Day 6 vs Day10 values are significantly different by one-way ANOVA analysis considering day 6 as reference time point. Dashed lines refer to day 6.

In addition to gene expression levels, we also studied the pathway activity by identifying the PGs released outside the cells by *T. rotula* and measured their concentration in the culture medium (Fig. 5a–c). By LC-MS/MS in comparison with pure standards (Supplementary Figs. 5–8), we detected three PGs in the culture medium, namely PGE_2_, PGB_2_ and 15-d-PGJ_2_. In addition, in the same medium, we also identified PGA_2_, PGD_1_, PGD_3_, PGE_1_, PGE_2_, PGE_3_, PGEM, PGFM, 15-d-PGD_2_ and 2,3-dinor-11b-PGF_2_ that we classified as “putative” since they have been identified through comparison of experimental mass fragmentation data obtained in this study with those reported in the literature (Supplementary Fig. 9, Supplementary Table 3)^26,27^.

Overall, a high variability among replicates was evident as indicated by the large error bars (Fig. 5a–c). In addition, we observed an up-and-down trend of each measured PG concentration from days 3 to 5. Conversely, measurements from day 6, corresponding to the full stationary phase, to day 10, showed a linear trend toward increasing concentration values, except for the PGE_2_, PGE_3_, 15-d-PGJ_2_, and PGFM (Fig. 5a–c). In particular, PGE_2_ had a constant low level reaching zero on day 10 (Fig. 5a), while PGEM showed an exponential trend starting from day 4, i.e. late exponential phase of growth, reaching the highest value among all the other PGs identified (Fig. 5b).

One-way ANOVA analysis (Table 3) shows a statistically significant difference over the considered time points for PGA_2_, PGB_2_, PGD_1_, PGD_3_, PGE_1_, PGEM, 15-d-PGD_2_ and 2,3-dinor-11b-PGF_2_. Comparison of day 4 versus day 10 values were statistically significant for the majority of the compounds, due to concentrations dropping to zero on day 4 (Fig. 5). PGEM concentrations, varied significantly from day 4 to 10, even if some differences were detected, i.e. Day5 vs Day6, Day5 vs Day7 and Day6 vs Day7 were not significant because of the large error bars (Fig. 5c, Table 3).

**Table 3:** One-way ANOVA analysis over the time points considered during the 10 day *Thalassiosira rotula* growth curve.

PG	Tukey’s multiple comparisons test	Adjusted p-value/Significance											
		PGA_2_	PGB_2_	PGD_1_	PGD_3_	PGE_1_	PGE_2_	PGE_3_	PGEM	PGFM	15-d-PGJ_2_	15-d-PGD_2_	2,3-dinor-11b-PGF_2_
ANOVA P value		0.0306*	0.001**	0.042*	0.008**	0.028*	0.068ns	0.221ns	<0.0001****	0.385ns	0.0701ns	0.0207*	0.0289*
	Day3 vs Day4	0,3042 ns	0,255 ns	0,759 ns	0,241 ns	0,754 ns	0,110 ns	0,5728 ns	0,4080 ns	0,4514 ns	0,3189 ns	0,3011 ns	0,3651 ns
	Day3 vs Day5	0,9845 ns	0,991 ns	0,463 ns	0,969 ns	0,317 ns	0,158 ns	0,9976 ns	0,5487 ns	0,5900 ns	0,8117 ns	0,9034 ns	0,5375 ns
	Day3 vs Day6	0,7557 ns	0,743 ns	0,683 ns	0,439 ns	0,670 ns	0,108 ns	0,7541 ns	0,7086 ns	0,3843 ns	0,5486 ns	0,7405 ns	0,5104 ns
	Day3 vs Day7	0,9794 ns	0,968 ns	0,856 ns	0,982 ns	0,832 ns	0,144 ns	0,9991 ns	0,6146 ns	0,4521 ns	0,7442 ns	0,9648 ns	0,9979 ns
	Day3 vs Day10	0,5089 ns	0,238 ns	0,624 ns	0,289 ns	0,588 ns	0,071 ns	0,9574 ns	<0,0001****	0,7949 ns	0,9210 ns	0,4168 ns	0,6952 ns
	Day4 vs Day5	0,6404 ns	0,525 ns	0,994 ns	0,610 ns	0,9581 ns	0,999 ns	0,8104 ns	0,9998 ns	0,9998 ns	0,9309 ns	0,8326 ns	0,9993 ns
	Day4 vs Day6	0,9504 ns	0,922 ns	0,999 ns	0,997 ns	0,9999 ns	0,999 ns	0,9994 ns	0,9931 ns	>0,9999 ns	0,9970 ns	0,9550 ns	0,9997 ns
	Day4 vs Day7	0,6655 ns	0,637 ns	0,999 ns	0,557 ns	0,9999 ns	0,999 ns	0,7685 ns	0,0349*	>0,9999 ns	0,9624 ns	0,7139 ns	0,5886 ns
	Day4 vs Day10	0,0174*	0,005**	0,103 ns	0,006**	0,0916 ns	0,999 ns	0,2003 ns	<0,0001****	0,9873 ns	0,0732 ns	0,0128*	0,0381*
	Day5 vs Day6	0,9786 ns	0,963 ns	0,998 ns	0,851 ns	0,9823 ns	0,999 ns	0,9345 ns	0,9997 ns	0,9986 ns	0,9961 ns	0,9990 ns	>0,9999 ns
	Day5 vs Day7	0,9999 Ns	0,999 ns	0,974 ns	0,999 ns	0,9169 ns	0,999 ns	0,9999 ns	0,0542 ns	0,9998 ns	>0,9999 ns	0,9999 ns	0,7733 ns
	Day5 vs Day10	0,219 ns	0,098 ns	0,043*	0,090 ns	0,0233*	0,995 ns	0,8025 ns	<0,0001****	0,9988 ns	0,3050 ns	0,0944 ns	0,0663 ns
	Day6 vs Day7	0,9839 ns	0,989	0,999 ns	0,808 ns	0,9995 ns	0,999 ns	0,9091 ns	0,0861 ns	>0,9999 ns	0,9992 ns	0,9899 ns	0,7478 ns
	Day6 vs Day10	0,0731 ns	0,026*	0,082 ns	0,013*	0,0714 ns	0,999 ns	0,3164 ns	<0,0001****	0,9706 ns	0,1516 ns	0,0523 ns	0,0612 ns
	Day7 vs Day10	0,2056 ns	0,071 ns	0,142 ns	0,105 ns	0,118 ns	0,997 ns	0,8417 ns	0,0005***	0,9874 ns	0,2539 ns	0,1367 ns	0,4585 ns

One-way ANOVA, calculated considering day 6 as reference point, shows the same significant differences over time points confirming that differences in concentrations at day 6 vs day 10 are statistically significant for all the PGs, except for PGE_2_, PGE_3_, 15-d-PGJ_2_, and PGFM (Table 4).

**Table 4:** One-way ANOVA analysis over the time points day 6 to day 10 of the ten day *Thalassiosira rotula* growth curve, considering Day 6 as reference time point.

PG	Tukey’s multiple comparisons test	Adjusted p-value/Significance											
		PGA_2_	PGB_2_	PGD_1_	PGD_3_	PGE_1_	PGE_2_	PGE_3_	PGEM	PGFM	15-d-PGJ_2_	15-d-PGD_2_	2,3-dinor-11b-PGF_2_
ANOVA P value		0.0306*	0.001**	0.042*	0.008**	0.028*	0.068ns	0.221ns	<0.0001****	0.385ns	0.0701ns	0.0207*	0.0289*
	Day6 vs Day3	0,5521	0,537	0,472	0,257	0,458	**0,051**	0,550	0,498	0,218	0,345	0,534	0,313
	Day6 vs Day4	0,8574	0,8	0,999	0,987	0,99	>0,999	0,997	0,973	0,999	0,987	0,868	0,998
	Day6 vs Day5	0,9278	0,9	0,993	0,679	0,938	0,999	0,823	0,998	0,994	0,984	0,995	>0,999
	Day6 vs Day7	0,9430	0,1	0,996	0,618	0,997	0,999	0,774	**0,04**	0,999	0,996	0,962	0,543
	Day6 vs Day10	**0,0334**	**0,011**	**0,038**	**0,006**	**0,032**	0,999	0,172	**<0,0001**	0,906	0,074	**0,023**	**0,028**

## Discussion

Diatoms are a very important group of microalgae populating all aquatic niches and able to fix about 20% of global carbon production^28^. Their elevated adaptability is due to a rich set of metabolic pathways coded by their genome^29^. Their crucial ecological role^30,31^ is coupled to an emerging biotechnological interest due to their ability to produce high added value molecules. One of the metabolisms recently identified in these microalgae is the pathway for the enzymatically-mediated synthesis of prostaglandins (PGs), principally studied in mammals but less in plants or microorganisms^15,23^.

The key step in PG biosynthesis is catalyzed by COX enzymes belonging to the heme-peroxidase protein superfamily. Evolutionary studies of this superfamily demonstrated an independent evolution of four superfamilies, including peroxidase–cyclooxygenase^12^, each possessing a peculiar folding of the heme peroxidase domain. In particular, the peroxidase-cyclooxygenase superfamily has a wide distribution in all living kingdoms^12^.

Phylogenetic analysis of the *Thalassiosira rotula* COX presented here widens the analysis reported previously in Di Dato *et al*.^15^ with the addition of a few sequences that have better resolved some of the clades identified in that study. In particular, the sequences from *Grammatophora oceanica* 5553, *Staurosira* 23824, *Chaetoceros cf. neogracile* 1470 clustered with *Nostoc* WP015113127 thereby questioning the validity of the analysis. It was not clear enough whether the clade grouping the three diatom and *Nostoc* sequences could be identified as a diatom specific clade derived from cyanobacteria. In the present analysis, new bacterial sequences were added and clustered together with the above-mentioned diatom and *Nostoc* sequences showing that this clade is indeed a bacterial-like clade. Moreover, our phylogenetic analysis highlights the existence of more than one peroxidase-cyclooxygenase enzyme related to the oxidative metabolism of fatty acids in diatoms. The four sub-families in the peroxidase-cyclooxygenase superfamily (family 4: cyclooxygenase and α-DOX; 5a and 5b, bacterial peroxidase-cyclooxygenase families) did not evolved from one single protein, but rather appeared independently during evolution^32^. COX, were formerly known as ‘animal heme-dependent peroxidases’. However, as demonstrated here and in our previous work^15,23^, this enzyme is present and active in diatoms as well, thus the denomination ‘animal heme-dependent peroxidases’ would deserve a revision in order to avoid confusion.

α-DOX belongs to the same peroxidase–cyclooxygenase superfamily as COX and some diatoms (e.g. *S. marinoi*) possess this protein along with COX. This questions the origin and evolution of this class of enzymes in diatoms and shows that this group of microalgae is capable of manipulating fatty acids in different ways: diatom α-DOX enzymes may act on medium chain saturated fatty acids, such as 16:0^33^, while COX on long chain-PUFAs (e.g. AA and EPA)^34^. Also, the close proximity of bacterial and diatom sequences and the topology of the tree we present, questions whether diatoms acquired the genes coding for COX via lateral gene transfer^35^, which occurred during diatom evolution^36–38^ or whether these genes were acquired during endosymbiotic events, at least for species like *S. marinoi* possessing both α-DOX-like and COX-like enzymes. In the case of *T. rotula*, α-DOX-like is absent, while the pennate model diatom *Phaeodactylum tricornutum* completely lacks the enzymatic pathway leading to prostaglandins though it can produce isoprostanoids non-enzymatically^22^. Indeed, the ability to synthesize eicosanoids, including lipoxygenase-derived oxylipins and aldehydes, is not robustly conserved in diatoms^39,40^ where genes involved in these metabolisms might have been lost during evolution.

In our *in silico* study of TrotCOX protein sequence and structure we found an overall homology and the conservation of motifs characterizing the heme-peroxidase protein superfamily, corroborating also the assignment of this sequence to the peroxidase-cyclooxygenase superfamily^12^. The catalytic sites, the motifs and their order along the protein sequence, are very conserved compared to the representative sequences of the peroxidase-cyclooxygenase superfamily, even if with some differences. The glutamate embedded in the characteristic motif XRXXEX, usually conserved, in TrotCOX is mutated into a leucine. This mutation seems to be characteristic of diatoms that not only show mutations in TrotCOX but also in other diatom sequences considered for the phylogenetic analysis. Considering the different cellular environments, the leucine residue may still play in diatoms the same role as glutamic acid in other organisms although the glutamic acid is a charged amino acid while leucine is a hydrophobic uncharged amino acid. In addition, in the proximal heme sides, the conserved asparagine and arginine normally linked via a hydrogen bond, playing crucial roles in alpha helix structures^41^, are substituted with a threonine and a cysteine, respectively. This change should not compromise the alpha helix stability present in this region.

Our experimental data demonstrate that TrotCOX is active and works like the animal COX, being able to synthesize the same PGs produced by animals. The presence of PGE_3_, a specific derivative of EPA, is in line with the abundance of this PUFA precursor, which represents a hallmark of diatoms^42^.

Interestingly, the general increase of PGs released outside the cells started from day 5 onwards, i.e., when cells were entering the stationary phase that coincides with the maximum expression of COX. This stage is generally associated with an increase in the naturally associated bacterial population. During the transition between the stationary and senescent phases, nutrients decrease while dying algal cells generate organic matter that can feed associated bacteria. Bacteria are able to assimilate phosphate better than algae, especially at low concentrations^43^, and compete with algae for inorganic nutrients^44^. This increased pressure may stimulate PGs synthesis in diatoms as already shown in animals, where COX expression is stimulated in the presence of bacteria^45^ and in humans where PGE_2_ have been correlated to viral load and infection severity in influenza^46–49^. In line with this hypothesis, similar compounds, such as hydroxylated eicosapentaenoic acid 15-HEPE, were shown to be up-regulated and released from cells when the diatom *S. marinoi* was exposed to pathogenic bacteria^50^.

PGE_2_ is the most abundant prostanoid in the human body, but it is also very unstable since it is rapidly converted into PGE-M^46–49^ that is considered to mirror the systemic levels of PGE_2_ formation. In our study, PGE_2_ was present at low levels in the medium during the exponential growth phase and was almost absent in the late stationary phase (from day 7) while PGE-M levels increased exponentially up to the senescent phase (Fig. 4). The peak in the expression of the related enzyme, PTGES, at the onset of the stationary phase and the sustained release of PGs afterwards suggest a possible role of prostaglandins in communication and cell signaling. Their release outside the cells, in addition to their sustained presence in a saline environment, is quite striking and has never been observed before in diatoms. PGs are known to exert a wide range of effects in different organisms, including the induction of inflammatory processes, injury and pain in humans where they have been best studied. The fact that they have been identified in organisms ranging from unicellular diatoms, corals and jellyfish to arthropods, mollusks and mammals denotes that they regulate important physiological processes that have been conserved through evolution.

In conclusion, this study confirms that diatoms possess a molecular toolbox generally believed to be unique to higher organisms such as mammals. The production and release of PGs by some diatoms and the variation in the expression levels of PG biosynthetic pathway during different growth stages, strongly suggest a relevant and possible signaling role for these molecules within the plankton community that needs to be further investigated. This characteristic may have contributed to render diatoms one of the most successful groups of organisms in the world’s oceans.

## Methods

### Strain and cell cultures

*Thalassiosira rotula*, strain FE80C, was isolated in 2011 in the Gulf of Naples (40°48.5’N, 14°15’E), Mediterranean Sea. Clonal cultures were established by isolating single cells or short chains from phytoplankton net samples collected from the surface layer of the water column. Cultures were grown in sterile filtered oligotrophic seawater at 36 psu amended with f/2^51^ nutrients and maintained at a temperature of 20 °C, at 12:12 h light:dark cycle, and with a photon flux of 100 μmol photons m-^2^s^−1^.

10-liter cultures of *T. rotula*, in triplicate, were used to follow their growth from day 3 to day 10. Every day, 250 mL of each culture was harvested by filtration onto 1.2 μm pore size filters (RAWP04700 Millipore) and immediately frozen in liquid nitrogen. 100–200 mL of culture media recovered from the cell filtration was collected and stored at −80 °C until sample processing. Initial cell concentrations were about 5000 cells/mL upon inoculation. Culture growth was monitored daily from samples fixed with one drop of Lugol (final concentration of about 2%) and counted in a Bürker counting chamber under an Axioskop 2 microscope (20×) (Carl Zeiss GmbH, Jena, Germany).

### RNA extraction and reverse transcription

To proceed to total RNA extraction, filters were covered with 1.5 mL TRIsure (BIO-38033, Bioline) to which glass beads (G1277, Sigma-Aldrich) were added. Cells were disrupted on a thermo-shaker (Eppendorf) at 60 °C for 10 minutes at 1200 r.p.m. Filter and glass beads were discarded, and the extraction was continued according to the TRIsure instructions. DNase treatment was carried out using DNase I recombinant, RNase-free (Roche, Basel, Switzerland) according to manufacturer’s protocol to eliminate potential genomic DNA contamination. The efficiency of the DNase digestion was checked by testing DNA primer capability to amplify an amplicon with traditional PCR. PCR reactions were carried out in 25 μL volume with 2,5 μL of 10× PCR reaction buffer (Roche, Basel, Switzerland), 2,5 μL of 2 mM dNTP, 0.3 μL of 5 U/μL Taq (Roche, Basel, Switzerland), 1 μLof 20 pmol/μL for each oligo, 1 μL of RNA templates and nuclease-free water up to 25 μL. The PCR program consisted in a denaturation step at 95 °C for 5 min, 35 cycles at 95 °C for 30 s, 53 °C for 30 seconds and 72 °C for 45 s, and a final extension step at 72 °C for 7 min. Amplified PCR products were analyzed by agarose gel electrophoresis.

Total RNAs were purified and concentrated using RNeasy MinElute Cleanup Kit (Qiagen, Venlo, Netherlands) and eluted in 30 μL of RNase-free water. Concentrations of the resulting RNA samples were assessed by absorbance at 260 nm (ND-1000 Spectrophotometer; NanoDrop Technologies, Wilmington, DE, USA). The integrity of total RNA was checked by agarose gel electrophoresis. One microgram of each RNA sample was retro-transcribed in complementary DNA (cDNA) following the manufacturer’s instructions (5X All-In_One RT Master Mix, abm, Applied Biological Materials Inc., Richmond, Canada) using the T100 Thermal cycler (Bio-Rad Laboratories, Hercules, CA, USA). Retro-transcribed samples were again checked for DNA contamination by traditional PCR, as above, using intron-spanning primer pairs.

### Phylogenetic analyses

For cyclooxygenase phylogeny, the alignment from Di Dato *et al*.^15^ was retrieved and all the sequences were blasted against the NCBI database. The best hits from each pBLAST were added to the analysis. Sequences were visualized and aligned in BioEdit Sequence Alignment Editor^52^ using the ClustalW alignment algorithm implemented in BioEdit. The alignment was performed using MUSCLE online software and compared to the ClustalW output. The two alignments were identical. For maximum likelihood (ML) phylogenetic analysis both MEGAX^53^ and RAxML were used. Model test implemented in MEGAX identified the LG + G + I as the best substitution model on the basis of the Bayesian Information Criterion (BIC). 1000 bootstrap replicates were performed. Two different analyses were run in RAxML, one with the boot stopping option and one with 100 bootstrap replicates. All the analyses involved 58 amino acid sequences and 612 positions (including gaps). The two RAxML and the MEGA X trees were identical in topology and support. Phylogenetic trees were visualized and edited in the FigTree (Tree Figure Drawing Tool Version 1.4.3) software (http://tree.bio.ed.ac.uk/). The complete list of species and sequences used in the present work for phylogenetic analyses are listed in Supplementary Table 1. The corresponding sequences are reported at the end of the supplementary file, both the aligned sequences (with gaps) and the ungapped sequences. The sequences from strain SkelB that were annotated as *Skeletonema dohrnii* in MMETSP database were changed to *S. marinoi* after ribosomal sequence identification. Sequence ID were left unchanged. The phylogenetic tree was built on scale in the branch length (scale bar reported on Fig. 3). Sequences are identified by the species name, followed by their MMETSP ID (deprived of the taxon).

### *In-silico* TrotCOX protein structure modelling and comparison

We used Phyre^2^ (Protein Homology/analogY Recognition Engine V 2.0)^24^ to predict the possible structure of TrotCOX protein. In order to compare the TrotCOX predicted structure to known models of the peroxidase-cyclooxygenase family, the BtLPO (3BXI.pdb, 10.2210/pdb3bxi/pdb)^12,25^ and OaCOX1 structures were retrieved from the RCSB database (https://www.rcsb.org) and compared to TrotCOX using PyMOL Molecular Graphics System, Version 2.0 Schrödinger, LLC.

### Bioinformatic identification of the prostaglandin pathway

Pathway annotated as ‘prostaglandin biosynthesis’ was found among the second level pathways list generated within the Annocript pipeline annotation of the proteome from the *T. rotula* CCMP 1647 RNA-seq. Transcripts associated to the pathway were extrapolated from the total proteome annotation table^23^.

### Primer design and real time quantitative PCR

Candidate reference genes and genes of interest were selected considering the annotation of the peptides reported in the annotated transcriptome of *T. rotula* FE80 (CCMP1647)^23^.

Oligo sequences utilized are listed below^23^:

**Table:** 

Gene	Primer Sequence Forward Reverse	Length (bp)	Tm °C	GC content (%)	Amplicon Length (bp)	
COX-1 or PgG/Hs2	TCATCAAGGGAGGAGAATGG	CTTCCACCAAGAGCGAAGAC	20	58.4/60.5	50/55	170
PgEs2	TTCCAAACAGGGCAAGTTAC	TTGCACGAGACAGATTGGAG	20	56.4/58.4	45/50	183
PgFs	TCTCCCCTATCGAGGGTTCT	AGCTCCACTCTGCTATCC	20/18	60.5/56.3	55/56	114
TBP	CCTTCTTCAACCCCTCCACCAAC	GTTCGCTCATCCCACGTTTTCG	23/22	66.6/64.2	57/55	161
ACTIN	TCGGCCCTTGAGAAGAGTTTCG	GATGGTCTGGAAAGTGGAGTCC	22	64.2/64.2	55/55	147

Each sequence was initially tested by standard PCR in a 25 μL final volume with 2,5 μL of 10× PCR reaction buffer (Roche, Basel, Switzerland), 2,5 μL of 10 × 2 mM dNTP, 0.3 μL of 5 U/μL Taq (Roche, Basel, Switzerland), 1 μL 10 μΜ of each oligo, 1 μL of cDNA templates and nuclease-free water up to 25 μL. Standard PCR amplification program was used, i.e. 95 °C for 3 min, 40 cycles at 95 °C for 30 s, 53 °C 30 s, 72 °C for 30 s, and a final extension step at 72 °C for 7 min. Amplified PCR products were analysed by agarose gel electrophoresis and verified by sanger sequencing.

Reverse transcription-quantitative PCR (rt-qPCR) experiments were performed in MicroAmp Optical 384-Well reaction plate (Applied Biosystems, Foster City, CA, USA) with Optical Adhesive Covers (Applied Biosystems, Foster City, CA, USA) in a Viia7 Real Time PCR System (Applied Biosystem, Foster City, CA, USA). Five serial dilutions of mixed cDNAs were used to determine primer reaction efficiency^23^ using the formula: E = 10^−1/slope^. The PCR volume for each sample was 10 μL, with 5 μl of SensiFAST^TM^ SYBR^®^ Lo-ROX Kit (BIO_94020, Bioline), 1 μL of cDNA template (1 to 5 dilution each template) and 4 μL of 0.7 μM oligo mix (forward and reverse). Program reaction used was: 95 °C for 20 s, 40 cycles of 95 °C for 1 s and 60 °C for 20 s. The program was set to reveal the melting curve of each amplicon from 60 °C to 95 °C, and read every 0.5 °C. Single peaks for all genes confirmed gene-specific amplification and the absence of primer-dimers. All RT-qPCR reactions were carried out in triplicate to capture intra-assay variability. Each assay included three no-template negative controls for each primer pair.

The normalized expression levels of each gene of interest relative to the most stable reference genes^23^, actin and TBP, were calculated by using the Q-Gene tool^54^. Only TBP normalized values were reported in the main text and figure results. Relative expression ratios above two fold were considered significant^23^.

### Prostaglandin extraction

One µg of Prostaglandin E2-d4 (CAYMAN CHEMICAL, Michigan, USA) was added as internal standard to the media (100–200 mL) recovered from the cell culture, from day 3 to day 10, through a filtration step on 1.2 µm nitrocellulose filters (Millipore RAWP04700). Culture media were loaded onto pre-packed CHROMABOND® HR-X cartridges (500 mg/6 mL) previously activated with methanol (12 mL) and milliQ water (12 mL). After a preliminary desalting step with 12 mL of milliQ water, collection of the organic components was achieved by elution with 16 mL of methanol followed by 16 mL of methanol/dichloromethane (1:1). The two organic fractions were combined and dried under reduced pressure for LC-MS/MS analysis.

### LC-MS/MS analysis

*Thalassiosira rotula* water samples extracted as above, were re-suspended in 0.5 mL methanol and analyzed by tandem mass spectrometry in MRM (Multiple Reaction Monitoring) mode on a 4000 QTRAP® LC/MS/MS System (Applied Biosystems, Toronto, Canada), working in negative ion mode and coupled to a 1100 nanoHPLC system (Agilent Technologies, Waldbronn, Germany). Prostaglandins were separated by using a micro C18 column (10 cm ×1.0 mm, 5 µm). The mobile phase was generated by mixing eluent A (water, 0.1% Acetic Acid) and eluent B (acetonitrile/isopropanol 50/50) and the flow rate was 30 nL/min. Elution started at 20% B up to 95% B in 15 minutes. Tandem mass spectrometry was performed using a turbo ion spray source operating in negative mode, and the multiple reaction monitoring (MRM) mode was used for the selected analytes. Mass parameters (4000 qtrap AB science) were as follows: curtain gas 20 psi, GS 1/2 50/50 psi, ion spray voltage −5500 V, DP, −60 V; Dwell Time 25 ms and Temperature of 550 °C. Quantitative analysis was performed by monitoring a unique production^26^ arising from collision-induced fragmentation of the deprotonated selected parent compound after proper optimization of mass spectral parameters. After testing the scan mode including all the transitions, only the most intense transitions were chosen for each molecule.

### Statistical analysis

One-way ANOVA (α = 0.05) with Tukey’s post-hoc test was performed using GraphPad Prism6.0 (GraphPad Software Inc., San Diego, CA, USA). The analysis was performed to determine significant differences among the time points (days) considered during the *T. rotula* growth. Both qPCR amplification and LC-MS/MS data have been analysed.

## Supplementary information

Supplementary information.

Supplementary table 2.

## References

[1] Gladyshev MI, Sushchik NN, Makhutova ON (2013). Production of EPA and DHA in aquatic ecosystems and their transfer to the land. Prostaglandins Other Lipid Mediat..

[2] Serhan CN, Chiang N, Dalli J, Levy BD (2014). Lipid mediators in the resolution of inflammation. Cold Spring Harb. Perspect. Biol..

[3] Wiktorowska-Owczarek A, Berezińska M, Nowak JZ (2015). PUFAs: Structures, Metabolism and Functions. Adv. Clin. Exp. Med..

[4] Fullerton JN, Gilroy DW (2016). Resolution of inflammation: a new therapeutic frontier. Nat. Rev. Drug. Discov..

[5] Korbecki J, Baranowska-Bosiacka I, Gutowska I, Chlubek D (2014). Cyclooxygenase pathways. Acta Biochim. Pol..

[6] Versteeg HH, van Bergen en Henegouwen PM, van Deventer SJ, Peppelenbosch MP (1999). Cyclooxygenase-dependent signalling: molecular events and consequences. FEBS Lett..

[7] Di Costanzo, F., Di Dato, V., Ianora, A. & Romano, G. Prostaglandins in Marine Organisms: A Review. *Mar Drugs* **17**, (2019). 10.3390/md17070428 PMC6669704 31340503

[8] Clària J (2003). Cyclooxygenase-2 biology. Curr. Pharm. Des..

[9] Schultz JC, Appel HM (2004). Cross-Kingdom Cross-Talk: hormones shared by plants and their insect herbivores. Ecol..

[10] Ruan, J. *et al*. Jasmonic Acid Signaling Pathway in Plants. *Int. J. Mol. Sci.* **20**, (2019). 10.3390/ijms20102479 PMC6566436 31137463

[11] Groenewald EG, van der Westhuizen AJ (1997). Prostaglandins and related substances in plants. Botanical Rev..

[12] Zámocký M (2015). Independent evolution of four heme peroxidase superfamilies. Arch. Biochem. Biophysics.

[13] Varvas K (2009). Direct evidence of the cyclooxygenase pathway of prostaglandin synthesis in arthropods: genetic and biochemical characterization of two crustacean cyclooxygenases. Insect Biochem. Mol. Biol..

[14] Koljak R (2001). The basis of prostaglandin synthesis in coral: molecular cloning and expression of a cyclooxygenase from the Arctic soft coral *Gersemia fruticosa*. J. Biol. Chem..

[15] Di Dato V (2017). Animal-like prostaglandins in marine microalgae. ISME J..

[16] Flori, S. *et al*. Plastid thylakoid architecture optimizes photosynthesis in diatoms. *Nature Communications* **8** (2017). 10.1038/ncomms15885 PMC5481826 28631733

[17] Tanaka A (2015). Ultrastructure and membrane traffic during cell division in the marine pennate diatom *Phaeodactylum tricornutum*. Protist.

[18] De Martino A, Amato A, Bowler C (2009). Mitosis in diatoms: rediscovering an old model for cell division. BioEssays.

[19] Bowler C (2008). The *Phaeodactylum* genome reveals the evolutionary history of diatom genomes. Nat..

[20] Moustafa A (2009). Genomic footprints of a cryptic plastid endosymbiosis in diatoms. Sci..

[21] Matsuda Y, Hopkinson BM, Nakajima K, Dupont CL, Tsuji Y (2017). Mechanisms of carbon dioxide acquisition and CO _2_ sensing in marine diatoms: a gateway to carbon metabolism. Phil. Trans. R. Soc. B.

[22] Lupette J (2018). Non-enzymatic synthesis of bioactive isoprostanoids in the diatom *Phaeodactylum* following oxidative stress. Plant. Physiol..

[23] Di Dato V (2019). Unveiling the presence of biosynthetic pathways for bioactive compounds in the *Thalassiosira rotula* transcriptome. Sci. Rep..

[24] Kelley LA, Mezulis S, Yates CM, Wass MN, Sternberg MJE (2015). The Phyre2 web portal for protein modeling, prediction and analysis. Nat. Protoc..

[25] Singh AK (2009). Inhibition of lactoperoxidase by its own catalytic product: crystal structure of the hypothiocyanate-inhibited bovine lactoperoxidase at 2.3-Å resolution. Biophysical J..

[26] Wang, Y., Armando, A. M., Quehenberger, O., Yan, C. & Dennis, E. A. Comprehensive ultra-performance liquid chromatographic separation and mass spectrometric analysis of eicosanoid metabolites in human samples. *J. Chromatogr. A* **1359**, 60–69 (2014). 10.1016/j.chroma.2014.07.006 PMC4592635 25074422

[27] Yang, J., Schmelzer, K., Georgi, K. & Hammock, B. D. Quantitative profiling method for oxylipin metabolome by liquid chromatography electrospray ionization tandem mass spectrometry. *Anal. Chem.* **81**, 8085–8093 (2009). 10.1021/ac901282n PMC3290520 19715299

[28] Kroth PG (2008). A model for carbohydrate metabolism in the diatom *Phaeodactylum tricornutum* deduced from comparative whole genome analysis. PLoS ONE.

[29] Busseni G (2019). Meta-omics reveals genetic flexibility of diatom nitrogen transporters in response to environmental changes. Mol. Biol. Evolution.

[30] Vardi A, Thamatrakoln K, Bidle KD, Falkowski PG (2008). Diatom genomes come of age. Genome Biol..

[31] Bozarth A, Maier U-G, Zauner S (2008). Diatoms in biotechnology: modern tools and applications. Appl. Microbiol. Biotechnol..

[32] Zamocky, M., Jakopitsch, C., Furtmüller, P. G., Dunand, C. & Obinger, C. The peroxidase-cyclooxygenase superfamily: reconstructed evolution of critical enzymes of the innate immune system. *Proteins* **72**, 589–605 (2008). 10.1002/prot.21950 18247411

[33] Hamberg M, Ponce de Leon I, Rodriguez MJ, Castresana C (2005). Alpha-dioxygenases. Biochem. Biophys. Res. Commun..

[34] Smith WL, Garavito RM, DeWitt DL (1996). Prostaglandin endoperoxide H synthases (cyclooxygenases)-1 and -2. J. Biol. Chem..

[35] Sieber KB, Bromley RE, Dunning Hotopp JC (2017). Lateral gene transfer between prokaryotes and eukaryotes. Exp. Cell Res..

[36] Sorhannus U (2011). Evolution of antifreeze protein genes in the diatom genus *Fragilariopsis*: evidence for horizontal gene transfer, gene duplication and episodic diversifying selection. Evol. Bioinform. Online.

[37] Raymond JA, Kim HJ (2012). Possible role of horizontal gene transfer in the colonization of sea ice by algae. PLoS ONE.

[38] Diner RE (2017). Diatom centromeres suggest a mechanism for nuclear DNA acquisition. Proc. Natl. Acad. Sci. USA.

[39] Teng L (2017). Evolution and expansion of the prokaryote-like lipoxygenase family in the brown alga *Saccharina japonica*. Front. Plant. Sci..

[40] Orefice, I. Structural and functional analysis of lipoxygenase enzymes in marine diatoms. (The Open University of London, 2013).

[41] Gray TM, Matthews BW (1984). Intrahelical hydrogen bonding of serine, threonine and cysteine residues within α-helices and its relevance to membrane-bound proteins. J. Mol. Biol..

[42] Stonik V, Stonik I (2015). Low-molecular-weight metabolites from diatoms: structures, biological roles and biosynthesis. Mar. Drugs.

[43] Amin SA (2015). Interaction and signalling between a cosmopolitan phytoplankton and associated bacteria. Nat..

[44] Grossart H-P, Czub G, Simon M (2006). Algae-bacteria interactions and their effects on aggregation and organic matter flux in the sea. Environ. Microbiol..

[45] Martínez-Colón GJ, Moore BB (2018). Prostaglandin E2 as a regulator of immunity to pathogens. Pharmacology Therapeutics.

[46] Medina S (2012). Assessment of oxidative stress markers and prostaglandins after chronic training of triathletes. Prostaglandins Other Lipid Mediat..

[47] Idborg H (2014). Evaluation of urinary prostaglandin E2 metabolite as a biomarker in infants with fever due to viral infection. Prostaglandins Leukot. Essent. Fat. Acids.

[48] Kekatpure VD (2016). Elevated levels of urinary PGE-M are found in tobacco users and indicate a poor prognosis for oral squamous cell carcinoma patients. Cancer Prev. Res..

[49] Aoki T, Narumiya S (2012). Prostaglandins and chronic inflammation. Trends Pharmacol. Sci..

[50] Meyer N, Rettner J, Werner M, Werz O, Pohnert G (2018). Algal oxylipins mediate the resistance of diatoms against algicidal bacteria. Mar. Drugs.

[51] Guillard, R. R. L. Culture of phytoplankton for feeding marine invertebrates. In *Culture of Marine Invertebrate Animals* (eds. Smith, W. L. & Chanley, M. H.) 29–60 (Springer US, 1975).

[52] Hall T (1999). BioEdit: a user-friendly biological sequence alignment editor and analysis program for Windows 95/98/NT. Nucleic Acids Symposium Ser..

[53] Kumar S, Stecher G, Li M, Knyaz C, Tamura K (2018). MEGA X: Molecular Evolutionary Genetics Analysis across Computing Platforms. Mol. Biol. Evol..

[54] Simon P (2003). Q-Gene: processing quantitative real-time RT-PCR data. Bioinforma..

[55] Schneider, T. D. & Stephens, R. M. Sequence logos: a new way to display consensus sequences. *Nucleic Acids Research* **18**(20), 6097–6100 (1990). 10.1093/nar/18.20.6097 PMC332411 2172928

