# Supplementary material for: Variation in prostaglandin metabolism during growth of the diatom Thalassiosira rotula
Source: Sci Rep. 2020 Mar 25;10:5374. doi: 10.1038/s41598-020-61967-3 (PMC7096440; doi:10.1038/s41598-020-61967-3)
Supplement: Supplementary file 1 — Supplementary information. [file 41598_2020_61967_MOESM1_ESM.pdf]

Variation in prostaglandin metabolism during growth of the diatom *Thalassiosira rotula*

Valeria Di Dato, Roberta Barbarinaldi, Alberto Amato, Federica Di Costanzo, Carolina Fontanarosa, Anna Perna, Angela Amoresano, Francesco Esposito, Adele Cutignano, Adrianna Ianora, Giovanna Romano

Supplementary Information

Supplementary information content:

- ✓ Supplementary figure 1: 4

  Phyre^{2} best hits structures from TrotCOX protein blast.

- ✓ Supplementary figure 2: 10

  Phyre^{2} alignment between *Thalassiosira rotula* (TrotCOX) and *Bos taurus* lactoperoxidase (BtLPO).

- ✓ Supplementary figure 3: 11

  Phyre^{2} alignment between *Thalassiosira rotula* (TrotCOX) and *Ovis aries* cycloxygenase (OaCOX).

- ✓ Supplementary figure 4: 12

  Alignment of the diatoms sequences used in the phylogenetic analysis.

- ✓ Supplementary table 1: 13

  List of species and sequences ID used in the phylogenetic analysis.

- ✓ External standard calibration method for PG quantization. 15

- ✓ Supplementary figure 5: 17

  Calibration curve of deuterated prostaglandin E2 (PGE2-d4) used as internal standard.

- ✓ Supplementary figure 6: 18

  Calibration curve of prostaglandin E2 (PGE2) used as standard.

- ✓ Supplementary figure 7: 19

  Calibration curve of prostaglandin B2 (PGB2) used as standard.

- ✓ Supplementary figure 8: 20

Calibration curve of 15-Deoxy-Delta-12,14-prostaglandin J2 (15dPGJ2) used as standard.

- ✓ Supplementary figure 9: 21

TIC chromatogram (A) of a representative sample and XIC chromatogram (B) of PGEM (327 m/z precursor ion; 291 m/z and 309 m/z daughter ion).

- ✓ Supplementary table 3: 22

List of PGs identified in the culture medium with their LC-MS/MS parameters.

- ✓ Aligned sequences, with gap, used in the phylogenetic analysis: 23
- ✓ Sequences used in the phylogenetic analysis, without gap 38
- ✓ References 48

Supplementary figure 1. Phyre2[1] best hits structures from TrotCOX protein blast.

Phyre²

Email	alberto.amato@cea.fr
Description	Thalassiosira_rotula_TR754_c0_g1_i1_m_3431
Date	Thu Nov 7 16:33:40 GMT 2019
Unique Job ID	ca99ec1885b17cd9

Detailed template information

#	Template	Alignment Coverage	3D Model	Confidence	% i.d.	Template Information
1	c2gjmA_	Alignment	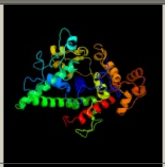	100.0	19	**PDB header:**oxidoreductase **Chain:** A: **PDB Molecule:**lactoperoxidase; **PDBTitle:** crystal structure of buffalo lactoperoxidase at 2.75a resolution
2	c2oyuP_	Alignment	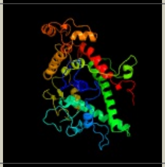	100.0	28	**PDB header:**oxidoreductase **Chain:** P: **PDB Molecule:**prostaglandin g/h synthase 1; **PDBTitle:** indomethacin-(s)-alpha-ethyl-ethanolamide bound to cyclooxygenase-1
3	c1pggB_	Alignment	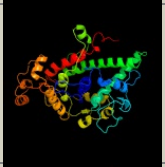	100.0	28	**PDB header:**oxidoreductase **Chain:** B: **PDB Molecule:**prostaglandin h2 synthase-1; **PDBTitle:** prostaglandin h2 synthase-1 complexed with 1-(4-iodobenzoyl)-5-2 methoxy-2-methylindole-3-acetic acid (iodoindomethacin), trans model
4	c1ht8B_	Alignment	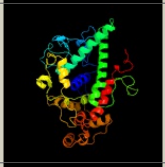	100.0	28	**PDB header:**oxidoreductase **Chain:** B: **PDB Molecule:**prostaglandin h2 synthase-1; **PDBTitle:** the 2.7 angstrom resolution model of ovine cox-1 complexed with2 alclofenac
5	d1q4ga1	Alignment	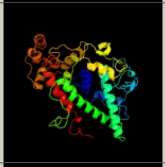	100.0	28	**Fold:**Heme-dependent peroxidases **Superfamily:**Heme-dependent peroxidases **Family:**Myeloperoxidase-like
6	c6ercA_	Alignment	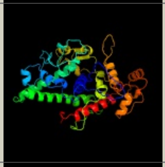	100.0	26	**PDB header:**oxidoreductase **Chain:** A: **PDB Molecule:**peroxinectin a; **PDBTitle:** peroxidase a from dictyostelium discoideum (ddpoxa)
7	c5mfaA_	Alignment	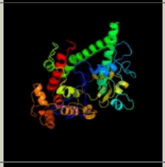	100.0	20	**PDB header:**oxidoreductase **Chain:** A: **PDB Molecule:**myeloperoxidase; **PDBTitle:** crystal structure of human promyeloperoxidase (prompo)
8	c1ddxA_	Alignment	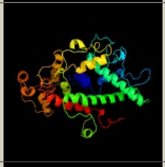	100.0	27	**PDB header:**oxidoreductase **Chain:** A: **PDB Molecule:**protein (prostaglandin h2 synthase-2); **PDBTitle:** crystal structure of a mixture of arachidonic acid and prostaglandin2 bound to the cyclooxygenase active site of cox-2: prostaglandin3 structure
9	c3pghD_	Alignment	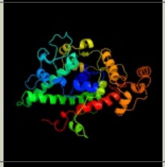	100.0	27	**PDB header:**oxidoreductase **Chain:** D: **PDB Molecule:**cyclooxygenase-2; **PDBTitle:** cyclooxygenase-2 (prostaglandin synthase-2) complexed with a non-2 selective inhibitor, flurbiprofen
10	d1cvua1	Alignment	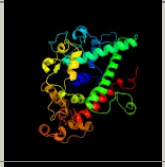	100.0	27	**Fold:**Heme-dependent peroxidases **Superfamily:**Heme-dependent peroxidases **Family:**Myeloperoxidase-like
11	c1d2vD_	Alignment	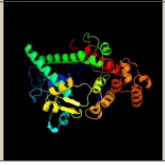	100.0	20	**PDB header:**oxidoreductase **Chain:** D: **PDB Molecule:**myeloperoxidase; **PDBTitle:** crystal structure of bromide-bound human myeloperoxidase isoform c at2 ph 5.5

12	c4hhsA_	Alignment	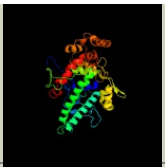	100.0	19	**PDB header:**oxidoreductase<br>**Chain:** A: **PDB Molecule:**alpha-dioxygenase;<br>**PDBTitle:** crystal structure of fatty acid alpha-dioxygenase (arabidopsis2 thaliana)
13	c4kvjA_	Alignment	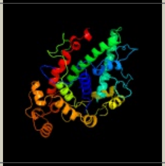	100.0	19	**PDB header:**oxidoreductase<br>**Chain:** A: **PDB Molecule:**fatty acid alpha-oxidase;<br>**PDBTitle:** crystal structure of oryza sativa fatty acid alpha-dioxygenase with2 hydrogen peroxide
14	c5gvrA_	Alignment	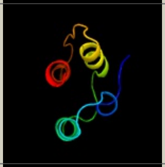	36.3	17	**PDB header:**hydrolase<br>**Chain:** A: **PDB Molecule:**probable atp-dependent rna helicase ddx41;<br>**PDBTitle:** crystal structure of the ddx41 dead domain in an apo closed form
15	c3brcA_	Alignment	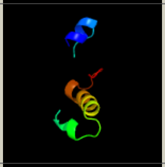	35.0	18	**PDB header:**structural genomics, unknown function<br>**Chain:** A: **PDB Molecule:**conserved protein of unknown function;<br>**PDBTitle:** crystal structure of a conserved protein of unknown function from2 methanobacterium thermoautotrophicum
16	c1vk5A_	Alignment	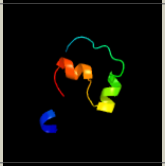	33.5	20	**PDB header:**structural genomics, unknown function<br>**Chain:** A: **PDB Molecule:**expressed protein;<br>**PDBTitle:** x-ray structure of gene product from arabidopsis thaliana at3g22680
17	d1vk5a_	Alignment	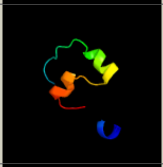	33.5	20	**Fold:**Hypothetical protein At3g22680<br>**Superfamily:**Hypothetical protein At3g22680<br>**Family:**Hypothetical protein At3g22680
18	d1u7ka_	Alignment	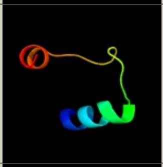	28.8	33	**Fold:**Retrovirus capsid protein, N-terminal core domain<br>**Superfamily:**Retrovirus capsid protein, N-terminal core domain<br>**Family:**Retrovirus capsid protein, N-terminal core domain
19	c3fe2B_	Alignment	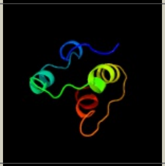	22.4	13	**PDB header:**hydrolase<br>**Chain:** B: **PDB Molecule:**probable atp-dependent rna helicase ddx5;<br>**PDBTitle:** human dead-box rna helicase ddx5 (p68), conserved domain i in complex2 with adp
20	c4px9C_	Alignment	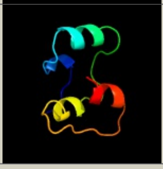	21.3	12	**PDB header:**translation, rna binding protein<br>**Chain:** C: **PDB Molecule:**atp-dependent rna helicase ddx3x;<br>**PDBTitle:** dead-box rna helicase ddx3x domain 1 with n-terminal atp-binding loop
21	c3evyB_	Alignment	not modelled	17.7	9	**PDB header:**hydrolase<br>**Chain:** B: **PDB Molecule:**putative type i restriction enzyme r protein;<br>**PDBTitle:** crystal structure of a fragment of a putative type i restriction2 enzyme r protein from bacteroides fragilis
22	d1ou9a_	Alignment	not modelled	17.3	21	**Fold:**SspB-like<br>**Superfamily:**SspB-like<br>**Family:**Stringent starvation protein B, SspB
23	c2noxP_	Alignment	not modelled	17.3	14	**PDB header:**oxidoreductase<br>**Chain:** P: **PDB Molecule:**tryptophan 2,3-dioxygenase;<br>**PDBTitle:** crystal structure of tryptophan 2,3-dioxygenase from ralstonia2 metallidurans
24	c6hwwA_	Alignment	not modelled	16.6	33	**PDB header:**viral protein<br>**Chain:** A: **PDB Molecule:**putative gag polyprotein;<br>**PDBTitle:** immature mlv capsid hexamer structure in intact virus particles
25	d1zszc1	Alignment	not modelled	16.5	16	**Fold:**SspB-like<br>**Superfamily:**SspB-like<br>**Family:**Stringent starvation protein B, SspB
26	c6hwyB_	Alignment	not modelled	15.9	33	**PDB header:**viral protein<br>**Chain:** B: **PDB Molecule:**putative gag polyprotein;<br>**PDBTitle:** mature mlv capsid pentamer structure in intact virus particles
27	c3fhcB_	Alignment	not modelled	15.6	9	**PDB header:**transport protein/hydrolase<br>**Chain:** B: **PDB Molecule:**atp-dependent rna helicase ddx19b;<br>**PDBTitle:** crystal structure of human dbp5 in complex with nup214
28	d1or7b2	Alignment	not modelled	15.0	32	**Fold:**Sigma2 domain of RNA polymerase sigma factors<br>**Superfamily:**Sigma2 domain of RNA polymerase sigma factors<br>**Family:**Sigma2 domain of RNA polymerase sigma factors

29	c4cmxB_	Alignment	not modelled	14.8	14	**PDB header:**nuclear protein<br>**Chain:** B: **PDB Molecule:**rv3378c;<br>**PDBTitle:** crystal structure of rv3378c
30	d1g1xc_	Alignment	not modelled	14.3	25	**Fold:**DNA/RNA-binding 3-helical bundle<br>**Superfamily:**Ribosomal protein S18<br>**Family:**Ribosomal protein S18
31	c4qicB_	Alignment	not modelled	14.2	18	**PDB header:**signaling protein/dna binding protein<br>**Chain:** B: **PDB Molecule:**anti-sigma factor nepr;<br>**PDBTitle:** co-crystal structure of anti-anti-sigma factor phyr complexed with2 anti-sigma factor nepr from bartonella quintana
32	d1mn3a_	Alignment	not modelled	13.8	14	**Fold:**RuvA C-terminal domain-like<br>**Superfamily:**UBA-like<br>**Family:**CUE domain
33	d1ou8a_	Alignment	not modelled	12.8	21	**Fold:**SspB-like<br>**Superfamily:**SspB-like<br>**Family:**Stringent starvation protein B, SspB
34	c2c2xB_	Alignment	not modelled	12.6	14	**PDB header:**oxidoreductase<br>**Chain:** B: **PDB Molecule:**methylenetetrahydrofolate dehydrogenase-<br>**PDBTitle:** three dimensional structure of bifunctional methylenetetrahydrofolate2 dehydrogenase-cyclohydrolase from mycobacterium tuberculosis
35	c1fcuA_	Alignment	not modelled	12.6	36	**PDB header:**hydrolase<br>**Chain:** A: **PDB Molecule:**hyaluronoglucosaminidase;<br>**PDBTitle:** crystal structure (trigonal) of bee venom hyaluronidase
36	d1fcqa_	Alignment	not modelled	12.5	36	**Fold:**TIM beta/alpha-barrel<br>**Superfamily:**(Trans)glycosidases<br>**Family:**Bee venom hyaluronidase
37	c3fmpD_	Alignment	not modelled	12.2	9	**PDB header:**oncoprotein/hydrolase<br>**Chain:** D: **PDB Molecule:**atp-dependent rna helicase ddx19b;<br>**PDBTitle:** crystal structure of the nucleoporin nup214 in complex with the dead-2 box helicase ddx19
38	d1yfna1	Alignment	not modelled	11.9	21	**Fold:**SspB-like<br>**Superfamily:**SspB-like<br>**Family:**Stringent starvation protein B, SspB
39	c2b19A_	Alignment	not modelled	11.5	42	**PDB header:**neuropeptide<br>**Chain:** A: **PDB Molecule:**neuropeptide k;<br>**PDBTitle:** solution structure of mammalian tachykinin peptide,2 neuropeptide k
40	c3rfaB_	Alignment	not modelled	11.0	13	**PDB header:**oxidoreductase<br>**Chain:** B: **PDB Molecule:**ribosomal rna large subunit methyltransferase n;<br>**PDBTitle:** x-ray structure of rlmn from escherichia coli in complex with s-2 adenosylmethionine
41	c2kneB_	Alignment	not modelled	10.8	32	**PDB header:**metal transport<br>**Chain:** B: **PDB Molecule:**atpase, ca++ transporting, plasma membrane 4;<br>**PDBTitle:** calmodulin wraps around its binding domain in the plasma2 membrane ca2+ pump anchored by a novel 18-1 motif
42	d1upka_	Alignment	not modelled	10.6	12	**Fold:**alpha-alpha superhelix<br>**Superfamily:**ARM repeat<br>**Family:**Mo25 protein
43	c3rfaA_	Alignment	not modelled	10.2	13	**PDB header:**oxidoreductase<br>**Chain:** A: **PDB Molecule:**ribosomal rna large subunit methyltransferase n;<br>**PDBTitle:** x-ray structure of rlmn from escherichia coli in complex with s-2 adenosylmethionine
44	d2bmfa1	Alignment	not modelled	10.1	21	**Fold:**P-loop containing nucleoside triphosphate hydrolases<br>**Superfamily:**P-loop containing nucleoside triphosphate hydrolases<br>**Family:**RNA helicase
45	d2qalr1	Alignment	not modelled	10.1	29	**Fold:**DNA/RNA-binding 3-helical bundle<br>**Superfamily:**Ribosomal protein S18<br>**Family:**Ribosomal protein S18
46	c3df1R_	Alignment	not modelled	10.1	29	**PDB header:**ribosome<br>**Chain:** R: **PDB Molecule:**30s ribosomal protein s18;<br>**PDBTitle:** crystal structure of the bacterial ribosome from escherichia coli in2 complex with hygromycin b. this file contains the 30s subunit of the3 first 70s ribosome, with hygromycin b bound. the entire crystal4 structure contains two 70s ribosomes.
47	d1ho8a_	Alignment	not modelled	9.9	21	**Fold:**alpha-alpha superhelix<br>**Superfamily:**ARM repeat<br>**Family:**Regulatory subunit H of the V-type ATPase
48	c3cuzA_	Alignment	not modelled	9.9	9	**PDB header:**transferase<br>**Chain:** A: **PDB Molecule:**malate synthase a;<br>**PDBTitle:** atomic resolution structures of escherichia coli and2 bacillis anthracis malate synthase a: comparison with3 isoform g and implications for structure based drug design
49	c2g2bA_	Alignment	not modelled	9.9	7	**PDB header:**immune system<br>**Chain:** A: **PDB Molecule:**allograft inflammatory factor 1;<br>**PDBTitle:** nmr structure of the human allograft inflammatory factor 1
50	c5gjuA_	Alignment	not modelled	9.5	26	**PDB header:**hydrolase<br>**Chain:** A: **PDB Molecule:**atp-dependent rna helicase dead;<br>**PDBTitle:** dead-box rna helicase
51	c5cofA_	Alignment	not modelled	9.4	11	**PDB header:**unknown function<br>**Chain:** A: **PDB Molecule:**uncharacterized protein;<br>**PDBTitle:** crystal structure of uncharacterised protein q1r1x2 from escherichia2 coli uti89
52	c5o5jR_	Alignment	not modelled	9.3	31	**PDB header:**ribosome<br>**Chain:** R: **PDB Molecule:**30s ribosomal protein s18 2;<br>**PDBTitle:** structure of the 30s small ribosomal subunit from mycobacterium2 smegmatis
53	c3a0bT_	Alignment	not modelled	8.9	20	**PDB header:**electron transport<br>**Chain:** T: **PDB Molecule:**photosystem ii reaction center protein t;<br>**PDBTitle:** crystal structure of br-substituted photosystem ii complex

54	c5mx2T_	Alignment	not modelled	8.2	18	**PDB header:**oxidoreductase **Chain:** T: **PDB Molecule:**photosystem ii reaction center protein t; **PDBTitle:** photosystem ii depleted of the mn4cao5 cluster at 2.55 a resolution
55	c2iv1J_	Alignment	not modelled	8.2	17	**PDB header:**lyase **Chain:** J: **PDB Molecule:**cyanate hydratase; **PDBTitle:** site directed mutagenesis of key residues involved in the catalytic2 mechanism of cyanase
56	d1rfya_	Alignment	not modelled	8.2	24	**Fold:**Long alpha-hairpin **Superfamily:**Transcriptional repressor TraM **Family:**Transcriptional repressor TraM
57	c5aj3R_	Alignment	not modelled	8.0	6	**PDB header:**ribosome **Chain:** R: **PDB Molecule:**mitoribosomal protein bs18m, mrps18c; **PDBTitle:** structure of the small subunit of the mammalian mitoribosome
58	c2c0zA_	Alignment	not modelled	7.9	22	**PDB header:**isomerase **Chain:** A: **PDB Molecule:**novw; **PDBTitle:** the 1.6 a resolution crystal structure of novw: a 4-keto-6-2 deoxy sugar epimerase from the novobiocin biosynthetic3 gene cluster of streptomyces spheroides
59	c3j0xU_	Alignment	not modelled	7.8	31	**PDB header:**ribosome **Chain:** U: **PDB Molecule:**30s ribosomal protein s18; **PDBTitle:** structural characterization of mrna-trna translocation intermediates2 (30s ribosome of class 4b of the six classes)
60	d1wrka1	Alignment	not modelled	7.8	7	**Fold:**EF Hand-like **Superfamily:**EF-hand **Family:**Calmodulin-like
61	c1upiA_	Alignment	not modelled	7.8	16	**PDB header:**epimerase **Chain:** A: **PDB Molecule:**dtdp-4-dehydrorhamnose 3,5-epimerase; **PDBTitle:** mycobacterium tuberculosis rmlc epimerase (rv3465)
62	c5b66T_	Alignment	not modelled	7.7	18	**PDB header:**electron transport, photosynthesis **Chain:** T: **PDB Molecule:**photosystem ii reaction center protein t; **PDBTitle:** crystal structure analysis of photosystem ii complex
63	c3bbnR_	Alignment	not modelled	7.7	27	**PDB header:**ribosome **Chain:** R: **PDB Molecule:**ribosomal protein s18; **PDBTitle:** homology model for the spinach chloroplast 30s subunit fitted to 9.4a2 cryo-em map of the 70s chlororibosome.
64	c5gtht_	Alignment	not modelled	7.6	18	**PDB header:**photosynthesis **Chain:** T: **PDB Molecule:**photosystem ii reaction center protein t; **PDBTitle:** native xfel structure of photosystem ii (dark dataset)
65	c5ws5t_	Alignment	not modelled	7.6	18	**PDB header:**photosynthesis **Chain:** T: **PDB Molecule:**photosystem ii reaction center protein t; **PDBTitle:** native xfel structure of photosystem ii (preflash dark dataset)
66	c5ws6t_	Alignment	not modelled	7.6	18	**PDB header:**photosynthesis **Chain:** T: **PDB Molecule:**photosystem ii reaction center protein t; **PDBTitle:** native xfel structure of photosystem ii (preflash two-flash dataset
67	c5ws5T_	Alignment	not modelled	7.6	18	**PDB header:**photosynthesis **Chain:** T: **PDB Molecule:**photosystem ii reaction center protein t; **PDBTitle:** native xfel structure of photosystem ii (preflash dark dataset)
68	c5gthT_	Alignment	not modelled	7.6	18	**PDB header:**photosynthesis **Chain:** T: **PDB Molecule:**photosystem ii reaction center protein t; **PDBTitle:** native xfel structure of photosystem ii (dark dataset)
69	c3wu2T_	Alignment	not modelled	7.6	18	**PDB header:**electron transport, photosynthesis **Chain:** T: **PDB Molecule:**photosystem ii reaction center protein t; **PDBTitle:** crystal structure analysis of photosystem ii complex
70	c5h2fT_	Alignment	not modelled	7.6	18	**PDB header:**photosynthesis **Chain:** T: **PDB Molecule:**photosystem ii reaction center protein t; **PDBTitle:** crystal structure of the psbm-deletion mutant of photosystem ii
71	c5kait_	Alignment	not modelled	7.6	18	**PDB header:**electron transport **Chain:** T: **PDB Molecule:**photosystem ii reaction center protein t; **PDBTitle:** nh3-bound rt xfel structure of photosystem ii 500 ms after the 2nd2 illumination (2f) at 2.8 a resolution
72	c5h2ft_	Alignment	not modelled	7.6	18	**PDB header:**photosynthesis **Chain:** T: **PDB Molecule:**photosystem ii reaction center protein t; **PDBTitle:** crystal structure of the psbm-deletion mutant of photosystem ii
73	c4ub8t_	Alignment	not modelled	7.6	18	**PDB header:**electron transport, photosynthesis **Chain:** T: **PDB Molecule:**photosystem ii reaction center protein t; **PDBTitle:** native structure of photosystem ii (dataset-2) by a femtosecond x-ray2 laser
74	c3wu2t_	Alignment	not modelled	7.6	18	**PDB header:**electron transport, photosynthesis **Chain:** T: **PDB Molecule:**photosystem ii reaction center protein t; **PDBTitle:** crystal structure analysis of photosystem ii complex
75	c5gtiT_	Alignment	not modelled	7.6	18	**PDB header:**photosynthesis **Chain:** T: **PDB Molecule:**photosystem ii reaction center protein t; **PDBTitle:** native xfel structure of photosystem ii (two flash dataset)
76	c5v2cT_	Alignment	not modelled	7.6	18	**PDB header:**electron transport, photosynthesis **Chain:** T: **PDB Molecule:**photosystem ii reaction center protein t; **PDBTitle:** re-refinement of crystal structure of photosystem ii complex
77	c5gtit_	Alignment	not modelled	7.6	18	**PDB header:**photosynthesis **Chain:** T: **PDB Molecule:**photosystem ii reaction center protein t; **PDBTitle:** native xfel structure of photosystem ii (two flash dataset)
78	c5v2ct_	Alignment	not modelled	7.6	18	**PDB header:**electron transport, photosynthesis **Chain:** T: **PDB Molecule:**photosystem ii reaction center protein t; **PDBTitle:** re-refinement of crystal structure of photosystem ii complex
79	c6jlpt_	Alignment	not modelled	7.6	18	**PDB header:**photosynthesis **Chain:** T: **PDB Molecule:**photosystem ii reaction center protein t; **PDBTitle:** xfel structure of cyanobacterial photosystem ii (3f state, dataset2)
						**PDB header:**photosynthesis

80	c6jlpT_	Alignment	not modelled	7.6	18	**Chain:** T: **PDB Molecule:**photosystem ii reaction center protein t; **PDBTitle:** xfel structure of cyanobacterial photosystem ii (3f state, dataset2)
81	c6jlmT_	Alignment	not modelled	7.6	18	**PDB header:**photosynthesis **Chain:** T: **PDB Molecule:**photosystem ii reaction center protein t; **PDBTitle:** xfel structure of cyanobacterial photosystem ii (dark state, dataset2)
82	c6jlkT_	Alignment	not modelled	7.6	18	**PDB header:**photosynthesis **Chain:** T: **PDB Molecule:**photosystem ii reaction center protein t; **PDBTitle:** xfel structure of cyanobacterial photosystem ii (1f state, dataset1)
83	c6jloT_	Alignment	not modelled	7.6	18	**PDB header:**photosynthesis **Chain:** T: **PDB Molecule:**photosystem ii reaction center protein t; **PDBTitle:** xfel structure of cyanobacterial photosystem ii (2f state, dataset2)
84	c6jljT_	Alignment	not modelled	7.6	18	**PDB header:**photosynthesis **Chain:** T: **PDB Molecule:**photosystem ii reaction center protein t; **PDBTitle:** xfel structure of cyanobacterial photosystem ii (dark state, dataset1)
85	c6jllT_	Alignment	not modelled	7.6	18	**PDB header:**photosynthesis **Chain:** T: **PDB Molecule:**photosystem ii reaction center protein t; **PDBTitle:** xfel structure of cyanobacterial photosystem ii (2f state, dataset1)
86	c6jlot_	Alignment	not modelled	7.6	18	**PDB header:**photosynthesis **Chain:** T: **PDB Molecule:**photosystem ii reaction center protein t; **PDBTitle:** xfel structure of cyanobacterial photosystem ii (2f state, dataset2)
87	c6jlnt_	Alignment	not modelled	7.6	18	**PDB header:**photosynthesis **Chain:** T: **PDB Molecule:**photosystem ii reaction center protein t; **PDBTitle:** xfel structure of cyanobacterial photosystem ii (1f state, dataset2)
88	c6jlnT_	Alignment	not modelled	7.6	18	**PDB header:**photosynthesis **Chain:** T: **PDB Molecule:**photosystem ii reaction center protein t; **PDBTitle:** xfel structure of cyanobacterial photosystem ii (1f state, dataset2)
89	c6jllt_	Alignment	not modelled	7.6	18	**PDB header:**photosynthesis **Chain:** T: **PDB Molecule:**photosystem ii reaction center protein t; **PDBTitle:** xfel structure of cyanobacterial photosystem ii (2f state, dataset1)
90	c6jlkt_	Alignment	not modelled	7.6	18	**PDB header:**photosynthesis **Chain:** T: **PDB Molecule:**photosystem ii reaction center protein t; **PDBTitle:** xfel structure of cyanobacterial photosystem ii (1f state, dataset1)
91	c6jlmt_	Alignment	not modelled	7.6	18	**PDB header:**photosynthesis **Chain:** T: **PDB Molecule:**photosystem ii reaction center protein t; **PDBTitle:** xfel structure of cyanobacterial photosystem ii (dark state, dataset2)
92	c6jljt_	Alignment	not modelled	7.6	18	**PDB header:**photosynthesis **Chain:** T: **PDB Molecule:**photosystem ii reaction center protein t; **PDBTitle:** xfel structure of cyanobacterial photosystem ii (dark state, dataset1)
93	c5tisT_	Alignment	not modelled	7.6	18	**PDB header:**photosynthesis **Chain:** T: **PDB Molecule:**photosystem ii reaction center protein t; **PDBTitle:** room temperature xfel structure of the native, doubly-illuminated2 photosystem ii complex
94	c5zznt_	Alignment	not modelled	7.6	18	**PDB header:**electron transport **Chain:** T: **PDB Molecule:**photosystem ii reaction center protein t; **PDBTitle:** crystal structure of photosystem ii from an sqdg-deficient mutant of2 thermosynechococcus elongatus
95	c5kaiT_	Alignment	not modelled	7.6	18	**PDB header:**electron transport **Chain:** T: **PDB Molecule:**photosystem ii reaction center protein t; **PDBTitle:** nh3-bound rt xfel structure of photosystem ii 500 ms after the 2nd2 illumination (2f) at 2.8 a resolution
96	c5kafT_	Alignment	not modelled	7.6	18	**PDB header:**electron transport **Chain:** T: **PDB Molecule:**photosystem ii reaction center protein t; **PDBTitle:** rt xfel structure of photosystem ii in the dark state at 3.0 a2 resolution
97	c4ub6T_	Alignment	not modelled	7.6	18	**PDB header:**electron transport, photosynthesis **Chain:** T: **PDB Molecule:**photosystem ii reaction center protein t; **PDBTitle:** native structure of photosystem ii (dataset-1) by a femtosecond x-ray2 laser
98	c5zznT_	Alignment	not modelled	7.6	18	**PDB header:**electron transport **Chain:** T: **PDB Molecule:**photosystem ii reaction center protein t; **PDBTitle:** crystal structure of photosystem ii from an sqdg-deficient mutant of2 thermosynechococcus elongatus
99	c4il6t_	Alignment	not modelled	7.6	18	**PDB header:**electron transport **Chain:** T: **PDB Molecule:**photosystem ii reaction center protein t; **PDBTitle:** structure of sr-substituted photosystem ii

Supplementary figure 2. Phyre2[1] alignment between *Thalassiosira rotula* (TrotCOX) and *Bos taurus* lactoperoxidase (BtLPO).

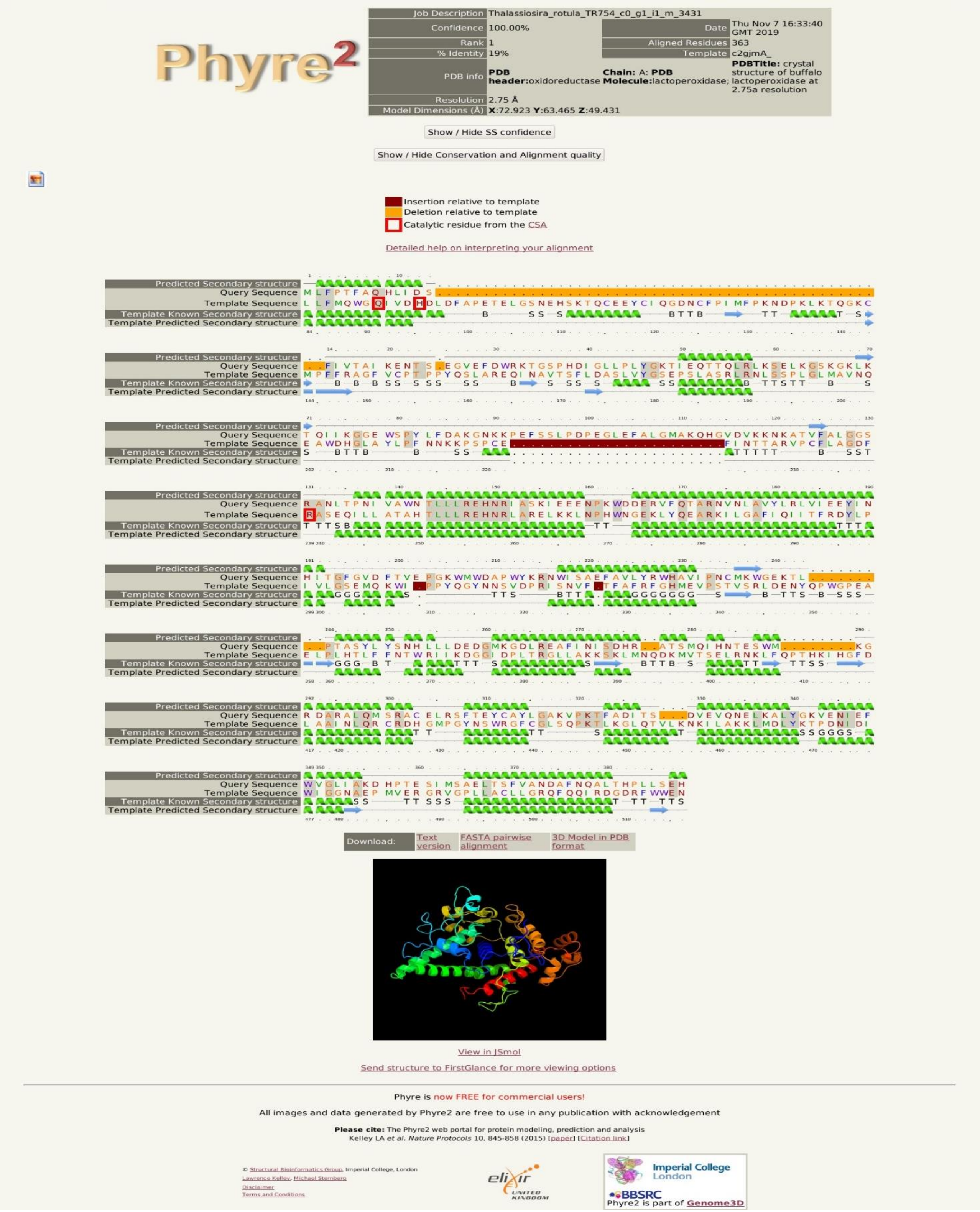

Supplementary figure 3. Phyre2[1] alignment between *Thalassiosira rotula* (TrotCOX) and *Ovis aries* cyclooxygenase (OaCOX).

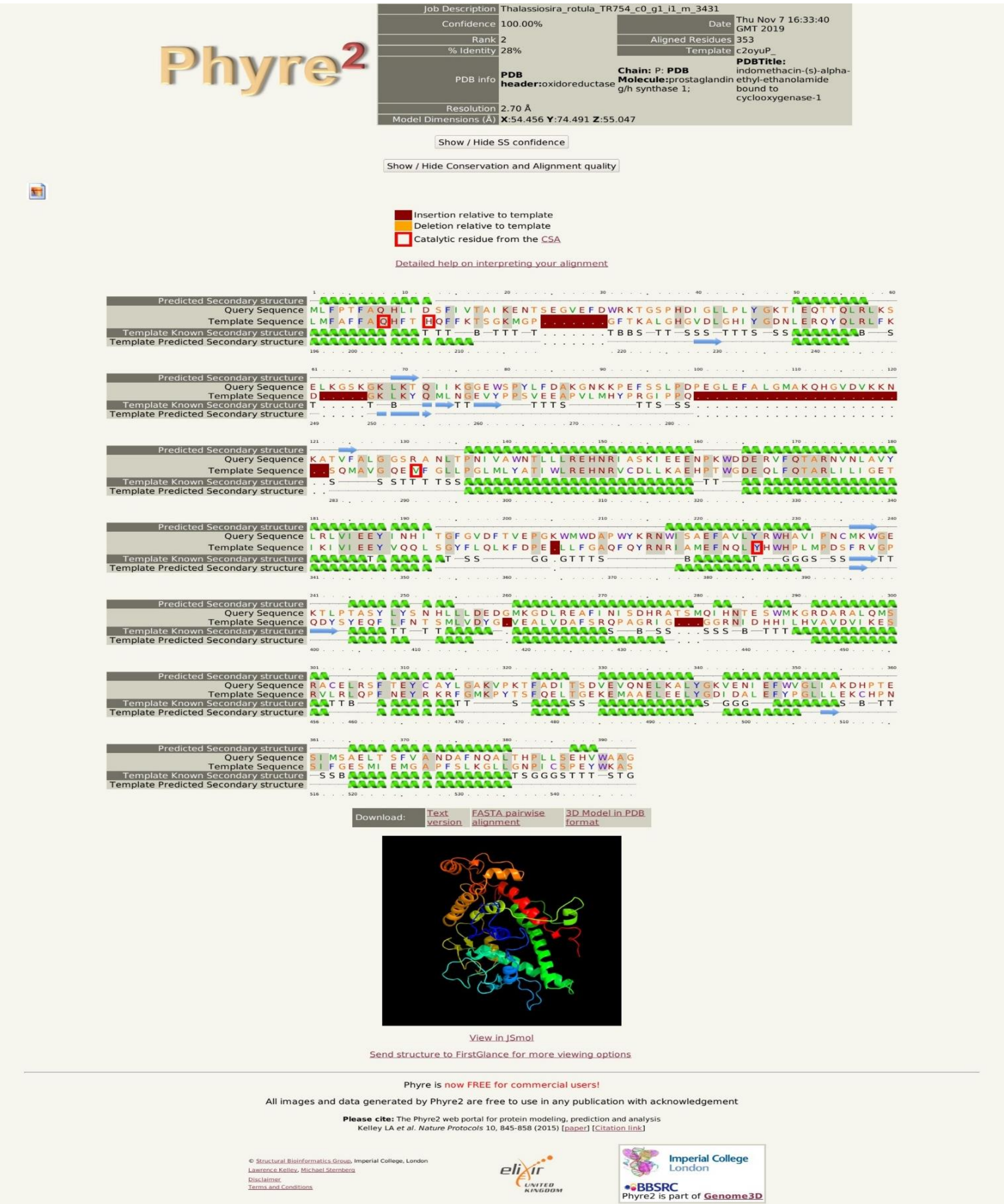

Supplementary figure 4. Alignment of the diatoms sequences used in the phylogenetic analysis (Figure 3, diatoms clade).

In red box the *T. rotula* sequence. The red arrow indicate the glutamate residue transformed in leucine in diatoms (yellow circle).

BtLPO = *Bos taurus* LPO; A.sub = *Aulacoseira subarctica* strain; S.cos = *Skeletonema costatum*; S.mar = *Skeletonema marinoi*; D.con = *Detonula confervacea*; T.fra = *Thalassionema frauenfeldii*; P.are = *Pseudo-nitzschia arenisensis*; C.men = *Cyclotella meneghiniana*; Odont = *Odontella*; T.rot = *Thalassiosira rotula*; C.cur = *Chaetoceros curvisetus*; C.deb = *Chaetoceros debilis*; C.aff = *Chaetoceros affinis*; A.gla = *Asterionellopsis glacialis*; C.neo = *Chaetoceros neogracile*.

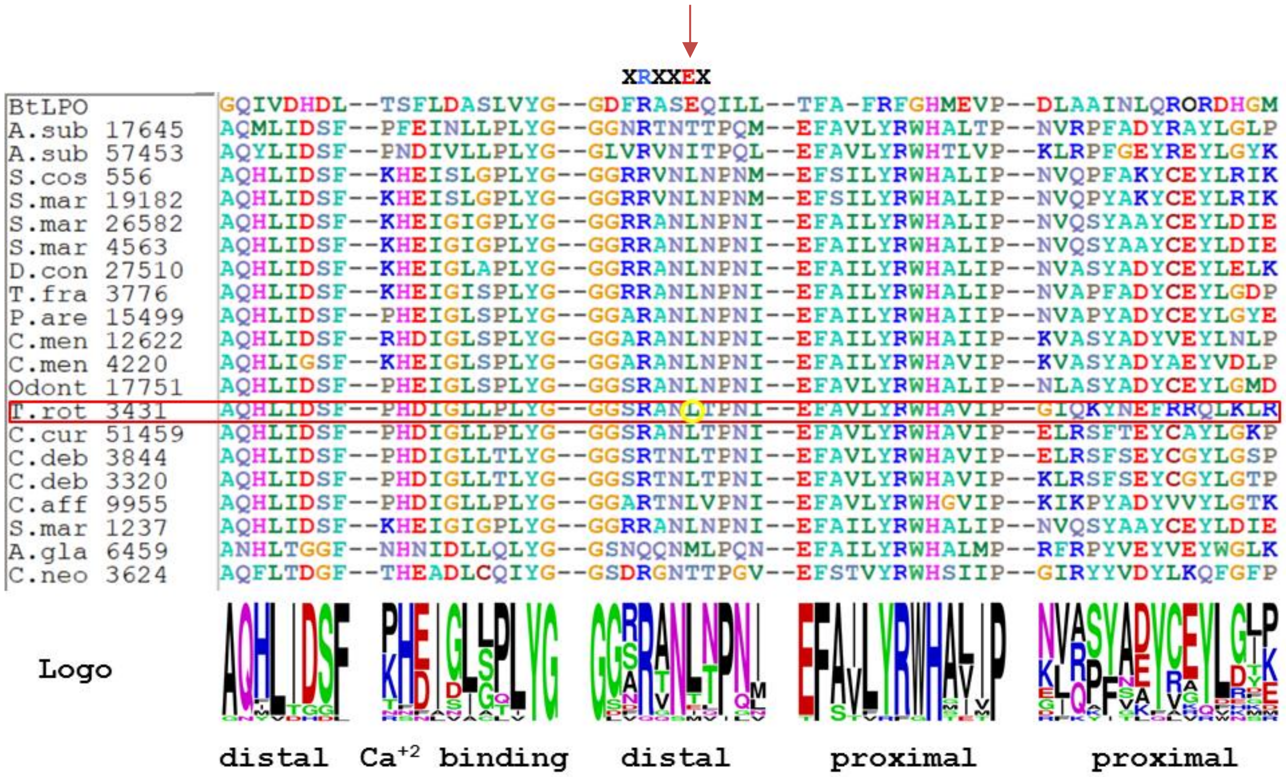

Supplementary table 1: List of species and sequences IDs used in the phylogenetic analysis.

**Species**	**Annotation**	**Sequence ID**
Chaetoceros neogracile		MMETSP0751-20121128_3624
Asterionellopsis glacialis		MMETSP0707-20130614_6459
Aulacoseira subarctica		MMETSP1064-20121228_17645
Aulacoseira subarctica		MMETSP1064-20121228_57453
Chaetoceros affine		MMETSP0090-20130426_9955
Chaetoceros curvisetus		MMETSP0717-20131115_51459
Chaetoceros debilis		MMETSP0149-20130528_3320
Chaetoceros debilis		MMETSP0150-20130528_3844
Odontella		MMETSP0015_2-20120614_17751
Cyclotella meneghiniana		MMETSP1057-20121228_4220
Cyclotella meneghiniana		MMETSP1057-20121228_12622
Pseudo-nitzschia arenysensis		MMETSP0329-20121206_15499
Thalassionema frauenfeldii		MMETSP0786-20121207_3776
Skeletonema marinoi		MMETSP0320-20121206_19182
Skeletonema costatum		MMETSP0013_2-20120614_556
Detonula confervacea		MMETSP1058-20130122_27510
Skeletonema marinoi		MMETSP1039-20121108_4563
Skeletonema marinoi		MMETSP0319-20121206_26582
Skeletonema marinoi		MMETSP0562-20121206_1237
Skeletonema marinoi		MMETSP1428-20130617_783
Synedropsis recta		MMETSP1176-20130426_10672
Nostoc sp.	COX-1	WP015113127
Chaetoceros cf. *neogracile*		MMETSP1336-20130426_1470
Grammatophora oceanica		MMETSP0009_2-20130614_5553
Gersemia fruticosa	COX-A	AAF93168
Gersemia fruticosa	COX-B	AAS48061
Plexaura homomalla	5S-specific-cyclooxygenase	AAU87497
Plexaura homomalla	COX-1	AAF93169
Gammarus sp.	COX-1	ADB65786
Caprella sp.	COX-1	ADB65785
Nitzschia sp		MMETSP0014_2-20120614_6887
Skeletonema marinoi		MMETSP0319-20121206_1511
Pseudo-nitzschia pungens		MMETSP1061-20121228_74475
Thalassiosira rotula		TR754|c0_g1_i1|m.3431
Skeletonema marinoi		MMETSP0920-20130426_33289
Fragilariopsis kerguelensis		MMETSP0907-20130614_13332
Skeletonema grethea		MMETSP0578-20130828_1168
Staurosira sp.		MMETSP1361-20130828_23824

Skeletonema marinoi		MMETSP1428-20130617_31509
Homo sapiens	COX-1	NP000953
Ovis aries	COX-1	NP001009476
Homo sapiens	COX-2	NP000954
Coccotylus truncatus	COX-1	AFN20596
Ciona intestinalis	COX-a	XP002127674
Ciona intestinalis	COX-b	XP002123273
Oncorhynchus mykiss	COX-1	CAC10360.1
Fistulifera solaris	Alpha-dioxygenase	GAX23950.1
Cyanothece sp.	Heme peroxidase	ACB53655.1
Fistulifera solaris		GAX18717.1
Alcanivorax nanhaiticus		WP_035233415.1
Hordeum vulgare subsp. *vulgare*		BAJ90503.1
Triticum aestivum		CDM84254.1
Rhizophagus clarus		GBC07128.1
Emiliania huxleyi		XP_005780718.1
Rhodococcus gordoniae		WP_064063209.1
Herbidospora mongoliensis		WP_066371138.1
Eutrema halophilum		BAJ34623.1
Pisum sativum	Alpha-dioxygenase	CAH05011.1

External standard calibration method for PG quantization.

For an external standard quantitation, known data from a calibration standard and unknown data from the sample are combined to generate a quantitative report. Working standard solutions containing all the analytes at 1 µg/mL were prepared, from stock solutions of 100 µg/mL for each standard: PGE_2, 15-d-PGJ_2, PGB_2, PGD_2, PGJ_2 (SIGMA ALDRICH, Saint Louis, MO), 20-oh-PGE_2, 6-keto-$PGF_1\alpha$, DHK (CAYMAN CHEMICAL, Michigan, USA). From these standard solutions, five solutions at different concentration levels were prepared: 1, 5, 25, 50, 100 ng/mL for the construction of the calibration curves. The coefficients of determination (R2) were used to evaluate the linearity of the calibration ranges. All R2 were greater than 0.99, demonstrating that the method was linear in the range of calibration for all analytes.

The amount of prostaglandins was normalized by the sampled volume extracted for each daily replicate and then by the number of cells per mL for each replicate. The final value reported in the graph were pg $cell^{-1}$.

Supplementary figure 5: calibration curve of deuterated prostaglandin E2 (PGE2-d4) used as internal standard.

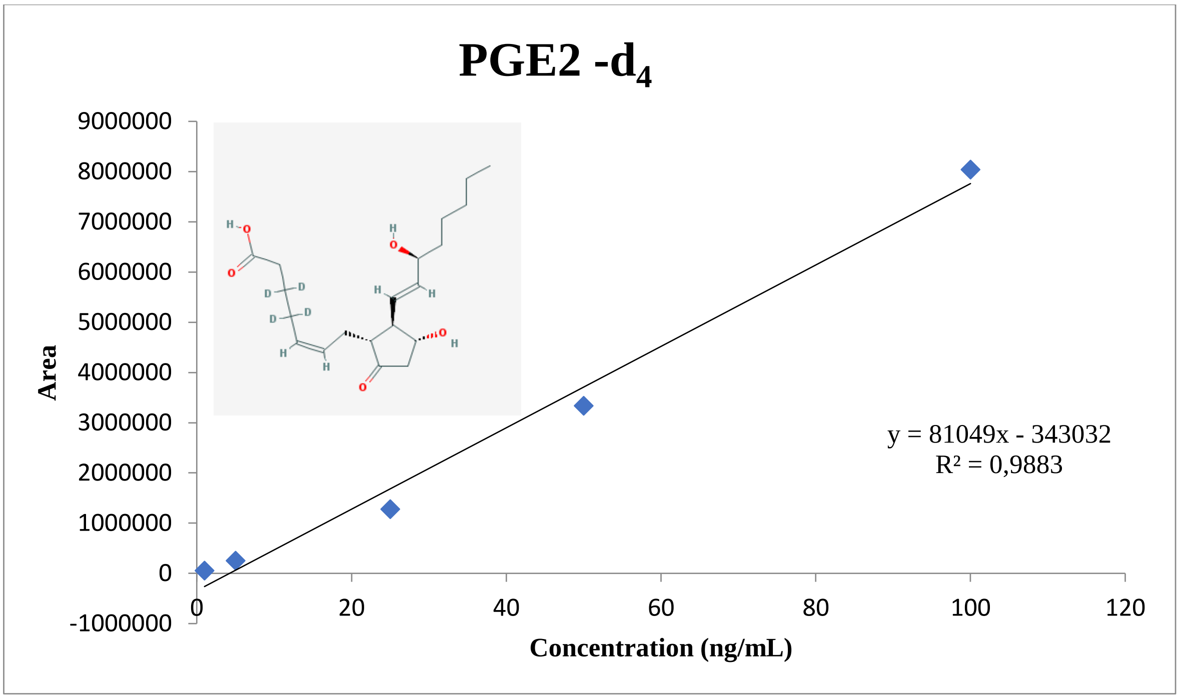

Supplementary figure 6: calibration curve of prostaglandin E2 (PGE2) used as standard.

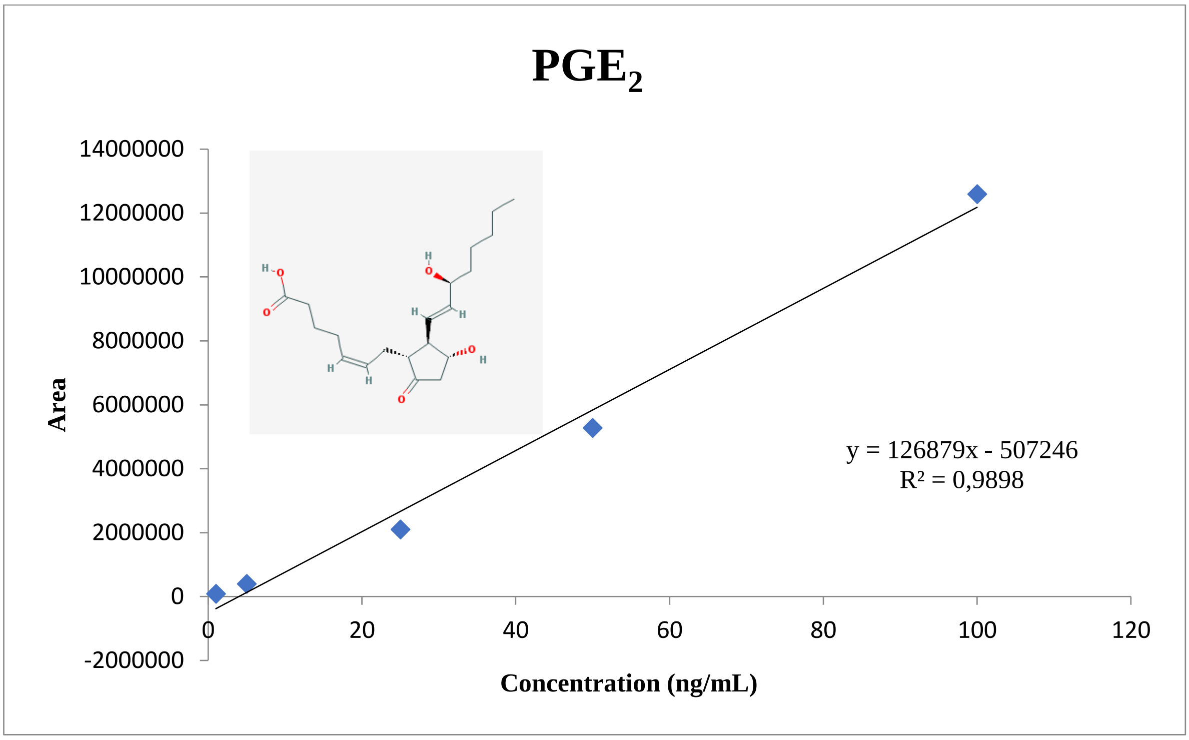

Supplementary figure 7: calibration curve of prostaglandin B2 (PGB2) used as standard.

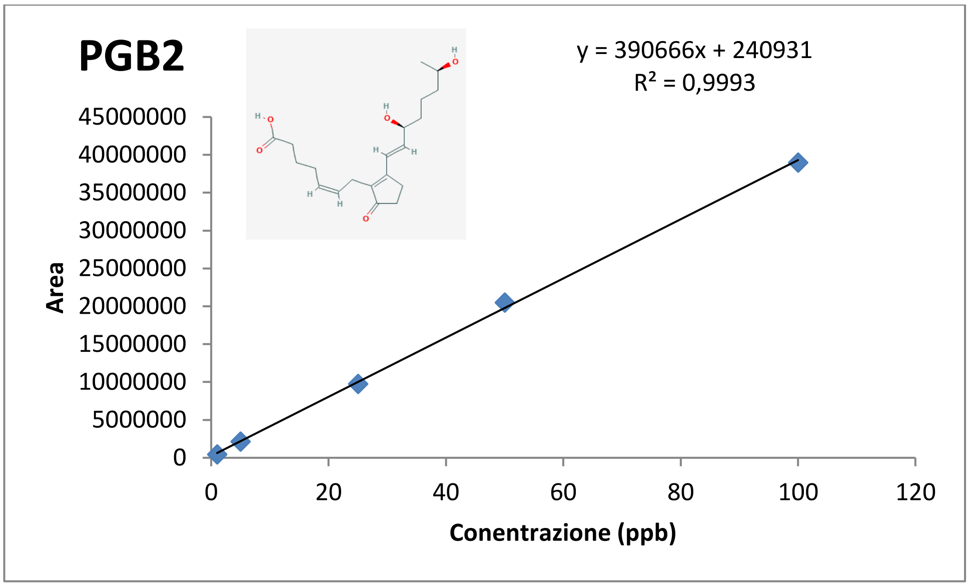

Supplementary figure 8: calibration curve of 15-Deoxy-Delta-12,14-prostaglandin J2 (15dPGJ2) used as standard.

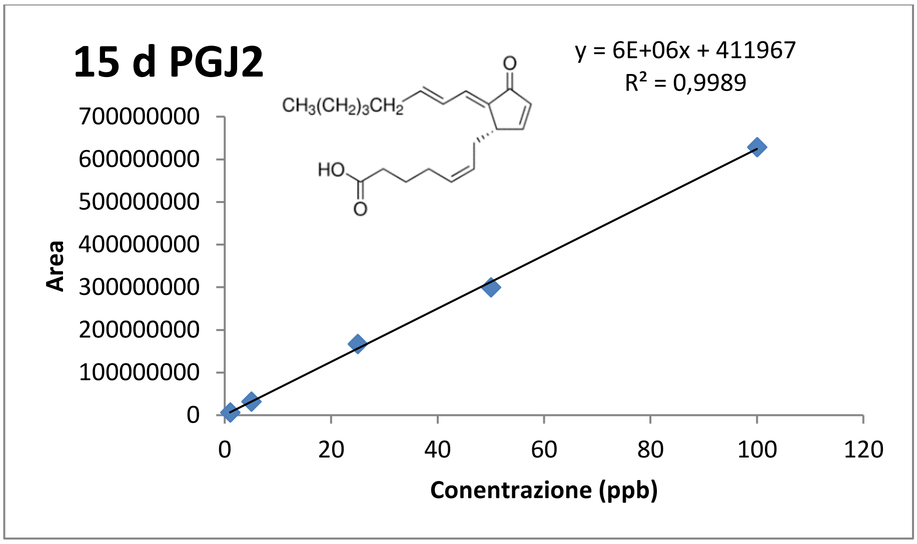

Supplementary figure 9: examples of TIC chromatograms.

a. a representative sample from the LC-MS/MS analysis of the culture medium and XIC chromatogram;

b. PGEM (327 m/z precursor ion; 291 m/z and 309 m/z daughter ion) peaks.

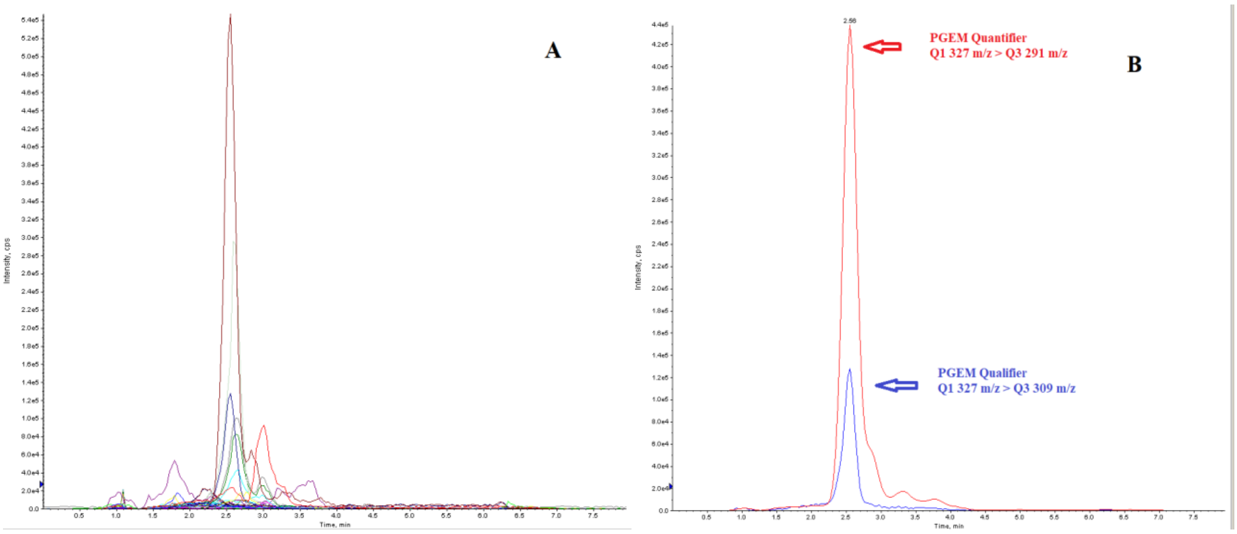

Supplementary table 3. List of PGs identified in the culture medium with their LC-MS/MS parameters.

Molecule ID	rt	Q1	Q3	DP	CE
**PGE_1**	1,13	353	235	-60	-29
**15-d-PGD_2**	2,14	333	271	-60	-22
**15-d-PGJ_2***	2,21	315	203	-60	-20
**PGE_3**	2,28	349	269	-60	-24
**PGE-M**	2,56	327	291	-60	-23
**PGA_2**	2,64	333	271	-60	-20
**PGB_2***	2,69	333	271	-60	-25
**PGF-M**	2,7	329	293	-60	-25
**2,3-dinor-11b-PGF_2**	3,05	325	227	-60	-22
**PGD_1**	3,14	353	271	-60	-29
**PGD_3**	3,14	349	269	-60	-20
**PGE_2***	3,23	351	272	-60	-23

ID: official abbreviated prostaglandin names; Q1: precursor ion (*m/z*) selected in quadrupole Q1; Q3: fragment ion (*m/z*) selected in quadrupole Q3; Q1>Q3: transition pairs utilized for quantification; DP: declustering potential; CE: collision energy for each molecule; rt: Liquid Chromatography retention time in minutes. Asterisks indicate prostaglandins identified by comparison with their own standard reference. The non-asterisk one indicates the prostaglandins identified by comparison with literature data

Aligned sequences, with gap, utilized in the phylogenetic analysis

Chaetoceros neogracile MMETSP0751-20121128_3624
MVFPNFAQFLTDGFAK-----SIPG------------
TNFLRNNATHEADLCQIYGRNKDQTDCLRLMS-SSIGEKGRLKSCMING-----
EEWPMPYFLESGEVDP---------------------------QFERLDPPKFSFDHVIEQLSRIDPNGRLGLVQ-
-----------------------KIKKNIFAVGSDRGNTTPGVAAFASLFLREHNRLAAEVENR---------NPA--
----WDDGQIFQTARNINIVIYIRIIMEDYLNQLSHAP--
-----FDFKLDPGPWTWDAKWNKK------FRVAVEFSTVYR-WHSIIPNAIQIGE---------------
GDPLPIMQAMFNNELLIDR------TLLKSFEDSSSQRATSFTPFNTAD--------------------
IMLPREYNTILQARQAGIRYYVDYLKQFGFPVDLPKNYSDITS-DEKVQ----------------
KMLEEMYGKG--NVDKVEFYIGAICATHERN-A----
PFSLFMNLQVAHDAIKAFYMNPLLQRSSWK----

Asterionellopsis glacialis MMETSP0707-20130614_6459
MILPTFANHLTGGFIK-----STRG--PDGS------
YQYGRTHSNHNIDLLQLYGRTQEQTLALRLRDNTTFGKKGRLKSQILNG----
GEEWPPFLYKDGRIDPQ----------------------------FEVLDPIAHGMSHLVDIMEGD------------------
------------------RSKFFAMGSNQQNMLPQNIAMTTLFLREHNRVAGELERR---------YSD------
WDDDRIFETARNIVIVIYLKIVIEEYINHISPLP---
DIVKFLVDPGPWMWNAEWNKP------NWISAEFAILYR-WHALMPNKVMHA----------------
GIVFDLSSTLFQHDLLLGED----RSLKKTFVDMSAQRSASCECLNTAG--------------------
PLVEREVESLRYSRALRFRPYVEYVEYWGLK--KPRRFEDITK-NAEVA----------------
SMLRDLYGT----VDKVEFFVGLIASDHSKN-G----
IFGSSMNIGVGLDAFSQALTNPLLSEHVWK----

Aulacoseira subarctica MMETSP1064-20121228_17645
MLFPVFAQMLIDSFIS-----TVTYTNTTTK---
KLTFDWKRTNSPFEINLLPLYGRFEYQTDALRLKS-EKS--RGRLKSQIING-----
EEYAEFLYNDLGEIKD---------------------------EFQVLGPPQA-FNQILSKVDKTEEEKR-----------
------------------KIKSKIFAFGGNRTNTTPQMAALNTLFLREHNRLAGQLEKH---------NED------
WDDDRVFQTARNINLVIYLKIIIEEYINHITSS---
GARFKVQPKEWVWNADWNKP------NWIAVEFAVLYR-WHALTPNSYLWE----------------
GKRVKISDDLFNNALLLET----SGGLRQAIAEISKNPATIMAPFNTAL--------------------
ELLSQEQAAFAQTRQANVRPFADYRAYLGLP--LVKTFKDITQ-DKEVQ----------------
QKLEELYKT----PDRVEFWAGLVAEDKDPK-A----
IFGPTLSTLVALDAFSQALTHPLLSEQVFN----

Aulacoseira subarctica MMETSP1064-20121228_57453
MLFPVFAQYLIDSFIL-----TKRIKTEGGT---VSTIDWKRSDSPNDIVLLPLYG-
FKNATDALRLKS-EEAQKRGKLKSQLIDG-----EEYAPFLYDESGMVKE---------------------------
EFKALG-EPESLGEILKAMMADSNTVK-----------------------------
KYKSKIFAFGLVRVNITPQLAAMNTLFLREHNRLAGVLEEN---------NPE------
WDDERVFQTARNINLVIYLKIVIEEYINHISS--
GVQFKAKPEKWIWHASWNKP------NWISVEFAVLYR-WHTLVPNSDEWN----------------
GIRYDFQNEIFNNEILLD-----AGGLRHSFASISANRAPSICPFNTGG--------------------
FLLPREASALQQSRVNKLRPFGEYREYLGYK--KTKEFSDISS-DPQVQ----------------

ETLRKLYKT----PDRVEFYAGLIAEDHVAK-G----
ILAPIMTDLVAKDAFTQALTHPLLSENVFN----

Chaetoceros affine MMETSP0090-20130426_9955
MLFPTFAQHLIDSFIV-----TAVKSDGGSG----
TEFEWKKTDSPHDIGLLPLYGRTFDQTKQLRVQN-PPRGKYGQLKSQIIHG-----
EEYAPYLYDADGKVKK---------------------------EFDLLE-TPQGLERSLSMLSPE--DAKA---------
-------------------K-KSNIFAFGGARTNLVPNITAWNTLLLREHNRIAQTIEKE---------EPT------
WDDERVFQTARNVLLVIYLKLVVEEYINHITGY--
---GIDFTVDPGKWMWNAPWYKR------NWISAEFAVLYR-WHGVIPSCMKWG----------------
DKTLSTHESLFNNAVLTED---MKGSLRDTFINISNHRATQMNLFNTES--------------------
MMVLRDMAALKQCRACKIKPYADYVVYLGTKE-RPTKFSDISK-DKEVQ----------------
EALEKVYKK----VENVEFWTGLLASDNPPE-G----
IMSPEMTTFVANDAFNQALCHPLLSENVWS----

Chaetoceros curvisetus MMETSP0717-20131115_51459
MLFPTFAQHLIDSFIV-----TAIKENSSEG----
VVFNWRKTGSPHDIGLLPLYGKTIEQTTQLRLKS-EVKGSKGRMKTQIIKG-----
GEWSPYLFDSKGNKKA---------------------------EFSSLP-DPEGLEFALMEAKKFGVDAEK-------
---------------------N-KASVFALGGSRANLTPNIVAWNTLLLREHNSIAAKIEAE---------NPE------
WDDERVFQTARNVNLAVYLRLVIEEYINHITAF---
--GVDFTVEPGKWMWDAPWYKR------NWISAEFAVLYR-WHAVIPNCMKWG----------------
EKTLPTAAYLYSNHLLLDDT-GLKGDLREAFINISNHRATSMEIHNSEK--------------------
WMTGRDSRALQMSRECELRSFTEYCAYLGKP--VPKTFADITS-DVEVQ----------------
NELKALYGK----VENVEFWVGLIAKDHPTE-A----
IMSAELTTFVANDAFNQALTHPLLSEHVWP----

Chaetoceros debilis MMETSP0149-20130528_3320
MLFPTFAQHLIDSFID-----TVYHYDDDGN----
VVFDWKRTETPHDIGLLTLYGKTIPETKQLRKQS-ETSGEKGKLKSQLVNG-----
EEWAPFLYDSNGKVKE---------------------------EFNELP-VPQGIDEKMYAARPQ-VQAKL-------
---------------------K-ESIFAFFGGSRTNLTPNISAWNTLLLREHNRIAGLIEEE---------NPT------
WDDERVFQTARNCTLVIYLRLVIEEYINHITAY---
-GVDFEIEPEKWMWDAPWYKR------NWISAEFAVLYR-WHAVIPSLMKWG----------------
KNTHTTMDYLFSNNLLLSDD-GMKGNLRDCFHNICDHRATNMQLHNSEGG-------------------
FMVGRDKSALEMSRSCKLRSFSEYCGYLGTP--APESFADITQ-DKDLQ----------------
KELKDVYGE----VKNVEFWTGLIAKDHSCE-A----
IMSAELTKFVANDAFNQALTHPLLSEHVFN----

Chaetoceros debilis MMETSP0150-20130528_3844
MLFPTFAQHLIDSFID-----TVYHYENDGN----
IVFDRKLTGTPHDIGLLTLYGKTIPETKQLRKQS-DTSGEKEKICPNLLKE-----
KSXAPFLYDSNGKVKE---------------------------EFNKLP-VPQGIDGKMYAARPQ-VRAKL-------
---------------------K-ESIFAFFGGSRTNLTPNISAWNTLLLREHNRIAGLIEED---------NPT------
WDDERVFQTARNCTLVIYLRLVIEEYINHITTY---
-GVDFKIEPEKWMWDAPWYKR------NWISAEFAVLYR-WHAVIPSLMKWG----------------
KNTHTTMEYLFSNNLLLSDD-GMKGNLRDCFHNICDHRATNMQLHNSEGG-------------------
FMVGRDKSALEMSRSCELRSFSEYCGYLGSP--APESFADITQ-DKDLQ----------------

KELKDVYGE----VKNVEFWTGLIAKDHSCE-A----IMSAELTKFVANDAFNXGFD----
SSSPFR----

Odontella MMETSP0015_2-20120614_17751
LLFPTVAQHLIDSFIN-----TKYTKTING-----
TFFDWARTDSPHEIGLSPLYGCNPKQTSQLRELN-EKIGCKGRLKTQILGD---
GGEEWAPFLYGDSGKIKA--------------------------EFNELD-EPVALDHILGFNSENAEMMR-----
--------------------------KSIFAFGGSRANLNPNIVAWNTLLLREHNRVASKIEAS---------EPS------
WDDERVFQTARNVVLVIYLKIVIEDYVAHIS--
GVNFKVQPGEWMWNAKWYKR------NWMSVEFAVLYR-WHALIPNTTFWG---------------
SKTASTKDALYNNTLLLNKEQGCAGNLRNIMVQISEQRITSFQLNNTED-------------------
WLVDRDLNALRQSRECNLASYADYCEYLGMD--RPKTFTDISL-YPEVQ---------------
EKLRSLYKT----ADKVEFYVGLIAADHSPGGK----
LFSEAMTRFVANDAFNQALANPLLSENVWK----

Cyclotella meneghiniana MMETSP1057-20121228_4220
LLFPTFAQHLIDSFIN-----
TRIDHEATEKNGGRPVFDWARTDSKHEIGLSPLYGDTEEQLNQLRLLS-
NVNGFKGLMKTQIINE-----EEWAPFLYEVDGSKKK--------------------------EFSAIW-
DPSGASYVLGMRRDVGERQK-------------------------------
RTLFAFGGARANLNPNIVAWNTLLLREHNRIAGEIEKS---------EPS------
WDDERVFQTARNVLVVIYLKLVIEEYIAHIA--
GVNFKVDPGEWMWNAPWAKT------NWMSTEFAILYR-WHAVIPNTSSWGK---------------
AKNLKVLDTLFNNDLLLDTKEGLSGNLRDAFVSISEERVTAHQLFNTEE-------------------
WMVDRELAAIKQGRANKVASYADYAEYVDLP--RPKTFADISL-YPEVQ---------------
KALEEVYGT----VDRVEFYVGLIAVDMGAGGK----IFSP-
MTKFVANDAFNQALTNPLLSMNVWN----

Cyclotella meneghiniana MMETSP1057-20121228_12622
LLFPTFAQHLIGSFIN-----
TKIDHEATEKNGGQVIFDWAKTYSRHDIGLSPLYGDTTEQTDQLRLMS-
NVTGFKGRMKTQLIND-----EEWAPFLYRVDGTKKP--------------------------EFNAIW-
DPAGASHVLKMRGEEGFKQK-------------------------------
RTLFAFGGARANLNPNIVAWNTLLLREHNRLAGEIEKS---------EPS------
WDDERVFQTARNVNIVIYLKLVIEEYIAHIS--
GADFKVDPGEWMWNADWNKA------NWMSVEFAILYR-WHAIIPNTINWG----------------
TSNMKVSDILFNNDLLVKETEGLDANLRDVFVQMSEQRATAHQLFNTEE-------------------
WLLDRELAAVTQGRANKVASYADYVEYLNLP--RPKTFADISL-YPEVQ---------------
NALKEVYGT----VARVELYVGLIAADMGAGGK----IFSL-
MTKFVANDAFNQALTNPLLSQNVWK----

Pseudo-nitzschia arenysensis MMETSP0329-20121206_15499
LLFPTVAQHLIDSFIN-----THVDRKATEAKG-
SPVFNWAKTDSPHEIGLSPLYGDSKEQTDQLRERS-EEVGRKGRLKSQMIEG-----
EEWSPFLYDSKGNKKT--------------------------EFSKIP-DPSGMSHILKHVNAATAKKA----------
----------------------SIFAFGGARANLNPNIVAWNVLLLREHNRLASMIEES---------EPT------
WDDERVFQTARNVSIVIYLKIVIEEYIKHIS--
GANFRVAPGKWLWNAQWNKT------NWMSVEFAILYR-WHAIIPNSSSWGP--------------

SKDVEVRESLFNNTLLLDKTKGMGAKLADIFVQISNERTTSFELNNTEK--------------------
WLVDREMAAIKQGRTNNVAPYADYCEYLGYE--RPKTFADINR-DPKVQ----------------
EQLKELYGT----PDKVEFYVGLIAGEHPSGGK----IFSK-
MTSFVANDAFNQALGNPLLSQNVWE----

Thalassionema frauenfeldii MMETSP0786-20121207_3776
LLFPTFAQHLIDSFIN-----TRIKADTDKNK--
PPEFEWTRTDSKHEIGISPLYGDEPAQTKQLRELS-ETSGRKGRLKTQMIEG-----
EEWAPFLYNGN-NKKP---------------------------EFSDIP-EPDGVRMIEKHG---FGDRS--------------
-------------------TIFAFGGRRANLNPNIVAWNTLLLREHNRLAAEIEKS---------EPE------
WDDERVFQTARNVLTVIYSKIVVEEYVGHIS--
GVPFEVQPGPWMWNAEWNKT------NWMSVEFAILYR-WHALIPDTIRWGP---------------
KVDIGIMKQLFNNTLLLSKENGMGANLKDCFTEISRNRVTSFELFNTEGS-------------------
YMATREMQAIRQCRAANVAPFADYCEYLGDP--RPKTFEDISR-KPEVQ----------------
KVLKELYGT----PDRVEFYVGLIAQDHSAGPK----
IFGDVMTKFVANDAFNQALANPLLSQNVWE----

Skeletonema marinoi MMETSP0320-20121206_19182
LLFPTFAQHLIDSFIN-----TKLN-TETG------
QFEWDQTESKHEISLGPLYGDDVGQTNQLREKS-EVTGRRGRLKTQILDG----
GEEWAHFLYDETGTKKE---------------------------EFNLIR-DPDGMKHILKALYNSDPAAKS-----
-----------------------SIMQTIFAFGGRRVNLNPNMVAWNTLLLREHNRLAGEIERS---------EPS---
---WDDERVFQTARNVLIVIYLKLVIQEYIGHIS---
--GVKFKLDPGEWMWNAPWYKT------NWMSTEFSILYR-WHALIPNQNGLGP---------------
SKDLGVLKSLFNNPLLLDEEKGLGGNLRDVFVDICKARITSFQLFNTEK--------------------
WMVGREAATISQGRANNVQPYAKYCEYLRIK--PPKTFKDISL-VPEVQ----------------
QALEELYGT----PDRVEFYVGLIAADHAPG-K----IFSL-
MTKFVANDAFNQALTNPLLSQNVWE----

Skeletonema costatum MMETSP0013_2-20120614_556
LLFPTFAQHLIDSFIN-----TKLN-TETG------
KFEWDQTESKHEISLGPLYGDDVGQTNQLREKS-EVTGRRGRLKTQILDG----
GEEWAHFLYDETGTKKE---------------------------EFNLIR-DPDGMKHILKALYSSDPAAKS-----
-----------------------SIMQTIFAFGGRRVNLNPNMVAWNTLLLREHNRLAGEIERS---------EPS---
---WDDERVFQTARNVLIVIYLKLVIQEYIGHIS---
--GVKFKLDPGEWMWNAPWYKT------NWMSTEFSILYR-WHALIPNQNGLGP---------------
SKDLGVLKSLFNNPLLLDKEKGLGGNLRDVFVDICKARITSFQLFNTEK--------------------
WMVGREAATINQGRANNVQPFAKYCEYLRIK--PPKTFKDISL-VPEVQ----------------
QALEELYGT----PDRVEFYVGLIAADHAPG-K----IFSL-
MTKFVANDAFNQALTNPLLSQNVWE----

Detonula confervacea MMETSP1058-20130122_27510
LLFPTFAQHLIDSFIN-----TKTKPLESGRL----
GFEWDQTFSKHEIGLAPLYGDYEEETLQLREKS-EASGRKGRLKTQVLDG----
GEEWAPFLYDSEGTKKP---------------------------EFSLLP-DPDGLQHIL----AMQPETME----------
------------------KKKKSIFAFGGRRANLNPNIVAWNTLLLREHNRLASEIEKS---------EPS------
WDDERIFQTSRNVVIVMYCKIIIEEYIKHIS--
GVNLRVEPGEWMWNASWYKP------NWISTEFAILYR-WHALIPNTSSWGA---------------

SKNIEVVDALFNNNLLISKDTGMGGNLRDAFVEISKTRITSFELFNTEK-------------------
AMVQRETAAIEQGRFNNVASYADYCEYLELK--RPETFEDISL-KPEVQ---------------
QALKELYGS----PDRVEFYIGLIAADHPAGGK----
VFSAAMTKFVANDAFNQALTNPLLSQNAWN----

Skeletonema marinoi MMETSP1039-20121108_4563
LLFPTFAQHLIDSFIN-----TKIN-VETG------EFEWDRTDSKHEIGIGPLYGDEVEQTSQLREKS-
EKPGRRGRLKTQVLEG----GEEWAPFLYNEDGTKKK-------------------------EFSAIH-
DPDGMKTILGLVYSSDPTTKS--------------------------
SIEQSIFAFGGRRANLNPNIVAWNTLLLREHNRLAGEIEKS---------EPG------
WDDERVFQTARNVLIVMYCKIVIEEYIKHIS--
GVNFKVEPGPWMWNAPWYKT------NWMSTEFAILYR-WHALIPNEAGLGP--------------
SKDAGVMEALFNNPMLLDDETGLGGNLRDIFVDISQTRVTSLQLFNTEK-------------------
WMVERESAAINQGRANNVQSYAAYCEYLDIE--PPKTFEXISM-VPERQ---------------
QALKELYGT----PDRVEFYVGLIAADHPAGGK----IFSE-
MTKFVANDAFNQALTNPLLSQNVWE----

Skeletonema marinoi MMETSP0319-20121206_26582
LLFPTFAQHLIDSFIN-----TKIN-VETG------EFEWDRTDSKHEIGIGPLYGDEVEQTSQLREKS-
EKPGRRGRLKTQVLEG----GEEWAPFLYNEDGTKKKE--------------FSAIEDGTKKEEFSAIH-
DPDGMKTILGLVYSSDPTTKS--------------------------
SIEQSIFAFGGRRANLNPNIVAWNTLLLREHNRLAGEIEKS---------EPG------
WDDERVFQTARNVLIVMYCKIVIEEYIKHIS--
GVNFKVEPGPWMWNAPWYKT------NWMSTEFAILYR-WHALIPNEAGLGP--------------
SKDAGVMEALFNNPMLLDDETGLGGNLRDIFVDISQTRVTSLQLFNTEK-------------------
WMVERESAAINQGRANNVQSYAAYCEYLDIE--PPKTFEDISM-VPERQ---------------
QALKELYGT----PDRVEFYVGLIAADHPAGGK----IFSE-
MTKFVANDAFNQALTNPLLSQNVWE----

Skeletonema marinoi MMETSP0562-20121206_1237
LLFPTFAQHLIDSFIN-----TKIN-AETG------EFEWDRTDSKHEIGIGPLYGDEVEQTSQLREKS-
EKPGRRGRLKTQVREG----GEEWAPFLYNKDGTKKE-------------------------EFSAIH-
DPDGMKTILGLVYSSDPTTKS--------------------------
SIEQSIFAFGGRRANLNPNIVAWNTLLLREHNRLAGEIEKS---------EPG------
WDDERVFQTARNVLIVMYCKIVIEEYIKHIS--
GVNFKVEPGPWMWNAPWYKT------NWMSTEFAILYR-WHALIPNEAGLGP--------------
SKDAGVMEALFNNPMLLDDETGLGGNLRDIFVDISQTRVTSFQLFNTEK-------------------
WMVYRESAAINQGRANNVQSYAAYCEYLDIE--PPKTFEDITM-VPERQ---------------
QALKELYGT----PDRVEFYVGLIAADHPAGGK----
IFSEAMTKFVANDAFNQALTNPLLSQNVWE----

Skeletonema marinoi MMETSP1428-20130617_783
LLFPTFAQHLIDSFIN-----TKIN-AKTG------
EFEWDKNDSKHEIGIGPLYGDEVEQTNQLREKS-EAPGRRGRLKTQVL-E---
GGEEWAPFLYNEDGTKKK-------------------------EFSAIH-DPDGMKTILGLVYSSDPTTKS----
----------------------SIEQSIFAFGGRRANLNPNIVAWNTLLLREHNRLAGEIEKS---------EPG----
--WDDERVFQTARNVLIVMYCKIVIEEYIKHIS--
-GVNFKVDPGPWMWNAPWYKT------NWMSTEFAILYR-WHALIPNEAGLGP--------------

SKDAGVMEALFNNPMLLDDKTGLGGNLRDIFVDISQTRVTSFQLFNTEK--------------------
WMVYRESAAINQGRANNVQSYAAYCEYLDIE--PPKTFEDITM-VPERQ----------------
QALKELYGT----PDRVEFYVGLIAADHPAGGK----
IFSEAMTKFVANDAFNQALTNPLLSQNVWE----

Synedropsis recta MMETSP1176-20130426_10672
PTKPWLGQGCRGMALR---
FPKRPLPDNLPDAKTLVEDFCIRPDDDASFTPCANGVNSLTPYFALCVIHDFFRSDTGRSKV
GKLDR---PWVNLHSSYLDLQTVYGYN----------------------------------KKSCASVRTQKHG------
------------------------------KLTEKKIVDHRLERMAVCHALILVMVKHHNFICDQLMER---------
YPD----KFKTDEMIFQTARLINCGVYINLIIELYSCLFHVWN--
--------------EDGSNPVELRGLDYPRDIQGY-------HLSYEFNIMYR-FHAFIPKEWTPFYG---------
-------LKEAIAYLKKGDFTHSVPALSDDAIKTLLVDAMSHRAGANHVPSNVPR------------------
-VMGPAEVKGIEDARLLGISTYNDFKEAVGEP--KYDTFLDMSGNRPELA----------------
AKLEKHYPT----VDDVEFAVGMRVETRTPLRG----
AGFSTVGRAILADAFSSIRFDRFYSQSEYH----

Nostoc sp WP015113127
MLFPYWVQWFTDSFLR-----LDHTN-------------
KLKNTSNHEIDLCNVYGLTRKQTHLLRSFQ------
GGKFKTQKLKRQDGVEEEYPLFYYADPAQG-----------------------------IVDPQFAGLYE-
PVNDE---KRQPAD-------------------------------
KKQYLFAMGVERANVQIGYVMLNTLCLREHNRLCDVLASN---------YPD------
WDDERLFQTSRNILMAIILKIIMEEYINHITPY---
HFKLFADPEAFTK-ESWYRT------NHMAIEFDFVYR-WHSAIPETFNCNG----------------
KPTHVADTLWNNKILIDQG------LGALMEETCSQAGTRIGLFNTPNI-------------------
LVDLAELPSIKLGRQLQLASYNDYRELCGFP--RVTSFDQITG-DEFTQ----------------
QKLKEFYGH----VDNIEFFVGLYSEDVRQN------STIPPVARLIGIDAFSQALTNPLLSPKIFN---
-
Chaetoceros cf neogracile MMETSP1336-20130426_1470
LMLPIFAQWFTDSFLR-----TKFRVDGPQD--------
FKENESNHEIDLCQIYGMTETQTAMLRSME------NGFLKSQIIDK-----EEFPAHLFEETASG---
LV------------------------QIKPEFAGLYT-EANVQRVFRRASKE-------------------------------
HKLNCFAVGIEHGNSTMGNTLMNIIWMREHNRIAREIAKA---------HPT------
WDDERLFQTARNVNIVLLLKVVVEDYIFHIS--
GFPFKMDN-KIAEGERWYRS------NWMAAEFALLYR-WHDLIPDTVEFGG----------------
ERKDSSALNKNNRWLMKVG------VDRACLDASQEPSGKIMLGNTPNF-------------------
LVEVGKMS-LKQGRVSGLASFNDYCEHYGLK--RKKNFMDLTGNNKEVS----------------
KKLEDVYGS----IDNLEWFVGLWAEAYDDKTK----CMGP-
LTYMVGNDAFTQALTNPLLAAEVFN----

Grammatophora oceanica MMETSP0009_2-20130614_5553
LLLPMFAQWFTDGFLR-----TKWKPPTEQD--------
YVENESTHQIDLNQIYGATEVQTNILRAKK------GGKLRSQMIKG-----EEYPEYLFDTET------
L------------------------QIKPEFEGLYS-DVNFTRVFQNVSDE-------------------------------
QKKVTFAVGLEHGNSTVANTIMNTLFLREHNRLAGIIEAA---------HKD------
WDDERIFQTTRNCLIVILMKIVINDYVAHIA--
DAPVFLDAGGFAEDQPWYRE------NWMSVEFNLLYR-WHDMIPDQVSFTSADQVSFS---

SEDEYWDMESSKTLVNNNTLMLELG------VEQILLDASKQRAGQLGLFNTPDF----------------
---LLHVHKET-MKQCRMINLAPYVDYCKEYGLT--VPKTFRELTGGNLEYA----------------
KALESVYDS----VEHVEWFVGLWAGG-KALGQ----
FSSDLLILMVGHDAVTQLFTNPLLSRRVYN----

Gersemia fruticosa AAF93168
WLFMFFAQHFTHEFFK-----TIYHS-PAFT-----------WGN-
HGVDVSHIYGQDMERQNKLRSFE------DGKLKSQTING-----EEWPPYLKDVDNVTMQY-----
----------------------------PPNT--
PEDQKFALGHPFYSMLPGLFMYASIWLREHNRVCTILRKE---------HPH------
WVDERLYQTGKLIITGELIKIVIEDYVNHLAN--
YNLKLTYNP-ELVFDHGYDYD------NRIHVEFNHMYH-WHPFSPDEYNISG----------------
STYSIQDFMYHPEIVVKHG------MSSFVDSMSKGLCGQMS-HHNHGA-------------------
YTLDVAVEVIKHQRELRMQSFNNYRKHFALE--PYKSFEELTG-DPKMS----------------
AELQEVYGD----VNAVDLYVGFFLEKGLTT-S----
PFGITMIAFGAPYSLRGLLSNPVSSPTYWK----

Gersemia fruticosa AAS48061
WLFMFFAQHFTHEFFK-----TIYHS-PAFT-----------WGN-HGVDVSHIYGQDIERQNKLRSFQ--
----DGKLKSQMING-----EEWPPYLKDVDNVTMQY---------------------------------PPNT------------
--------------------------------SEDQKFALGHPFYGMLPGLFMYASIWLREHNRVCTILRKE--------
-HPH------WEDERLYHTGKLIITGELIKIVIEDYVNHLAN--
------------YNLKITYDP-ELVFDHGYDYD------NRIHVEFNHMYH-WHPFSPDEYNISG-----------
-----STYSIHEFMYHPEIVVKHG------MSSFVDSMSKGLCGQMS-HHNHGA-------------------
YTLDVAVEVIKHQRELRMQSFNNYREHFALE--PYKSFEEMTG-DPKMA----------------
AELQETYGD----VNAVDLYVGFFLEKGLTT-S----
PFGITMIAFGAPYSLRGLLSNPVSSPTYWK----

Plexaura homomalla AAU87497
WLFMFFAQHFTHQFFK-----TVHHS-PAFS-----------WGN-
HGVDVSHIYGQGVERENKLRAFK------DGKLKSQMING-----EEYPPYLKDVDDLKMQY-----
----------------------------LENT--
AEEQKFALGHPFFSMLPGLFMYATIWLREHNRVCMILRKE---------HPH------
WEDERIYQTGKLIITGELIKIVIEDYVNHLAN--
YNMKLRYDP-QLVFSRNYDYD------NRIHLEFNHLYH-WHPFSPDQFNISG----------------
TTYAIKDFMYHPEIVVKHG------MSSFVNAMSSGLCGKMS-HHNHGQ-------------------
YTLDVAVEVIKYQRELRMQSFNXYRRHFGLH--AYKSFEEMTG-NPKMA----------------
AELKEVYGD----VNAVDFYVGFFLEKSLTT-S----
PFGITMIASGAPYSLRGLLSNPVSSPTYWK----

Plexaura homomalla AAF93169
WLFMFFAQHFTHQFFK-----TVHHS-PAFS-----------WGN-
HGVDVSHIYGQGVERENKLRAFK------DGKLKSQMING-----EEYPPYLKDVDGLKMQY-----
----------------------------LENT--
AEEQKFALGHPFFSMLPGLFMFATLWLREHNRVCMILRKE---------HPH------
WEDERIYQTAKLIITGETIKIVIEDYINHLAN--
YNMKLRYDP-QLVFSRNYDYD------NRIHLEFNHLYH-WHPFSPDQFNISG----------------
TTYTINDFMYHPEIVVKHG------MSSFVNAMSSGLCGKMS-HHNHGQ------------------

YTLDVAVEVIKYQRKLRMQSFNNYRRHFGLP--AYKSFEEMTG-DPKLA----------------
AELKEVYGD----VNAVDFYVGFFLEKSLPT-S----
PFGITMIASGAPYSLRGLLSNPVSSPTYWK----

Gammarus sp ADB65786
LLFQYYAQHFTHQFFR-----TNYTKGPQFT-----------KGN-
GGVDVSNIYGLTERQRRALRSNV------DGKLKFQIING-----EHFPPYLKDVPGISMEY----------
-----------------------PPHLP---
ITEDNKFALGHPFFALLPGLFVYSTIWMREHNRVCEVLKEQ---------HPH------
WDDERLYHTAKLIITGEVIKITIEDYVQHLSQ---
YKVDLKFKP-QVVHGTRFQFH------NRINVEFDHLYH-WHPLIPEGIKVED----------------
SYYSLMDMAFSTKSVFTHG------LDAFVKALVTNRAGKLT-SRNHSP-------------------
VTVPVLKKMLENSRILRFQGVNQYRKKFNMR--PFRDFLDLTG-DEELA----------------
RDMEEMYGD----INAVEYYVGLIAEKDSP--S----
LTPLTMVNVGGPWSVKGLIANPICSPHWWK----

Caprella sp ADB65785
LLFQYYAQHFTHQFFR-----TNYTMCPQFT-----------KGN-
GGVDVSNIYGLTEQHRRAIRMNS------DGKLKYQVIND-----EHYPPYLRDVQGIEMDY-------
--------------------------PPHIP--
ITEDNKFALGHPFFALLPGLFVFSTIWMREHNRVCDVLKNQ---------HPD------
WDDERLYQTAKLIITGEVIKITIEDYVQHLSQ---
YKVDLKFKP-QVVHGTRFQFD------NRINAEFNHLYH-WHPLIPDGIQVED----------------
KYYSLMDMAFSTKSVFTHG------LDKFIESMATSRAGKLS-HSNHPL-------------------
VTLPVLKKMMENGRKLRYQGINEYRKRFALK--PFKDFMDLTG-DEALA----------------
KDLQELYGH----VDAVEFYVGLLTEKDSP--S----
LTPLTMVNVGGPWSVKGLIANPICSPHWWK----

Nitzschia sp MMETSP0014_2-20120614_6887
PQLNFWMLSFVNWFHD-----DNFRTLPDTDG-------
AFTWSDRGSLHMTHLYGHTEYRQAALRTMA-----GDGKMKTSSRLG----
WDYYPPLLMDVQADFPD-------------------------FDMWTSQRGSKHKSTSAGQTSEQADEN----
---------------------------MPYYFAIGDPRFNLHLGHILWTSVGLYLHNTACVILQRE---------DPG-
-----LTDEDIFQRARVIVFHIIQKIRLQDFVMDSISSTR---
----DHIRIPYDPKLLREEFAHHFAYSGGN-QPNFLEFNHLYQAWHALIPNGLVLNEDVD-------
--DGEKDILPIRKTLWAPKLMTTNFT-----IGEMATSFASTPLTLYSPHNFPVF--------------------
LRGVTEAALKDERAQRMAPYNSYRELIGLD--PITSFEQFAVDDP-------------------
QKMAELYNN---DVDSVDFIAGILADSNPHLPG----
NFFGVQLVLVALFALQDLANNPLILDPVMS----

Skeletonema marinoi MMETSP0319-20121206_1511
EADVALVANIMKRDTN--SFAPFNQLASAWIQFMT--HDWFQHDASSS--QGLK-----------------
-------------------MKNVVTHWWDASQMYGS---------------------------SQEEVDAVRAE-
GGKLHLDVNDEIDYN--------------------------------
ASLPITGFRENWWAGLHILHTIFAREHNHIVDILAQS---------YPS------
MTEDELFGTARNIIAVLLAKIHTVEWTPTLLDNAVSDMALNINWHGLQTVTSMYFKGKNIP
DEVKDIIDEMK--------------------VPSVFGSNYTTEQTLFNTP------FYMTEEFVSVYR-
MHTLLPDAMILEGGKT----------VSLQELAFTDARNLVSDPSKTTAT-------

LLQAFAHTPAQALSLKNYPKSLFNLQIGNGK----------
SINLAEIDISRDRARGIPRYNDARRQLLLT--PYKSMDDLTSDKE-----------------ELKLLKSVY-
---TDIEQVDFLVGCLVDKDRPDGF----AFGIVPYYIFVVMASRRLLSDRFFQEGLTEENYS

Pseudo-nitzschia pungens MMETSP1061-20121228_74475
DNVQVIAQRLLAREGFKPAGNQLNIIAAAWIQAQV--
HSWIQHLDGAPTSIEATAEAVGPVCPVKK--MNFFETE-ERPD----GE----
YNSFRTQWWDASFVYGQ--------------------------
NREQVHLGRLYKDGKLKVNESNPDTLSFV--------------------------
EEGKSKIDVVGDQSNSWVGVTVLQVLFIKEHNYCAEMIKKE---------NPQ------
LTDEEIYGHCRNIISALVAKIHTIDWTVELLKTEQLRVAMEINWKG-------------
ATKAVFGDKAPF--------------------HP--LRLINKPKADNKGVP------FCLTEEFAAVYR-
LHPLLPPGLVVEHG-----------EGKEEFIGIEKLLTTKGRDKMREPG---
MAKKIMFSAFHYSCGHLKSSNYPFIMRKFTPTDHKGVDLQPPEDRVVDMAAIDLHRDRER
GIQKYNEFRRQLKLR--PFKTWEALTGEENKSDN----------
ELTDAKKLELIYGPAPEGIEKIDLLVGDLYERKIADGF----
ALSETSFMIFLLMASRRIDSDPYLNEYYNEEYYT

Thalassiosira rotula TR754|c0_g1_i1|m.3431
MLFPTFAQHLIDSFIV-----TAIKENTSEG----
VEFDWRKTGSPHDIGLLPLYGKTIEQTTQLRLKS-ELKGSKGKLKTQIIKG-----
GEWSPYLFDAKGNKKP---------------------------EFSSLP-DPEGLEFALGMAKQHGVDVKK-----
----------------------N-KATVFALGGSRANLTPNIVAWNTLLLREHNRIASKIEEE---------NPK----
--WDDERVFQTARNVNLAVYLRLVIEEYINHITGF--
----GVDFTVEPGKWMWDAPWYKR------NWISAEFAVLYR-WHAVIPNCMKWG----------------
EKTLPTASYLYSNHLLLDED-GMKGDLREAFINISDHRATSMQIHNTES--------------------
WMKGRDARALQMSRACELRSFTEYCAYLGAK--VPKTFADITS-DVEVQ----------------
NELKALYGK----VENIEFWVGLIAKDHPTE-S----
IMSAELTSFVANDAFNQALTHPLLSEHVWAAGPE

Skeletonema marinoi MMETSP0920-20130426_33289
LLFPTFAQHLIDSFIN-----TKIN-VETG------EFEWDRTDSKHEIGIGPLYGDEVEQTSQLREKS-
EKPGRRGRLKTQVLEG----DEEWAPFLYNEDGTKKE---------------------------EFSAIH-
DPDGMKTILGLVYSSDPTTKS----------------------------
SIEQSIFAFGGRRANLNPNIVAWNTLLLREHNRLAGEIEKS---------EPG------
WDDERVFQTARNVLIVMYCKIVIEEYIKHIS---
GVNFKVDPGPWMWNAPWYKT------NWMSTEFAILYR-WHALIPNEAGLGP---------------
SKDAGVMESLFNNPVLLDDETGLGGNLRDIFVDISQTRITSFQLFNTEK--------------------
WMVERESAAINQGRANNVQSYAAYCEYLDIE--PPKTFEDISM-VPERQ----------------
QALKELYGT----PDRVEFYVGLIAADHPAGGK----
IFSEAMTKFVANDAFNQALTNPLLSQNVWE----

Fragilariopsis kerguelensis MMETSP0907-20130614_13332
PNVQVVAQRLLARESFTPAGDQLNIVAAGWIQAMV--
HDWMKHEDGKKTSMEVTPAVVGSQCPLHR--FNLFETK-ERPDGH--------
YNSERTNWWDASFVYGQ--------------------------
NAEQVQNSRAFVGGKLKVNEKNPDTLPSR--------------------------DDG---
TDLTGDQSNSWVGVSVLQTLFLYEHNYCAEQIAKE---------NPN------

LTDHQIYGHCRNIIAALVAKIHTIDWTVELLKTPQLKIGMRTNWMG-------------
IIQAITGLKIPF-------------------LDRLLRLIKKKENNNEGVP------FCLTEEFAAVYR-
LHPLLPPGLIIEGEGA-----GDKDEDEDTFIDLRDTLTTKGRDLMRKSG---
MAKKVMKAVFTYPCGNMAPSNYPDVMRDFHPTDLLGNNL----
DDRIDLAAIDLFRDRERGIQYFNNFRRKLSMK--
PFQTWEELTGDDKMTEEALAAFVAGTGNLTNAKKLELVYGAAPKGIEHCDLLVGDLYEK
KIP-GF----AISETSFMIFLLMASRRLDADPYLNEYFDEEHYT

Skeletonema grethea MMETSP0578-20130828_1168
ILVAYSIXSFQYVLDFKPAASQLNVLAASWIQAMV--HDWIGHFDGDET--
ETLDRGGESLCPFAKSPFSFKNTKTEKIDGVPF------SPSERTNWWDASFVYGN----------------
----------NSEQIDRARTMQGGKMVTSD-IPHALAED--------------------------KDG---
VYFAGDNKNSWVGVALLQDLFIREHNYICDQIAAE---------AKEEGK--
EMTDEELFGKARVVVAALVAKIHTVDWTVELLKTKLLAIGMKTNWDG-------------
LLKAVG-IPIPG-------------------ILSQMGEKKGRVSDNEGTP------FCLTEEFAAVYR-
LHSLSPPGLILGDG-----------DAKDKFIGLEDLLGDEGRKQMRETKT--
RPKEMMKSCLHWPCGALMSSNYPNAFRDVAPTDDYGKDLK---
SQNIDLAALDLFRDRERGILKFNEFRRQLNLK--PYRTWLELTENEE-----------------
DARKLELIYGPGQEGIERCDLLVGDMYERKVQPSF----
ALSETSFIIFLLMASRRLSADPFLNELYNEETYS

Staurosira sp MMETSP1361-20130828_23824
LLFPFFAQWFVDSFLR-----TKWKPLAEQD--------
FKENESNHDIDLNQIYGTSEIQTDMLRSMK------GGRLKSQIIDG-----EEYPVFLFDQKT------
A----------------------TLKPEFRGLYT-EENFKRVFGNASKE------------------------------
HKLHSFAVGLEHGNSTIGNTVMNTLFLREHNRVAGVISAA---------HPE------
WDDERVFQTTRNVMIVLLIKIVLADYIYHIS---
GAAVFADPGGFAEDELWYRE------NWMSVEFSLLYR-WHDLIPSSVTFDG----------------
ETRDAVDLQNNNRWLLKAG------MDSVIQDASNQKAGVMGLGNTPDF-------------------
LLSVTKMS-LHMARTCKLSSYVEYCKEYGQD--PPEDFMDLTG-DQDSA----------------
SKLERVYGS----IDKVEWFVGLFAQK-RDV------
FSGLLMTLMVGNDAVTQAFTNPLLAKRVYN----
Skeletonema marinoi MMETSP1428-20130617_31509
EADVALVANMMKRDTN--SFAPFNQLASAWIQFMT--HDWFQHDASSS--QGLK--------------
---------------------MQNVVTHWWDASQMYGS--------------------------SQEEVDAVRAE-
GGKLHLDVNDEIDYN--------------------------------
ASLPITGFRENWWAGLHILHTVFAREHNHIVDILAQS---------YPS------
MTEDELFGTARNIIAALLAKIHTVEWTP----------------------------KNIPDEVKDIIDEMK---------
-----------VPSVFGSNYTTEQTLFNTP------FYMTEEFVSVYR-MHHLLPDEMILEGGK------------
-LQELAFTDARNLVSDPSKTTAT-------LLQAFAHTPAQALSLKNYPKSLFNLQIGNGK--------
--LINLAEIDISRDRARGIPRYNDARRQLLLT--PYKSMDDLTSDKE----------------
ELKLLKSVY----ADIEQVDFLVGCLVDKDRPDGF----AFGI--YYIFVVMASRRLLSDRFFQ-----
----
Homo sapiens NP000953
LMFAFFAQHFTHQFFK-----TSGKMGPGFT-----------
KALGHGVDLGHIYGDNLERQYQLRLFK------DGKLKYQVLDG-----EMYPPSVEEAP-
VLMHY--------------------------------PRGI---
PPQSQMAVGQEVFGLLPGLMLYATLWLREHNRVCDLLKAE---------HPT------

WGDEQLFQTTRLILIGETIKIVIEEYVQQLSG---
YFLQLKFDP-ELLFGVQFQYR------NRIAMEFNHLYH-WHPLMPDSFKVGS-----------------
QEYSYEQFLFNTSMLVDYG------VEALVDAFSRQIAGRIGGGRNMDH--------------------
HILHVAVDVIRESREMRLQPFNEYRKRFGMK--PYTSFQELVG-EKEMA-----------------
AELEELYGD----IDALEFYPGLLLEKCHPN-S----IFGESMIEIGAPFSLKGLLGNPICSPEYWK--
--

Ovis aries NP001009476

LMFAFFAQHFTHQFFK-----TSGKMGPGFT-----------
KALGHGVDLGHIYGDNLERQYQLRLFK------DGKLKYQMLNG-----EVYPPSVEEAP-
VLMHY--PRGI---
PPQSQMAVGQEVFGLLPGLMLYATIWLREHNRVCDLLKAE---------HPT------
WGDEQLFQTARLILIGETIKIVIEEYVQQLSG---
YFLQLKFDP-ELLFGAQFQYR------NRIAMEFNQLYH-WHPLMPDSFRVGP-----------------
QDYSYEQFLFNTSMLVDYG------VEALVDAFSRQPAGRIGGGRNIDH--------------------
HILHVAVDVIKESRVLRLQPFNEYRKRFGMK--PYTSFQELTG-EKEMA-----------------
AELEELYGD----IDALEFYPGLLLEKCHPN-S----
IFGESMIEMGAPFSLKGLLGNPICSPEYWK----

Homo sapiens NP000954

MMFAFFAQHFTHQFFK-----TDHKRGPAFT-----------
NGLGHGVDLNHIYGETLARQRKLRLFK------DGKMKYQIIDG-----EMYPPTVKDTQ-
AEMIY-------------------------------------PPQV---
PEHLRFAVGQEVFGLVPGLMMYATIWLREHNRVCDVLKQE---------HPE------
WGDEQLFQTSRLILIGETIKIVIEDYVQHLSG---
YHFKLKFDP-ELLFNKQFQYQ------NRIAAEFNTLYH-WHPLLPDTFQIHD-----------------
QKYNYQQFIYNNSILLEHG------ITQFVESFTRQIAGRVAGGRNVPP--------------------
AVQKVSQASIDQSRQMKYQSFNEYRKRFMLK--PYESFEELTG-EKEMS-----------------
AELEALYGD----IDAVELYPALLVEKPRPD-A----
IFGETMVEVGAPFSLKGLMGNVICSPAYWK----

Coccotylus truncatus AFN20596

VLIAYYAQWVTHQFFN-----TDESDPTGHS-----------
VKQPVGVNMSMLYGSKQEVEKSVRAYK------GGLLKSTIKNG-----QEFPEIMPCQEGSRIP--
----
GKEMFNMPILIANMIPGFAAIHVLFFRRHQYICRELAKW---------AEAQGK--
NIDDEELFQKAKLIVTVNMLRITMHDYVSRALQSSHAKMR--
----------FDQKVKQSRIWKMFGPDYFPPS-----NAIQFEFNIFYR-WHQFYPDTTKIMKR--------
IDDLKFPKSKQQLDEKWNAVRWIADE---PDGMERVLFSASSQRAGKLSLLNTNQW----------
---------IVEHVVKPGLARCREHQLASYNDYREKVGFP--RLTTFEQVTS-NPALL-----------------
EKLKRVYRN----VDQIEYYPGVFAEDKHFG-------
NVHGPLTFGSSMTFTGIFSSRLFETALDE----

Ciona intestinalis XP002127674

VLFPFFAQHFTHMFFK-----TDPMKGMPYQ-----------WGD-
QLVDLSQIYGHGEKRQHELRSHV------NGKLKVSLVDG-----HEFPP-LSNQTTANMSN--------
----------------------------INLL---
PQEYQFVFGHQGFSLMPTFLIWSTIWLREHNRICDLIKEE---------NPA------

WDDERIFQTARLVLTGETIKVVIEDYVQHLSG--
FHYKLLYDP-ELVQGGSHSFH------NQIHVEFQLLYH-WHALMPDQIEFNG---------------
KSYTMKRLLFNPEPVVKGG------LKRTIEDLSNQWAGQVAGGKTQGA------------------
ATLHVAGLAIKNGRDLRMQSFNAYKEKFEMK--KYTTFQELTG-EEEMA---------------
AELQKLYGD----IDAVEYYIGIMLEKRRSP-Q----
LFGETLTEMGSPYSLKGLYSNPINHKDWWK----

Ciona intestinalis XP002123273
VFFAFFAQHFTHQFFK-----TNTIKGMPFQ-----------WGE-HSVDLSHVYGHTIQRQHELRSHI--
----DGKLKVFETNG-----EVFPP-LTESANVTMSG---------------------------------EKLM-------------
------------------------------RGR-KFAIGHPGFGAFPSFFVIATLWLREHNRVCDILKDL---------
HPD------WDDERLFQTARLILTGETLKIIVEDYVQHVSG---
------------FHFQLSYDP-EILHKSTFSYN------NQIHAEFHILYH-WHMLMPDFIELGE-------------
--HVYPLKELLFNVDPVVEIG------METVLKQLSNQFAGKVVGGRNQGP------------------
ELVAVVELALKQTRQMRMCSFNKYRERFGMK--PYTSFEELTG-ETEVA---------------
ALLRNLYYD----IDALELFVGYFVEHRRNR-Q----
VLGATMLEMGAPYSLKGVFGNPIGSPAWWK----

Oncorhynchus mykiss CAC10360.1
LMFAFFAQHFTHQFFK-----TRNSMGLGFT-----------
RALGHGVDAGNVYGDNLVRQLNLRLLK------DGKMKYQVVKG-----EVYPPTVAEAA-
VNMRY---------------------------------PQET--
PVGQRMAIGQEVFGLLPGLTMYATLWLREHNRVCDILKAE---------HPT------
WGDEQLFQTARLIVIGETIRIVIEEYVQHLSG--
YLLDLKFDP-VLLFKSTFQYR------NRIAVEFKQLYH-WHPLMPDSFHIDG---------------
DEVPYSQFIFNTSIVTHYG------VEKLVDAFSRQCAGQIGGGRNIHP------------------
VVTNVAEGVIEESRTLRLQPFNEYRKRFNLK--PYTSFSDFTG-EEEMA---------------
RELEELYGD----IDALEFYPAIMLEKTRPN-A----
IFGESMVEMGAPFSLKGLLGNPICSPEYWK----

Fistulifera solaris GAX23950.1
LPPVKDVAAILRRPMNPTSVAPFNQIAVAWIQMMT--HDWFQHDPAHPDQK------------------
---------------------MNRVTHWWDASQLYGS--------------------------
TLAQQTAVRVPNTGKVRLDQHQELNYTT-------------------------------
TGIPITGFADNWWAGLHMMHTLFVREHNWLVDQFERQ---------YPG-----
VYTANDKFQLARLCLSALLAKIHTVEWTPTLLDNPVAALGLHTNWRG---
VDAILEYGTRFELQLAYRIVGGD--------------------QSVPHAGNGTTRETLYNTT------
FAMTEEFVAVYR-MHPLLPDEMEIEGKTFS-----------LNDLSFVDARTLTKS-VKTTQT-------
LLQAFGMTPANTLSLQNYPRQLYGLEKPGMS---------
QPVNLAEIDLQRDRERNLPRYNDMRRQLLLK--PYKRLEDLTDDET----------------
ELNLLKSVY----QDMDQVDLMVGCLVDKDRPYGF----
AFGIVPFHVFLVMASRRILNDRFFMEDFNAKVYT

Cyanothece sp ACB53655.1
PNPRVVSRTLMTR-EDFKPATILNLLAAAWIQFEN--
HDWFSHGDNKPEDKLEIPLEANDPWPEEYRPLEVGKTLPDTSRPEGAKPP--
TFINTVTHWWDGSQIYGS--------------------------NPETVDQLRSHEDGKLIIGENGLLPVDP----
---------------------------ETGVDITGFNDNWWIGLGMLHTLFTREHNLICDHLKQE---------YSQ---

---WSDDDLFDHARLINAALMAKIHTVDWTPAILPLPATDIALNVNWNGFLG------------
EDIKQVLGTVG----------------EGEIADLLTGIIGS-DKNHHTAP------YYLTEEFVSVYR-
MHPLIPDELEFRSLEGD---------KFLQEVNFFEMSGKRTRALLESISL----
PDLFYSFGITHPGAITLHNYPRFLQQLVRDNGE----------
VFDLAAVDILRDRERGVPRYNRFREIMGRG--RVKCFEEISSNKQ----------------
WVEEMRRVYN---DNIDQVDLMVGLFAEDT-PEGF----
GFSDTAFRVFILMASRRLKSDRFFTTDYRAEIYT

Fistulifera solaris GAX18717.1
LPPVKDVAAILRRPENPSTVAPFNQIAVAWIQMMT--HDWFQHDPTNPDSK--------------------
-------------------MNRVTHWWDASQLYGS--------------------------
TLTQQRAVRVPNTGKLRLDKHQELNYTS-------------------------------
TGIPITGFADNWWAGLHMMHTLFVREHNWLADQFELQ---------YPG-----
VYTANDKFQLTRLCLSALLAKIHTIEWTPTLLDNPVAALGLHTNWRG---
VDAILEYGTQFELQLAYRIVGGD--------------------QSVPHAGNGTTRETMYNTT------
FAMTEEFVAVYR-MHPLLPDDMEIEGITLT-----------LNDLSFVDARKLTKS-VKTTQT-------
LLQAFGTTPANTLSLQNYPRQLYGLEKPGMS---------
QPINLAEIDLQRDRERNLPRYNDMRRQLLLK--PYKRLEDLTDDET-----------------
ELNLLKSVY----QDIDQVDLMVGCLVDKDRPYGF----
AFGIVPFHVFLVMASRRILNDRFFMEDFNAKVYT

Alcanivorax nanhaiticus WP_035233415.1
PNPREVSNLIMSRGGDFKPATTLNFIATSWIQFMV--
HDWFDHGPRTDANPIEFPLPAGDVLGGGTMSVQRTRPDPDVSGDEGIITY--ENIN--
THWWDGSQLYGS--------------------------SKEKNDEVRSFVDGKLKVDGDGRLPTEF------------
-------------------FSGKPVTGFNENWWVGLSMLHHIFTQEHNAIADMLVAN---------YPG------
QSDQWYFDKARLINSALMAKIHTVEWTPAILANPVLERAMYANWWGLGGDRDKRDKYQ
DDLDNLNNNLGQIGGLLDLVGIDNGLGDSPTGSLEHALAGLVGSRTPNNYNVP------
YTLTEEFVSVYR-MHPLLRDEIKVYDIGSN---------VVDEEIALEDTRNGDAEDLLGDIGQ----
DRLWYSFGITHPGALTLNNYPDFLRNLSMPLIG----------
DIDMAAIDILRDRERGVPRYNEFRRQIGLK--PLTSFEQLTSDPQ----------------
LLADLKALYN---NDIELVDTLVGQLGEETRPEGF----
GFGETSFQIFILNASRRLMTDRFFTTDYTDEVYT

Hordeum vulgare subsp. Vulgare BAJ90503.1
PDPFVVATKLLARREYKDTGKQFNILAAAWIQFMV--
HDWMDHMEDTKQIEITAPKEVANECPLKS--FKFYATKEQPTNSDGIKT---
GYHNIRTAWWDGSAVYGN--------------------------NEKQEKKIRTYADGKLVIGDD-GLLLHE-
--------------------------ENG---VPLSGDVRNGWVGISILQALFVKEHNAVCDAIKEE---------
HPN------LSDEELYRYAKLVTSAVIAKIHTIDWTVELLKTKTLRAGMRANWYG------------
LLGKKIKDTFGHIG--------------------GTALGGLVGLKKPINHGVP------YSLTEEFTSVYR-
MHPLIPSTLKLRDPTGQPA--ADNSPPYLEDIDIGELVGLKGEDQLSKIG----
FEKQTLSMGYQACGALELWNYPSFFRDLIPQNLDGTNRS----
DRIDLAALEVYRDRERSVPRYNEFRRRLFLI--PIKCWEDLTSDND----------------
AIEAIRAIYG---DDVEKLDLLVGLLAEKKIK-GF----
AISETAFNIFILMASRRLEADRFLTSNFNEKTYT

Triticum aestivum CDM84254.1
PDPFVVATKLLARREYKDTGKQFNILAAAWIQFMV--
HDWMDHMEDTKQIEITAPKEVANECPLKS--FKFYATKEQPTNSDGIKT---
GYNNVRTAWWDGSAVYGN--------------------------NEKQAEKTRTYVDGKLVIGDD-
GLLLHE---------------------------ENG---VPLSGDVRNGWVGVSILQALFVKEHNAVCDAIKED-
--------HPN------LSDEELYRYAKLVTSAVIAKIHTIDWTVELLKTKTMRAAMRANWYG-------
-----LLGKKFKDTFGHIG-------------------GTALGGLVGLKKPINHGVP------YSLTEEFTSVYR-
MHSLIPTTLKLRDPTGQPA--ANNSPPYLEDIDIGELVGLKGEDQLSKIG----
FEKQTLSMGYQACGALELWNYPSFFRDLIPQNLDGTNRS----
DRIDLAALEVYRDRERSVPRYNEFRRRLFLI--PIKSWEDLTSDKD----------------
AIESIRAIYG---DDVEKLDLLVGLMAEKKIK-GF----
AISETAFNIFILMASRRLEADRFITSNFNEKTYT

Rhizophagus clarus GBC07128.1
LELEHYITALTQLPLN----
TPEEKKLLSILERLLVTQLWSDISKPPAMIAGDIYRSSDGSGYNRLIQSLGKANSRYSLTSRI
QYPIKLSVLPKSEDIFDKIMVQGGDFVEHPSGISSMLFYLAIIITHDLFHTSFADPNINLTSSYL
DLTPLYGSNDQEQKSIRTLKG--------------------
GLLKPDTFADSRILLQPPGVSALVILFSRNHNFIAQTLLEKNELGRFSVTNPNDPEQLKKQDE
DLFQTARLINCGFYINVILHNYLRTILGLDR---
TNSKWFVDPTVPYNKRGQLEPLPSGIGNIVSLEFNYIYR-WHAATSKDDSKFVEDEFKTIFG-
-----
DDWENITIDEFKEKMGVWGRSIPKDPSKWKFNHIERGSDNRFKDTDIAKEIINGTKKVSGA
FGANRIPKVFRPIELLGIESARVLGLSSLNDFRRSLNLK--PYESFMEMNP-DPLIA--------------
-KKLEELYGS----
IENVELYPGLMTEKTKPDMLGSAIALPFTISRAILSDAVNLVRNDRYYTNDFSPRNLT

Emiliania huxleyi XP_005780718.1
PFQALHNLPSADEVVR------LLYKRDAFK------------
KAPYGVNSLATWFANVAIHDFFRTAT----GTDGGTHPERGSDK--
EWVNLHSSYLDLQPLYGYS---------------------------------KTTADATREWS------------------------
--------------GGKLKAFAEDRMRRIPESRVIVELLRREHNYVAEQLAQR---------YPAQ----
FATDEELYQQARLIMGGVYINIILRAYGCQMFG---
----EIAPDGSGFCELRQGYGGAGVG---NMCTFNFNLIYR-FHTSIPVEWSATD--------------
PPPIDTDEQMR-----------------TLLNGILNWESGGFGPNNVPDSILG----------------
ERARVSQRAIEAARLMGAPTLNTFRRRFTSG---YSSFEDMTGGDQATA----------------
DTLRQLYPG---GIEDVELVVGCQVEKCMSGGW----
ALPSTIGQAIVADAFASIRQDRFYTQDWGASSYT

Rhodococcus gordoniae WP_064063209.1
VWFAFFAQWFTDSFLR-----TNSKD------------
PRKNDSNHEIDLCQIYGVNQAKTTMLRAGY------GGRLDSQVIDK-----
KEYPPFLFAARTPGEEL-------------------------RFVPKFEGLHDREYLLDTVLRLCPDE-----------
-------------------RKKSVFAVGLEHGNSTIGNTVLNVLFLREHNRIAGILEGA---------YPEWD---
---DDRLFETTRNIMIVILLKIVIEEYIKHIG---
PFDFPIEFVP-FMADNAPWNRT------NWCAIEFNLLYR-WHSLIPDTVVFDS----------------
QRVSTRILVDNNPLVLDRG------IESIIDQCSRQKASRIGLGNTPAFLIDRHPMCPD---------
RESVEERT-VGLMRQARLRSFNDYREAFGLG--RLTSFTELTG-DVEVQ----------------

QKLARLYGD----VDAVEWYVGIFAED-YPRHR----
MMGELLTTMVAHDAFTQAFTNPLLARHVYHEDTF

Herbidospora mongoliensis WP_066371138.1
VMFMFFAQWFTDSFLR-----TSRDD------------FRRNTSTQEIDLCQIYGLTEEKTHLLRAHR--
----DGRLKSQLIDG-----EEYPEFLFRPRSSGEPP-------------------------
VFKPEFEGLHDSEFIVSRLLEDAPEK------------------------------
QKDTFFAVGLEHGNSTIGNTIMNVVFLREHNRIAGLLKQA---------
HPEWAERPEGADARLFETTRNIMIVLLLKLVVEEYIRHIS--
-------------PYDFPLETVP-FIADGKRWNRS------NWISIEFNLLYR-WHSLVPSTIGSGP-----------
-----DRLDSTDFRNNNPLVLARG------IESLVSQCSGERAGRIGLMNTPGFLVDRDPAHPE------
---RPSVQERT-IALSRKARLASYNDYRENFGLG--RLKDFGRLTQ-DAELR----------------
ERLEKLYDD----IDKLEWYVGIFAED-YSRDE----
MMGRLMTTMVAYDAFTQALTNPLLARDVYNEATF

Eutrema halophilum BAJ34623.1
---MVVATKLLTRRKMIDTGKQFNMIAASWIQFMI--
HDWVDHLEDTDQIELSAPKEAAKGCPLSS--FRFFKTKEVPTGFFEIKT---
GSLNTRTPWWDSSVIYGS--------------------------NSKTLERVRTYKDGKLKISEETGLLLHD---
------------------------DDG---LAISGDIRNSWVGVSALQALFIKEHNAVCDLLKKE---------YED--
----LEDEDLYRHARLVTSAVIAKIHTIDWTVELLKTDTLLAGMRANWYG------------
LLGKKFKDTFGHVG-------------------SSIFGGVVGMKKPQNHGVP------YSLTEEFTSVYR-
MHSLLPDQLHMRDIDVTPG--PNKSLPLTQEVSMEKLIGREGEETMSQIG----
FTKLMVSMGHQACGALELMNYPAWFRDLVPQDPNGHDRP----
DHIDLAALEIYRDRERNVARYNDFRRAMFMI--PIKTWEDLTDDKE----------------
AIELLDDVYG---GDVDELDLLVGLMAEKKIK-GF----
AISETAFNIFLLMATRRLEADRFFTSDFNEMTYT

Pisum sativum CAH05011.1
PDPMVVVTKLLERKTYKDTGTQFNVIAASWIQFMI--
HDWIDHMEDTKQVELSAPSEVASQCPLKS--FKFFKTKEIPTGFYDIKT---
GHANVRTPWWDGSVVYGS--------------------------NEQVLNKVRTFKDGKLKISKE-
GHLLHN---------------------------EDG---TAISGDIRNSWAGVTTLQTLFVQEHNAVCDALKKE-
--------NSD------LEDEDLYRHARLVTSAVIAKIHTIDWTVELLKTDTLLAGMRANWYG--------
----LLGKQFKDRFGHVG-------------------NSILSGFVGMKRSENHGVP------YSLTEEFATVYR-
MHPLLPDSLHLRDISASPG--PNKSPPLIKEIPMNDLIGLQGEKTLLEIG----
NAKKLVSMGHQACGALELWNYPSWLRNLVPHNIDGTERS----
DHVDLAALEVYRDRERNVARYNQFRRGLLLI--PISKWEDLTDDEE----------------
AIKVLEEVYG---DDVEELDVLVGLMAEKKIK-GF----
AISETAFVIFLLMASRRLEADRFFTSNFNEETYT

Original sequences, ungapped, utilized in the phylogenetic analysis

Chaetoceros neogracile MMETSP0751-20121128_3624

MVFPNFAQFLTDGFAKSIPGTNFLRNNATHEADLCQIYGRNKDQTDCLRLMSSSIGE
KGRLKSCMINGEEWPMPYFLESGEVDPQFERLDPPKFSFDHVIEQLSRIDPNGRLGLVQKIK
KNIFAVGSDRGNTTPGVAAFASLFLREHNRLAAEVENRNPAWDDGQIFQTARNINIVIYIRII
MEDYLNQLSHAPFDFKLDPGPWTWDAKWNKKFRVAVEFSTVYRWHSIIPNAIQIGEGDPL
PIMQAMFNNELLIDRTLLKSFEDSSSQRATSFTPFNTADIMLPREYNTILQARQAGIRYYVD
YLKQFGFPVDLPKNYSDITSDEKVQKMLEEMYGKGNVDKVEFYIGAICATHERNAPFSLF
MNLQVAHDAIKAFYMNPLLQRSSWK

Asterionellopsis glacialis MMETSP0707-20130614_6459

MILPTFANHLTGGFIKSTRGPDGSYQYGRTHSNHNIDLLQLYGRTQEQTLALRLRDN
TTFGKKGRLKSQILNGGEEWPPFLYKDGRIDPQFEVLDPIAHGMSHLVDIMEGDRSKFFAM
GSNQQNMLPQNIAMTTLFLREHNRVAGELERRYSDWDDDRIFETARNIVIVIYLKIVIEEYI
NHISPLPDIVKFLVDPGPWMWNAEWNKPNWISAEFAILYRWHALMPNKVMHAGIVFDLSS
TLFQHDLLLGEDRSLKKTFVDMSAQRSASCECLNTAGPLVEREVESLRYSRALRFRPYVEY
VEYWGLKKPRRFEDITKNAEVASMLRDLYGTVDKVEFFVGLIASDHSKNGIFGSSMNIGVG
LDAFSQALTNPLLSEHVWK

Aulacoseira subarctica MMETSP1064-20121228_17645

MLFPVFAQMLIDSFISTVTYTNTTTKKLTFDWKRTNSPFEINLLPLYGRFEYQTDALR
LKSEKSRGRLKSQIINGEEYAEFLYNDLGEIKDEFQVLGPPQAFNQILSKVDKTEEEKRKIKS
KIFAFGGNRTNTTPQMAALNTLFLREHNRLAGQLEKHNEDWDDDRVFQTARNINLVIYLKI
IIEEYINHITSSGARFKVQPKEWVWNADWNKPNWIAVEFAVLYRWHALTPNSYLWEGKRV
KISDDLFNNALLLETSGGLRQAIAEISKNPATIMAPFNTALELLSQEQAAFAQTRQANVRPF
ADYRAYLGLPLVKTFKDITQDKEVQQKLEELYKTPDRVEFWAGLVAEDKDPKAIFGPTLS
TLVALDAFSQALTHPLLSEQVFN

Aulacoseira subarctica MMETSP1064-20121228_57453

MLFPVFAQYLIDSFILTKRIKTEGGTVSTIDWKRSDSPNDIVLLPLYGFKNATDALRL
KSEEAQKRGKLKSQLIDGEEYAPFLYDESGMVKEEFKALGEPESLGEILKAMMADSNTVK
KYKSKIFAFGLVRVNITPQLAAMNTLFLREHNRLAGVLEENNPEWDDERVFQTARNINLVI
YLKIVIEEYINHISSGVQFKAKPEKWIWHASWNKPNWISVEFAVLYRWHTLVPNSDEWNGI
RYDFQNEIFNNEILLDAGGLRHSFASISANRAPSICPFNTGGFLLPREASALQQSRVNKLRPF
GEYREYLGYKKTKEFSDISSDPQVQETLRKLYKTPDRVEFYAGLIAEDHVAKGILAPIMTDL
VAKDAFTQALTHPLLSENVFN

Chaetoceros affine MMETSP0090-20130426_9955

MLFPTFAQHLIDSFIVTAVKSDGGSGTEFEWKKTDSPHDIGLLPLYGRTFDQTKQLR
VQNPPRGKYGQLKSQIIHGEEYAPYLYDADGKVKKEFDLLETPQGLERSLSMLSPEDAKAK
KSNIFAFGGARTNLVPNITAWNTLLLREHNRIAQTIEKEEPTWDDERVFQTARNVLLVIYLK
LVVEEYINHITGYGIDFTVDPGKWMWNAPWYKRNWISAEFAVLYRWHGVIPSCMKWGD
KTLSTHESLFNNAVLTEDMKGSLRDTFINISNHRATQMNLFNTESMMVLRDMAALKQCRA
CKIKPYADYVVYLGTKERPTKFSDISKDKEVQEALEKVYKKVENVEFWTGLLASDNPPEGI
MSPEMTTFVANDAFNQALCHPLLSENVWS

Chaetoceros curvisetus MMETSP0717-20131115_51459

MLFPTFAQHLIDSFIVTAIKENSSEGVVFNWRKTGSPHDIGLLPLYGKTIEQTTQLRL
KSEVKGSKGRMKTQIIKGGEWSPYLFDSKGNKKAEFSSLPDPEGLEFALMEAKKFGVDAE
KNKASVFALGGSRANLTPNIVAWNTLLLREHNSIAAKIEAENPEWDDERVFQTARNVNLA
VYLRLVIEEYINHITAFGVDFTVEPGKWMWDAPWYKRNWISAEFAVLYRWHAVIPNCMK
WGEKTLPTAAYLYSNHLLLDDTGLKGDLREAFINISNHRATSMEIHNSEKWMTGRDSRAL
QMSRECELRSFTEYCAYLGKPVPKTFADITSDVEVQNELKALYGKVENVEFWVGLIAKDH
PTEAIMSAELTTFVANDAFNQALTHPLLSEHVWP

Chaetoceros debilis MMETSP0149-20130528_3320

MLFPTFAQHLIDSFIDTVYHYDDDGNVVFDWKRTETPHDIGLLTLYGKTIPETKQLR
KQSETSGEKGKLKSQLVNGEEWAPFLYDSNGKVKEEFNELPVPQGIDEKMYAARPQVQA
KLKESIFAFFGGSRTNLTPNISAWNTLLLREHNRIAGLIEEENPTWDDERVFQTARNCTLVIY
LRLVIEEYINHITAYGVDFEIEPEKWMWDAPWYKRNWISAEFAVLYRWHAVIPSLMKWGK
NTHTTMDYLFSNNLLLSDDGMKGNLRDCFHNICDHRATNMQLHNSEGGFMVGRDKSALE
MSRSCKLRSFSEYCGYLGTPAPESFADITQDKDLQKELKDVYGEVKNVEFWTGLIAKDHSC
EAIMSAELTKFVANDAFNQALTHPLLSEHVFN

Chaetoceros debilis MMETSP0150-20130528_3844

MLFPTFAQHLIDSFIDTVYHYENDGNIVFDRKLTGTPHDIGLLTLYGKTIPETKQLRK
QSDTSGEKEKICPNLLKEKSXAPFLYDSNGKVKEEFNKLPVPQGIDGKMYAARPQVRAKL
KESIFAFFGGSRTNLTPNISAWNTLLLREHNRIAGLIEEDNPTWDDERVFQTARNCTLVIYLR
LVIEEYINHITTYGVDFKIEPEKWMWDAPWYKRNWISAEFAVLYRWHAVIPSLMKWGKN
THTTMEYLFSNNLLLSDDGMKGNLRDCFHNICDHRATNMQLHNSEGGFMVGRDKSALEM
SRSCELRSFSEYCGYLGSPAPESFADITQDKDLQKELKDVYGEVKNVEFWTGLIAKDHSCE
AIMSAELTKFVANDAFNXGFDSSSPFR

Odontella MMETSP0015_2-20120614_17751

LLFPTVAQHLIDSFINTKYTKTINGTFFDWARTDSPHEIGLSPLYGCNPKQTSQLREL
NEKIGCKGRLKTQILGDGGEEWAPFLYGDSGKIKAEFNELDEPVALDHILGFNSENAEMMR
KSIFAFGGSRANLNPNIVAWNTLLLREHNRVASKIEASEPSWDDERVFQTARNVVLVIYLKI
VIEDYVAHISGVNFKVQPGEWMWNAKWYKRNWMSVEFAVLYRWHALIPNTTFWGSKTA
STKDALYNNTLLLNKEQGCAGNLRNIMVQISEQRITSFQLNNTEDWLVDRDLNALRQSRE
CNLASYADYCEYLGMDRPKTFTDISLYPEVQEKLRSLYKTADKVEFYVGLIAADHSPGGK
LFSEAMTRFVANDAFNQALANPLLSENVWK

Cyclotella meneghiniana MMETSP1057-20121228_4220

LLFPTFAQHLIDSFINTRIDHEATEKNGGRPVFDWARTDSKHEIGLSPLYGDTEEQLN
QLRLLSNVNGFKGLMKTQIINEEEWAPFLYEVDGSKKKEFSAIWDPSGASYVLGMRRDVG
ERQKRTLFAFGGARANLNPNIVAWNTLLLREHNRIAGEIEKSEPSWDDERVFQTARNVLVV
IYLKLVIEEYIAHIAGVNFKVDPGEWMWNAPWAKTNWMSTEFAILYRWHAVIPNTSSWG
KAKNLKVLDTLFNNDLLLDTKEGLSGNLRDAFVSISEERVTAHQLFNTEEWMVDRELAAI
KQGRANKVASYADYAEYVDLPRPKTFADISLYPEVQKALEEVYGTVDRVEFYVGLIAVD
MGAGGKIFSPMTKFVANDAFNQALTNPLLSMNVWN

Cyclotella meneghiniana MMETSP1057-20121228_12622

LLFPTFAQHLIGSFINTKIDHEATEKNGGQVIFDWAKTYSRHDIGLSPLYGDTTEQTD
QLRLMSNVTGFKGRMKTQLINDEEWAPFLYRVDGTKKPEFNAIWDPAGASHVLKMRGEE

GFKQKRTLFAFGGARANLNPNIVAWNTLLLREHNRLAGEIEKSEPSWDDERVFQTARNVNI
VIYLKLVIEEYIAHISGADFKVDPGEWMWNADWNKANWMSVEFAILYRWHAIIPNTINWG
TSNMKVSDILFNNDLLVKETEGLDANLRDVFVQMSEQRATAHQLFNTEEWLLDRELAAVT
QGRANKVASYADYVEYLNLPRPKTFADISLYPEVQNALKEVYGTVARVELYVGLIAADM
GAGGKIFSLMTKFVANDAFNQALTNPLLSQNVWK

Pseudo-nitzschia arenisensis MMETSP0329-20121206_15499
LLFPTVAQHLIDSFINTHVDRKATEAKGSPVFNWAKTDSPHEIGLSPLYGDSKEQTD
QLRERSEEVGRKGRLKSQMIEGEEWSPFLYDSKGNKKTEFSKIPDPSGMSHILKHVNAATA
KKASIFAFGGARANLNPNIVAWNVLLLREHNRLASMIEESEPTWDDERVFQTARNVSIVIY
LKIVIEEYIKHISGANFRVAPGKWLWNAQWNKTNWMSVEFAILYRWHAIIPNSSSWGPSKD
VEVRESLFNNTLLLDKTKGMGAKLADIFVQISNERTTSFELNNTEKWLVDREMAAIKQGRT
NNVAPYADYCEYLGYERPKTFADINRDPKVQEQLKELYGTPDKVEFYVGLIAGEHPSGGKI
FSKMTSFVANDAFNQALGNPLLSQNVWE

Thalassionema frauenfeldii MMETSP0786-20121207_3776
LLFPTFAQHLIDSFINTRIKADTDKNKPPEFEWTRTDSKHEIGISPLYGDEPAQTKQLR
ELSETSGRKGRLKTQMIEGEEWAPFLYNGNNKKPEFSDIPEPDGVRMIEKHGFGDRSTIFAF
GGRRANLNPNIVAWNTLLLREHNRLAAEIEKSEPEWDDERVFQTARNVLTVIYSKIVVEEY
VGHISGVPFEVQPGPWMWNAEWNKTNWMSVEFAILYRWHALIPDTIRWGPKVDIGIMKQ
LFNNTLLLSKENGMGANLKDCFTEISRNRVTSFELFNTEGSYMATREMQAIRQCRAANVAP
FADYCEYLGDPRPKTFEDISRKPEVQKVLKELYGTPDRVEFYVGLIAQDHSAGPKIFGDVM
TKFVANDAFNQALANPLLSQNVWE

Skeletonema marinoi MMETSP0320-20121206_19182
LLFPTFAQHLIDSFINTKLNTETGQFEWDQTESKHEISLGPLYGDDVGQTNQLREKSE
VTGRRGRLKTQILDGGEEWAHFLYDETGTKKEEFNLIRDPDGMKHILKALYNSDPAAKSSI
MQTIFAFGGRRVNLNPNMVAWNTLLLREHNRLAGEIERSEPSWDDERVFQTARNVLIVIYL
KLVIQEYIGHISGVKFKLDPGEWMWNAPWYKTNWMSTEFSILYRWHALIPNQNGLGPSKD
LGVLKSLFNNPLLLDEEKGLGGNLRDVFVDICKARITSFQLFNTEKWMVGREAATISQGRA
NNVQPYAKYCEYLRIKPPKTFKDISLVPEVQQALEELYGTPDRVEFYVGLIAADHAPGKIFS
LMTKFVANDAFNQALTNPLLSQNVWE

Skeletonema costatum MMETSP0013_2-20120614_556
LLFPTFAQHLIDSFINTKLNTETGKFEWDQTESKHEISLGPLYGDDVGQTNQLREKSE
VTGRRGRLKTQILDGGEEWAHFLYDETGTKKEEFNLIRDPDGMKHILKALYSSDPAAKSSI
MQTIFAFGGRRVNLNPNMVAWNTLLLREHNRLAGEIERSEPSWDDERVFQTARNVLIVIYL
KLVIQEYIGHISGVKFKLDPGEWMWNAPWYKTNWMSTEFSILYRWHALIPNQNGLGPSKD
LGVLKSLFNNPLLLDKEKGLGGNLRDVFVDICKARITSFQLFNTEKWMVGREAATINQGR
ANNVQPFAKYCEYLRIKPPKTFKDISLVPEVQQALEELYGTPDRVEFYVGLIAADHAPGKIF
SLMTKFVANDAFNQALTNPLLSQNVWE

Detonula confervacea MMETSP1058-20130122_27510
LLFPTFAQHLIDSFINTKTKPLESGRLGFEWDQTFSKHEIGLAPLYGDYEEETLQLRE
KSEASGRKGRLKTQVLDGGEEWAPFLYDSEGTKKPEFSLLPDPDGLQHILAMQPETMEKK
KKSIFAFGGRRANLNPNIVAWNTLLLREHNRLASEIEKSEPSWDDERIFQTSRNVVIVMYCK
IIIEEYIKHISGVNLRVEPGEWMWNASWYKPNWISTEFAILYRWHALIPNTSSWGASKNIEV
VDALFNNNLLISKDTGMGGNLRDAFVEISKTRITSFELFNTEKAMVQRETAAIEQGRFNNV

ASYADYCEYLELKRPETFEDISLKPEVQQALKELYGSPDRVEFYIGLIAADHPAGGKVFSAA
MTKFVANDAFNQALTNPLLSQNAWN

Skeletonema marinoi MMETSP1039-20121108_4563
LLFPTFAQHLIDSFINTKINVETGEFEWDRTDSKHEIGIGPLYGDEVEQTSQLREKSEK
PGRRGRLKTQVLEGGEEWAPFLYNEDGTKKKEFSAIHDPDGMKTILGLVYSSDPTTKSSIE
QSIFAFGGRRANLNPNIVAWNTLLLREHNRLAGEIEKSEPGWDDERVFQTARNVLIVMYCK
IVIEEYIKHISGVNFKVEPGPWMWNAPWYKTNWMSTEFAILYRWHALIPNEAGLGPSKDA
GVMEALFNNPMLLDDETGLGGNLRDIFVDISQTRVTSLQLFNTEKWMVERESAAINQGRA
NNVQSYAAYCEYLDIEPPKTFEXISMVPERQQALKELYGTPDRVEFYVGLIAADHPAGGKI
FSEMTKFVANDAFNQALTNPLLSQNVWE

Skeletonema marinoi MMETSP0319-20121206_26582
LLFPTFAQHLIDSFINTKINVETGEFEWDRTDSKHEIGIGPLYGDEVEQTSQLREKSEK
PGRRGRLKTQVLEGGEEWAPFLYNEDGTKKKEFSAIEDGTKKEEFSAIHDPDGMKTILGLV
YSSDPTTKSSIEQSIFAFGGRRANLNPNIVAWNTLLLREHNRLAGEIEKSEPGWDDERVFQT
ARNVLIVMYCKIVIEEYIKHISGVNFKVEPGPWMWNAPWYKTNWMSTEFAILYRWHALIP
NEAGLGPSKDAGVMEALFNNPMLLDDETGLGGNLRDIFVDISQTRVTSLQLFNTEKWMVE
RESAAINQGRANNVQSYAAYCEYLDIEPPKTFEDISMVPERQQALKELYGTPDRVEFYVGL
IAADHPAGGKIFSEMTKFVANDAFNQALTNPLLSQNVWE

Skeletonema marinoi MMETSP0562-20121206_1237
LLFPTFAQHLIDSFINTKINAETGEFEWDRTDSKHEIGIGPLYGDEVEQTSQLREKSEK
PGRRGRLKTQVREGGEEWAPFLYNKDGTKKEEFSAIHDPDGMKTILGLVYSSDPTTKSSIE
QSIFAFGGRRANLNPNIVAWNTLLLREHNRLAGEIEKSEPGWDDERVFQTARNVLIVMYCK
IVIEEYIKHISGVNFKVEPGPWMWNAPWYKTNWMSTEFAILYRWHALIPNEAGLGPSKDA
GVMEALFNNPMLLDDETGLGGNLRDIFVDISQTRVTSFQLFNTEKWMVYRESAAINQGRA
NNVQSYAAYCEYLDIEPPKTFEDITMVPERQQALKELYGTPDRVEFYVGLIAADHPAGGKI
FSEAMTKFVANDAFNQALTNPLLSQNVWE

Skeletonema marinoi MMETSP1428-20130617_783
LLFPTFAQHLIDSFINTKINAKTGEFEWDKNDSKHEIGIGPLYGDEVEQTNQLREKSE
APGRRGRLKTQVLEGGEEWAPFLYNEDGTKKKEFSAIHDPDGMKTILGLVYSSDPTTKSSI
EQSIFAFGGRRANLNPNIVAWNTLLLREHNRLAGEIEKSEPGWDDERVFQTARNVLIVMYC
KIVIEEYIKHISGVNFKVDPGPWMWNAPWYKTNWMSTEFAILYRWHALIPNEAGLGPSKD
AGVMEALFNNPMLLDDKTGLGGNLRDIFVDISQTRVTSFQLFNTEKWMVYRESAAINQGR
ANNVQSYAAYCEYLDIEPPKTFEDITMVPERQQALKELYGTPDRVEFYVGLIAADHPAGGK
IFSEAMTKFVANDAFNQALTNPLLSQNVWE

Synedropsis recta MMETSP1176-20130426_10672
PTKPWLGQGCRGMALRFPKRPLPDNLPDAKTLVEDFCIRPDDDASFTPCANGVNSL
TPYFALCVIHDFFRSDTGRSKVGKLDRPWVNLHSSYLDLQTVYGYNKKSCASVRTQKHGK
LTEKKIVDHRLERMAVCHALILVMVKHHNFICDQLMERYPDKFKTDEMIFQTARLINCGV
YINLIIELYSCLFHVWNEDGSNPVELRGLDYPRDIQGYHLSYEFNIMYRFHAFIPKEWTPFY
GLKEAIAYLKKGDFTHSVPALSDDAIKTLLVDAMSHRAGANHVPSNVPRVMGPAEVKGIE
DARLLGISTYNDFKEAVGEPKYDTFLDMSGNRPELAAKLEKHYPTVDDVEFAVGMRVETR
TPLRGAGFSTVGRAILADAFSSIRFDRFYSQSEYH

Nostoc WP015113127

MLFPYWVQWFTDSFLRLDHTNKLKNTSNHEIDLCNVYGLTRKQTHLLRSFQGGKF
KTQKLKRQDGVEEEYPLFYYADPAQGIVDPQFAGLYEPVNDEKRQPADKKQYLFAMGVE
RANVQIGYVMLNTLCLREHNRLCDVLASNYPDWDDERLFQTSRNILMAIILKIIMEEYINHI
TPYHFKLFADPEAFTKESWYRTNHMAIEFDFVYRWHSAIPETFNCNGKPTHVADTLWNNK
ILIDQGLGALMEETCSQAGTRIGLFNTPNILVDLAELPSIKLGRQLQLASYNDYRELCGFPRV
TSFDQITGDEFTQQKLKEFYGHVDNIEFFVGLYSEDVRQNSTIPPVARLIGIDAFSQALTNPL
LSPKIFN

Chaetoceros cf neogracile MMETSP1336-20130426_1470

LMLPIFAQWFTDSFLRTKFRVDGPQDFKENESNHEIDLCQIYGMTETQTAMLRSME
NGFLKSQIIDKEEFPAHLFEETASGLVQIKPEFAGLYTEANVQRVFRRASKEHKLNCFAVGI
EHGNSTMGNTLMNIIWMREHNRIAREIAKAHPTWDDERLFQTARNVNIVLLLKVVVEDYI
FHISGFPFKMDNKIAEGERWYRSNWMAAEFALLYRWHDLIPDTVEFGGERKDSSALNKNN
RWLMKVGVDRACLDASQEPSGKIMLGNTPNFLVEVGKMSLKQGRVSGLASFNDYCEHYG
LKRKKNFMDLTGNNKEVSKKLEDVYGSIDNLEWFVGLWAEAYDDKTKCMGPLTYMVGN
DAFTQALTNPLLAAEVFN

Grammatophora oceanica MMETSP0009_2-20130614_5553

LLLPMFAQWFTDGFLRTKWKPPTEQDYVENESTHQIDLNQIYGATEVQTNILRAKK
GGKLRSQMIKGEEYPEYLFDTETLQIKPEFEGLYSDVNFTRVFQNVSDEQKKVTFAVGLEH
GNSTVANTIMNTLFLREHNRLAGIIEAAHKDWDDERIFQTTRNCLIVILMKIVINDYVAHIA
DAPVFLDAGGFAEDQPWYRENWMSVEFNLLYRWHDMIPDQVSFTSADQVSFSSEDEYWD
MESSKTLVNNNTLMLELGVEQILLDASKQRAGQLGLFNTPDFLLHVHKETMKQCRMINLA
PYVDYCKEYGLTVPKTFRELTGGNLEYAKALESVYDSVEHVEWFVGLWAGGKALGQFSS
DLLILMVGHDAVTQLFTNPLLSRRVYN

Gersemia fruticosa AAF93168

WLFMFFAQHFTHEFFKTIYHSPAFTWGNHGVDVSHIYGQDMERQNKLRSFEDGKL
KSQTINGEEWPPYLKDVDNVTMQYPPNTPEDQKFALGHPFYSMLPGLFMYASIWLREHNR
VCTILRKEHPHWVDERLYQTGKLIITGELIKIVIEDYVNHLANYNLKLTYNPELVFDHGYDY
DNRIHVEFNHMYHWHPFSPDEYNISGSTYSIQDFMYHPEIVVKHGMSSFVDSMSKGLCGQ
MSHHNHGAYTLDVAVEVIKHQRELRMQSFNNYRKHFALEPYKSFEELTGDPKMSAELQE
VYGDVNAVDLYVGFFLEKGLTTSPFGITMIAFGAPYSLRGLLSNPVSSPTYWK

Gersemia fruticosa AAS48061

WLFMFFAQHFTHEFFKTIYHSPAFTWGNHGVDVSHIYGQDIERQNKLRSFQDGKLK
SQMINGEEWPPYLKDVDNVTMQYPPNTSEDQKFALGHPFYGMLPGLFMYASIWLREHNR
VCTILRKEHPHWEDERLYHTGKLIITGELIKIVIEDYVNHLANYNLKITYDPELVFDHGYDY
DNRIHVEFNHMYHWHPFSPDEYNISGSTYSIHEFMYHPEIVVKHGMSSFVDSMSKGLCGQ
MSHHNHGAYTLDVAVEVIKHQRELRMQSFNNYREHFALEPYKSFEEMTGDPKMAAELQE
TYGDVNAVDLYVGFFLEKGLTTSPFGITMIAFGAPYSLRGLLSNPVSSPTYWK

Plexaura homomalla AAU87497

WLFMFFAQHFTHQFFKTVHHSPAFSWGNHGVDVSHIYGQGVERENKLRAFKDGKL
KSQMINGEEYPPYLKDVDDLKMQYLENTAEEQKFALGHPFFSMLPGLFMYATIWLREHNR
VCMILRKEHPHWEDERIYQTGKLIITGELIKIVIEDYVNHLANYNMKLRYDPQLVFSRNYD
YDNRIHLEFNHLYHWHPFSPDQFNISGTTYAIKDFMYHPEIVVKHGMSSFVNAMSSGLCGK

MSHHNHGQYTLDVAVEVIKYQRELRMQSFNXYRRHFGLHAYKSFEEMTGNPKMAAELK
EVYGDVNAVDFYVGFFLEKSLTTSPFGITMIASGAPYSLRGLLSNPVSSPTYWK

Plexaura homomalla AAF93169
WLFMFFAQHFTHQFFKTVHHSPAFSWGNHGVDVSHIYGQGVERENKLRAFKDGKL
KSQMINGEEYPPYLKDVDGLKMQYLENTAEEQKFALGHPFFSMLPGLFMFATLWLREHNR
VCMILRKEHPHWEDERIYQTAKLIITGETIKIVIEDYINHLANYNMKLRYDPQLVFSRNYDY
DNRIHLEFNHLYHWHPFSPDQFNISGTTYTINDFMYHPEIVVKHGMSSFVNAMSSGLCGKM
SHHNHGQYTLDVAVEVIKYQRKLRMQSFNNYRRHFGLPAYKSFEEMTGDPKLAAELKEV
YGDVNAVDFYVGFFLEKSLPTSPFGITMIASGAPYSLRGLLSNPVSSPTYWK

Gammarus sp ADB65786
LLFQYYAQHFTHQFFRTNYTKGPQFTKGNGGVDVSNIYGLTERQRRALRSNVDGK
LKFQIINGEHFPPYLKDVPGISMEYPPHLPITEDNKFALGHPFFALLPGLFVYSTIWMREHNR
VCEVLKEQHPHWDDERLYHTAKLIITGEVIKITIEDYVQHLSQYKVDLKFKPQVVHGTRFQ
FHNRINVEFDHLYHWHPLIPEGIKVEDSYYSLMDMAFSTKSVFTHGLDAFVKALVTNRAG
KLTSRNHSPVTVPVLKKMLENSRILRFQGVNQYRKKFNMRPFRDFLDLTGDEELARDMEE
MYGDINAVEYYVGLIAEKDSPSLTPLTMVNVGGPWSVKGLIANPICSPHWWK

Caprella sp. ADB65785
LLFQYYAQHFTHQFFRTNYTMCPQFTKGNGGVDVSNIYGLTEQHRRAIRMNSDGK
LKYQVINDEHYPPYLRDVQGIEMDYPPHIPITEDNKFALGHPFFALLPGLFVFSTIWMREHN
RVCDVLKNQHPDWDDERLYQTAKLIITGEVIKITIEDYVQHLSQYKVDLKFKPQVVHGTRF
QFDNRINAEFNHLYHWHPLIPDGIQVEDKYYSLMDMAFSTKSVFTHGLDKFIESMATSRAG
KLSHSNHPLVTLPVLKKMMENGRKLRYQGINEYRKRFALKPFKDFMDLTGDEALAKDLQ
ELYGHVDAVEFYVGLLTEKDSPSLTPLTMVNVGGPWSVKGLIANPICSPHWWK

Nitzschia sp. MMETSP0014_2-20120614_6887
PQLNFWMLSFVNWFHDDNFRTLPDTDGAFTWSDRGSLHMTHLYGHTEYRQAALR
TMAGDGKMKTSSRLGWDYYPPLLMDVQADFPDFDMWTSQRGSKHKSTSAGQTSEQADE
NMPYYFAIGDPRFNLHLGHILWTSVGLYLHNTACVILQREDPGLTDEDIFQRARVIVFHIIQ
KIRLQDFVMDSISSTRDHIRIPYDPKLLREEFAHHFAYSGGNQPNFLEFNHLYQAWHALIPN
GLVLNEDVDDGEKDILPIRKTLWAPKLMTTNFTIGEMATSFASTPLTLYSPHNFPVFLRGVT
EAALKDERAQRMAPYNSYRELIGLDPITSFEQFAVDDPQKMAELYNNDVDSVDFIAGILAD
SNPHLPGNFFGVQLVLVALFALQDLANNPLILDPVMS

Skeletonema marinoi MMETSP0319-20121206_1511
EADVALVANIMKRDTNSFAPFNQLASAWIQFMTHDWFQHDASSSQGLKMKNVVT
HWWDASQMYGSSQEEVDAVRAEGGKLHLDVNDEIDYNASLPITGFRENWWAGLHILHTI
FAREHNHIVDILAQSYPSMTEDELFGTARNIIAVLLAKIHTVEWTPTLLDNAVSDMALNIN
WHGLQTVTSMYFKGKNIPDEVKDIIDEMKVPSVFGSNYTTEQTLFNTPFYMTEEFVSVYR
MHTLLPDAMILEGGKTVSLQELAFTDARNLVSDPSKTTATLLQAFAHTPAQALSLKNYPKS
LFNLQIGNGKSINLAEIDISRDRARGIPRYNDARRQLLLTPYKSMDDLTSDKEELKLLKSVY
TDIEQVDFLVGCLVDKDRPDGFAFGIVPYYIFVVMASRRLLSDRFFQEGLTEENYS

Pseudo-nitzschia pungens MMETSP1061-20121228_74475
DNVQVIAQRLLAREGFKPAGNQLNIIAAAWIQAQVHSWIQHLDGAPTSIEATAEAV
GPVCPVKKMNFFETEERPDGEYNSFRTQWWDASFVYGQNREQVHLGRLYKDGKLKVNES

NPDTLSFVEEGKSKIDVVGDQSNSWVGVTVLQVLFIKEHNYCAEMIKKENPQLTDEEIYGH
CRNIISALVAKIHTIDWTVELLKTEQLRVAMEINWKGATKAVFGDKAPFHPLRLINKPKAD
NKGVPFCLTEEFAAVYRLHPLLPPGLVVEHGEGKEEFIGIEKLLTTKGRDKMREPGMAKKI
MFSAFHYSCGHLKSSNYPFIMRKFTPTDHKGVDLQPPEDRVVDMAAIDLHRDRERGIQKY
NEFRRQLKLRPFKTWEALTGEENKSDNELTDAKKLELIYGPAPEGIEKIDLLVGDLYERKIA
DGFALSETSFMIFLLMASRRIDSDPYLNEYYNEEYYT

Thalassiosira rotula TR754|c0_g1_i1|m.3431

MLFPTFAQHLIDSFIVTAIKENTSEGVEFDWRKTGSPHDIGLLPLYGKTIEQTTQLRL
KSELKGSKGKLKTQIIKGGEWSPYLFDAKGNKKPEFSSLPDPEGLEFALGMAKQHGVDVK
KNKATVFALGGSRANLTPNIVAWNTLLLREHNRIASKIEEENPKWDDERVFQTARNVNLA
VYLRLVIEEYINHITGFGVDFTVEPGKWMWDAPWYKRNWISAEFAVLYRWHAVIPNCMK
WGEKTLPTASYLYSNHLLLDEDGMKGDLREAFINISDHRATSMQIHNTESWMKGRDARAL
QMSRACELRSFTEYCAYLGAKVPKTFADITSDVEVQNELKALYGKVENIEFWVGLIAKDH
PTESIMSAELTSFVANDAFNQALTHPLLSEHVWAAGPE

Skeletonema marinoi MMETSP0920-20130426_33289

LLFPTFAQHLIDSFINTKINVETGEFEWDRTDSKHEIGIGPLYGDEVEQTSQLREKSEK
PGRRGRLKTQVLEGDEEWAPFLYNEDGTKKEEFSAIHDPDGMKTILGLVYSSDPTTKSSIEQ
SIFAFGGRRANLNPNIVAWNTLLLREHNRLAGEIEKSEPGWDDERVFQTARNVLIVMYCKI
VIEEYIKHISGVNFKVDPGPWMWNAPWYKTNWMSTEFAILYRWHALIPNEAGLGPSKDAG
VMESLFNNPVLLDDETGLGGNLRDIFVDISQTRITSFQLFNTEKWMVERESAAINQGRANN
VQSYAAYCEYLDIEPPKTFEDISMVPERQQALKELYGTPDRVEFYVGLIAADHPAGGKIFSE
AMTKFVANDAFNQALTNPLLSQNVWE

Fragilariopsis kerguelensis MMETSP0907-20130614_13332

PNVQVVAQRLLARESFTPAGDQLNIVAAGWIQAMVHDWMKHEDGKKTSMEVTPA
VVGSQCPLHRFNLFETKERPDGHYNSERTNWWDASFVYGQNAEQVQNSRAFVGGKLKVN
EKNPDTLPSRDDGTDLTGDQSNSWVGVSVLQTLFLYEHNYCAEQIAKENPNLTDHQIYGH
CRNIIAALVAKIHTIDWTVELLKTPQLKIGMRTNWMGIIQAITGLKIPFLDRLLRLIKKKENN
NEGVPFCLTEEFAAVYRLHPLLPPGLIIEGEGAGDKDEDEDTFIDLRDTLTTKGRDLMRKSG
MAKKVMKAVFTYPCGNMAPSNYPDVMRDFHPTDLLGNNLDDRIDLAAIDLFRDRERGIQ
YFNNFRRKLSMKPFQTWEELTGDDKMTEEALAAFVAGTGNLTNAKKLELVYGAAPKGIE
HCDLLVGDLYEKKIPGFAISETSFMIFLLMASRRLDADPYLNEYFDEEHYT

Skeletonema grethea MMETSP0578-20130828_1168

ILVAYSIXSFQYVLDFKPAASQLNVLAASWIQAMVHDWIGHFDGDETETLDRGGES
LCPFAKSPFSFKNTKTEKIDGVPFSPSERTNWWDASFVYGNNSEQIDRARTMQGGKMVTS
DIPHALAEDKDGVYFAGDNKNSWVGVALLQDLFIREHNYICDQIAAEAKEEGKEMTDEEL
FGKARVVVAALVAKIHTVDWTVELLKTKLLAIGMKTNWDGLLKAVGIPIPGILSQMGEKK
GRVSDNEGTPFCLTEEFAAVYRLHSLSPPGLILGDGDAKDKFIGLEDLLGDEGRKQMRETK
TRPKEMMKSCLHWPCGALMSSNYPNAFRDVAPTDDYGKDLKSQNIDLAALDLFRDRERGI
LKFNEFRRQLNLKPYRTWLELTENEEDARKLELIYGPGQEGIERCDLLVGDMYERKVQPSF
ALSETSFIIFLLMASRRLSADPFLNELYNEETYS

Staurosira sp MMETSP1361-20130828_23824

LLFPFFAQWFVDSFLRTKWKPLAEQDFKENESNHDIDLNQIYGTSEIQTDMLRSMK
GGRLKSQIIDGEEYPVFLFDQKTATLKPEFRGLYTEENFKRVFGNASKEHKLHSFAVGLEH

GNSTIGNTVMNTLFLREHNRVAGVISAAHPEWDDERVFQTTRNVMIVLLIKIVLADYIYHIS
GAAVFADPGGFAEDELWYRENWMSVEFSLLYRWHDLIPSSVTFDGETRDAVDLQNNNRW
LLKAGMDSVIQDASNQKAGVMGLGNTPDFLLSVTKMSLHMARTCKLSSYVEYCKEYGQD
PPEDFMDLTGDQDSASKLERVYGSIDKVEWFVGLFAQKRDVFSGLLMTLMVGNDAVTQA
FTNPLLAKRVYN

Skeletonema marinoi MMETSP1428-20130617_31509

EADVALVANMMKRDTNSFAPFNQLASAWIQFMTHDWFQHDASSSQGLKMQNVV
THWWDASQMYGSSQEEVDAVRAEGGKLHLDVNDEIDYNASLPITGFRENWWAGLHILHT
VFAREHNHIVDILAQSYPSMTEDELFGTARNIIAALLAKIHTVEWTPKNIPDEVKDIIDEMKV
PSVFGSNYTTEQTLFNTPFYMTEEFVSVYRMHHLLPDEMILEGGKLQELAFTDARNLVSDP
SKTTATLLQAFAHTPAQALSLKNYPKSLFNLQIGNGKLINLAEIDISRDRARGIPRYNDARR
QLLLTPYKSMDDLTSDKEELKLLKSVYADIEQVDFLVGCLVDKDRPDGFAFGIYYIFVVMA
SRRLLSDRFFQ

Homo sapiens NP000953

LMFAFFAQHFTHQFFKTSGKMGPGFTKALGHGVDLGHIYGDNLERQYQLRLFKDG
KLKYQVLDGEMYPPSVEEAPVLMHYPRGIPPQSQMAVGQEVFGLLPGLMLYATLWLREH
NRVCDLLKAEHPTWGDEQLFQTTRLILIGETIKIVIEEYVQQLSGYFLQLKFDPELLFGVQFQ
YRNRIAMEFNHLYHWHPLMPDSFKVGSQEYSYEQFLFNTSMLVDYGVEALVDAFSRQIAG
RIGGGRNMDHHILHVAVDVIRESREMRLQPFNEYRKRFGMKPYTSFQELVGEKEMAAELE
ELYGDIDALEFYPGLLLEKCHPNSIFGESMIEIGAPFSLKGLLGNPICSPEYWK

Ovis aries NP001009476

LMFAFFAQHFTHQFFKTSGKMGPGFTKALGHGVDLGHIYGDNLERQYQLRLFKDG
KLKYQMLNGEVYPPSVEEAPVLMHYPRGIPPQSQMAVGQEVFGLLPGLMLYATIWLREHN
RVCDLLKAEHPTWGDEQLFQTARLILIGETIKIVIEEYVQQLSGYFLQLKFDPELLFGAQFQ
YRNRIAMEFNQLYHWHPLMPDSFRVGPQDYSYEQFLFNTSMLVDYGVEALVDAFSRQPA
GRIGGGRNIDHHILHVAVDVIKESRVLRLQPFNEYRKRFGMKPYTSFQELTGEKEMAAELE
ELYGDIDALEFYPGLLLEKCHPNSIFGESMIEMGAPFSLKGLLGNPICSPEYWK

Homo sapiens NP000954

MMFAFFAQHFTHQFFKTDHKRGPAFTNGLGHGVDLNHIYGETLARQRKLRLFKDG
KMKYQIIDGEMYPPTVKDTQAEMIYPPQVPEHLRFAVGQEVFGLVPGLMMYATIWLREHN
RVCDVLKQEHPEWGDEQLFQTSRLILIGETIKIVIEDYVQHLSGYHFKLKFDPELLFNKQFQ
YQNRIAAEFNTLYHWHPLLPDTFQIHDQKYNYQQFIYNNSILLEHGITQFVESFTRQIAGRV
AGGRNVPPAVQKVSQASIDQSRQMKYQSFNEYRKRFMLKPYESFEELTGEKEMSAELEAL
YGDIDAVELYPALLVEKPRPDAIFGETMVEVGAPFSLKGLMGNVICSPAYWK

Coccotylus truncatus AFN20596

VLIAYYAQWVTHQFFNTDESDPTGHSVKQPVGVNMSMLYGSKQEVEKSVRAYKG
GLLKSTIKNGQEFPEIMPCQEGSRIPGKEMFNMPILIANMIPGFAAIHVLFFRRHQYICRELA
KWAEAQGKNIDDEELFQKAKLIVTVNMLRITMHDYVSRALQSSHAKMRFDQKVKQSRIW
KMFGPDYFPPSNAIQFEFNIFYRWHQFYPDTTKIMKRIDDLKFPKSKQQLDEKWNAVRWIA
DEPDGMERVLFSASSQRAGKLSLLNTNQWIVEHVVKPGLARCREHQLASYNDYREKVGFP
RLTTFEQVTSNPALLEKLKRVYRNVDQIEYYPGVFAEDKHFGNVHGPLTFGSSMTFTGIFSS
RLFETALDE

Ciona intestinalis XP002127674

VLFPFFAQHFTHMFFKTDPMKGMPYQWGDQLVDLSQIYGHGEKRQHELRSHVNG
KLKVSLVDGHEFPPLSNQTTANMSNINLLPQEYQFVFGHQGFSLMPTFLIWSTIWLREHNRI
CDLIKEENPAWDDERIFQTARLVLTGETIKVVIEDYVQHLSGFHYKLLYDPELVQGGSHSF
HNQIHVEFQLLYHWHALMPDQIEFNGKSYTMKRLLFNPEPVVKGGLKRTIEDLSNQWAGQ
VAGGKTQGAATLHVAGLAIKNGRDLRMQSFNAYKEKFEMKKYTTFQELTGEEEMAAELQ
KLYGDIDAVEYYIGIMLEKRRSPQLFGETLTEMGSPYSLKGLYSNPINHKDWWK

Ciona intestinalis XP002123273

VFFAFFAQHFTHQFFKTNTIKGMPFQWGEHSVDLSHVYGHTIQRQHELRSHIDGKL
KVFETNGEVFPPLTESANVTMSGEKLMRGRKFAIGHPGFGAFPSFFVIATLWLREHNRVCDI
LKDLHPDWDDERLFQTARLILTGETLKIIVEDYVQHVSGFHFQLSYDPEILHKSTFSYNNQI
HAEFHILYHWHMLMPDFIELGEHVYPLKELLFNVDPVVEIGMETVLKQLSNQFAGKVVGG
RNQGPELVAVVELALKQTRQMRMCSFNKYRERFGMKPYTSFEELTGETEVAALLRNLYY
DIDALELFVGYFVEHRRNRQVLGATMLEMGAPYSLKGVFGNPIGSPAWWK

Oncorhynchus mykiss CAC10360.1

LMFAFFAQHFTHQFFKTRNSMGLGFTRALGHGVDAGNVYGDNLVRQLNLRLLKD
GKMKYQVVKGEVYPPTVAEAAVNMRYPQETPVGQRMAIGQEVFGLLPGLTMYATLWLR
EHNRVCDILKAEHPTWGDEQLFQTARLIVIGETIRIVIEEYVQHLSGYLLDLKFDPVLLFKST
FQYRNRIAVEFKQLYHWHPLMPDSFHIDGDEVPYSQFIFNTSIVTHYGVEKLVDAFSRQCA
GQIGGGRNIHPVVTNVAEGVIEESRTLRLQPFNEYRKRFNLKPYTSFSDFTGEEEMARELEE
LYGDIDALEFYPAIMLEKTRPNAIFGESMVEMGAPFSLKGLLGNPICSPEYWK

Fistulifera solaris GAX23950.1

LPPVKDVAAILRRPMNPTSVAPFNQIAVAWIQMMTHDWFQHDPAHPDQKMNRVT
HWWDASQLYGSTLAQQTAVRVPNTGKVRLDQHQELNYTTTGIPITGFADNWWAGLHMM
HTLFVREHNWLVDQFERQYPGVYTANDKFQLARLCLSALLAKIHTVEWTPTLLDNPVAAL
GLHTNWRGVDAILEYGTRFELQLAYRIVGGDQSVPHAGNGTTRETLYNTTFAMTEEFVAV
YRMHPLLPDEMEIEGKTFSLNDLSFVDARTLTKSVKTTQTLLQAFGMTPANTLSLQNYPRQ
LYGLEKPGMSQPVNLAEIDLQRDRERNLPRYNDMRRQLLLKPYKRLEDLTDDETELNLLK
SVYQDMDQVDLMVGCLVDKDRPYGFAFGIVPFHVFLVMASRRILNDRFFMEDFNAKVYT

Cyanothece sp Heam peroxidase ACB53655.1

PNPRVVSRTLMTREDFKPATILNLLAAAWIQFENHDWFSHGDNKPEDKLEIPLEAN
DPWPEEYRPLEVGKTLPDTSRPEGAKPPTFINTVTHWWDGSQIYGSNPETVDQLRSHEDGK
LIIGENGLLPVDPETGVDITGFNDNWWIGLGMLHTLFTREHNLICDHLKQEYSQWSDDDLF
DHARLINAALMAKIHTVDWTPAILPLPATDIALNVNWNGFLGEDIKQVLGTVGEGEIADLL
TGIIGSDKNHHTAPYYLTEEFVSVYRMHPLIPDELEFRSLEGDKFLQEVNFFEMSGKRTRAL
LESISLPDLFYSFGITHPGAITLHNYPRFLQQLVRDNGEVFDLAAVDILRDRERGVPRYNRFR
EIMGRGRVKCFEEISSNKQWVEEMRRVYNDNIDQVDLMVGLFAEDTPEGFGFSDTAFRVFI
LMASRRLKSDRFFTTDYRAEIYT

Fistulifera solaris GAX18717.1

LPPVKDVAAILRRPENPSTVAPFNQIAVAWIQMMTHDWFQHDPTNPDSKMNRVTH
WWDASQLYGSTLTQQRAVRVPNTGKLRLDKHQELNYTSTGIPITGFADNWWAGLHMMH
TLFVREHNWLADQFELQYPGVYTANDKFQLTRLCLSALLAKIHTIEWTPTLLDNPVAALGL
HTNWRGVDAILEYGTQFELQLAYRIVGGDQSVPHAGNGTTRETMYNTTFAMTEEFVAVY

RMHPLLPDDMEIEGITLTLNDLSFVDARKLTKSVKTTQTLLQAFGTTPANTLSLQNYPRQL
YGLEKPGMSQPINLAEIDLQRDRERNLPRYNDMRRQLLLKPYKRLEDLTDDETELNLLKSV
YQDIDQVDLMVGCLVDKDRPYGFAFGIVPFHVFLVMASRRILNDRFFMEDFNAKVYT

Alcanivorax nanhaiticus WP_035233415.1

PNPREVSNLIMSRGGDFKPATTLNFIATSWIQFMVHDWFDHGPRTDANPIEFPLPAG
DVLGGGTMSVQRTRPDPDVSGDEGIITYENINTHWWDGSQLYGSSKEKNDEVRSFVDGKL
KVDGDGRLPTEFFSGKPVTGFNENWWVGLSMLHHIFTQEHNAIADMLVANYPGQSDQWY
FDKARLINSALMAKIHTVEWTPAILANPVLERAMYANWWGLGGDRDKRDKYQDDLDNL
NNNLGQIGGLLDLVGIDNGLGDSPTGSLEHALAGLVGSRTPNNYNVPYTLTEEFVSVYRM
HPLLRDEIKVYDIGSNVVDEEIALEDTRNGDAEDLLGDIGQDRLWYSFGITHPGALTLNNYP
DFLRNLSMPLIGDIDMAAIDILRDRERGVPRYNEFRRQIGLKPLTSFEQLTSDPQLLADLKAL
YNNDIELVDTLVGQLGEETRPEGFGFGETSFQIFILNASRRLMTDRFFTTDYTDEVYT

Hordeum vulgare subsp. vulgare BAJ90503.1

PDPFVVATKLLARREYKDTGKQFNILAAAWIQFMVHDWMDHMEDTKQIEITAPKE
VANECPLKSFKFYATKEQPTNSDGIKTGYHNIRTAWWDGSAVYGNNEKQEKKIRTYADG
KLVIGDDGLLLHEENGVPLSGDVRNGWVGISILQALFVKEHNAVCDAIKEEHPNLSDEELY
RYAKLVTSAVIAKIHTIDWTVELLKTKTLRAGMRANWYGLLGKKIKDTFGHIGGTALGGL
VGLKKPINHGVPYSLTEEFTSVYRMHPLIPSTLKLRDPTGQPAADNSPPYLEDIDIGELVGLK
GEDQLSKIGFEKQTLSMGYQACGALELWNYPSFFRDLIPQNLDGTNRSDRIDLAALEVYRD
RERSVPRYNEFRRRLFLIPIKCWEDLTSDNDAIEAIRAIYGDDVEKLDLLVGLLAEKKIKGFA
ISETAFNIFILMASRRLEADRFLTSNFNEKTYT

Triticum aestivum CDM84254.1

PDPFVVATKLLARREYKDTGKQFNILAAAWIQFMVHDWMDHMEDTKQIEITAPKE
VANECPLKSFKFYATKEQPTNSDGIKTGYNNVRTAWWDGSAVYGNNEKQAEKTRTYVDG
KLVIGDDGLLLHEENGVPLSGDVRNGWVGVSILQALFVKEHNAVCDAIKEDHPNLSDEEL
YRYAKLVTSAVIAKIHTIDWTVELLKTKTMRAAMRANWYGLLGKKFKDTFGHIGGTALG
GLVGLKKPINHGVPYSLTEEFTSVYRMHSLIPTTLKLRDPTGQPAANNSPPYLEDIDIGELVG
LKGEDQLSKIGFEKQTLSMGYQACGALELWNYPSFFRDLIPQNLDGTNRSDRIDLAALEVY
RDRERSVPRYNEFRRRLFLIPIKSWEDLTSDKDAIESIRAIYGDDVEKLDLLVGLMAEKKIK
GFAISETAFNIFILMASRRLEADRFITSNFNEKTYT

Rhizophagus clarus GBC07128.1

LELEHYITALTQLPLNTPEEKKLLSILERLLVTQLWSDISKPPAMIAGDIYRSSDGSGY
NRLIQSLGKANSRYSLTSRIQYPIKLSVLPKSEDIFDKIMVQGGDFVEHPSGISSMLFYLAIIIT
HDLFHTSFADPNINLTSSYLDLTPLYGSNDQEQKSIRTLKGGLLKPDTFADSRILLQPPGVSA
LVILFSRNHNFIAQTLLEKNELGRFSVTNPNDPEQLKKQDEDLFQTARLINCGFYINVILHNY
LRTILGLDRTNSKWFVDPTVPYNKRGQLEPLPSGIGNIVSLEFNYIYRWHAATSKDDSKFVE
DEFKTIFGDDWENITIDEFKEKMGVWGRSIPKDPSKWKFNHIERGSDNRFKDTDIAKEIING
TKKVSGAFGANRIPKVFRPIELLGIESARVLGLSSLNDFRRSLNLKPYESFMEMNPDPLIAKK
LEELYGSIENVELYPGLMTEKTKPDMLGSAIALPFTISRAILSDAVNLVRNDRYYTNDFSPR
NLT

Emiliania huxleyi XP_005780718.1

PFQALHNLPSADEVVRLLYKRDAFKKAPYGVNSLATWFANVAIHDFFRTATGTDG
GTHPERGSDKEWVNLHSSYLDLQPLYGYSKTTADATREWSGGKLKAFAEDRMRRIPESRV

IVELLRREHNYVAEQLAQRYPAQFATDEELYQQARLIMGGVYINIILRAYGCQMFGEIAPD
GSGFCELRQGYGGAGVGNMCTFNFNLIYRFHTSIPVEWSATDPPPIDTDEQMRTLLNGILN
WESGGFGPNNVPDSILGERARVSQRAIEAARLMGAPTLNTFRRRFTSGYSSFEDMTGGDQA
TADTLRQLYPGGIEDVELVVGCQVEKCMSGGWALPSTIGQAIVADAFASIRQDRFYTQDW
GASSYT

Rhodococcus gordoniae WP_064063209.1

VWFAFFAQWFTDSFLRTNSKDPRKNDSNHEIDLCQIYGVNQAKTTMLRAGYGGRL
DSQVIDKKEYPPFLFAARTPGEELRFVPKFEGLHDREYLLDTVLRLCPDERKKSVFAVGLE
HGNSTIGNTVLNVLFLREHNRIAGILEGAYPEWDDDRLFETTRNIMIVILLKIVIEEYIKHIGP
FDFPIEFVPFMADNAPWNRTNWCAIEFNLLYRWHSLIPDTVVFDSQRVSTRILVDNNPLVL
DRGIESIIDQCSRQKASRIGLGNTPAFLIDRHPMCPDRESVEERTVGLMRQARLRSFNDYRE
AFGLGRLTSFTELTGDVEVQQKLARLYGDVDAVEWYVGIFAEDYPRHRMMGELLTTMVA
HDAFTQAFTNPLLARHVYHEDTF

Herbidospora mongoliensis WP_066371138.1

VMFMFFAQWFTDSFLRTSRDDFRRNTSTQEIDLCQIYGLTEEKTHLLRAHRDGRLK
SQLIDGEEYPEFLFRPRSSGEPPVFKPEFEGLHDSEFIVSRLLEDAPEKQKDTFFAVGLEHGN
STIGNTIMNVVFLREHNRIAGLLKQAHPEWAERPEGADARLFETTRNIMIVLLLKLVVEEYI
RHISPYDFPLETVPFIADGKRWNRSNWISIEFNLLYRWHSLVPSTIGSGPDRLDSTDFRNNNP
LVLARGIESLVSQCSGERAGRIGLMNTPGFLVDRDPAHPERPSVQERTIALSRKARLASYND
YRENFGLGRLKDFGRLTQDAELRERLEKLYDDIDKLEWYVGIFAEDYSRDEMMGRLMTT
MVAYDAFTQALTNPLLARDVYNEATF

Eutrema halophilum BAJ34623.1

MVVATKLLTRRKMIDTGKQFNMIAASWIQFMIHDWVDHLEDTDQIELSAPKEAAK
GCPLSSFRFFKTKEVPTGFFEIKTGSLNTRTPWWDSSVIYGSNSKTLERVRTYKDGKLKISEE
TGLLLHDDDGLAISGDIRNSWVGVSALQALFIKEHNAVCDLLKKEYEDLEDEDLYRHARL
VTSAVIAKIHTIDWTVELLKTDTLLAGMRANWYGLLGKKFKDTFGHVGSSIFGGVVGMKK
PQNHGVPYSLTEEFTSVYRMHSLLPDQLHMRDIDVTPGPNKSLPLTQEVSMEKLIGREGEE
TMSQIGFTKLMVSMGHQACGALELMNYPAWFRDLVPQDPNGHDRPDHIDLAALEIYRDR
ERNVARYNDFRRAMFMIPIKTWEDLTDDKEAIELLDDVYGGDVDELDLLVGLMAEKKIKG
FAISETAFNIFLLMATRRLEADRFFTSDFNEMTYT

Pisum sativum CAH05011.1

PDPMVVVTKLLERKTYKDTGTQFNVIAASWIQFMIHDWIDHMEDTKQVELSAPSEV
ASQCPLKSFKFFKTKEIPTGFYDIKTGHANVRTPWWDGSVVYGSNEQVLNKVRTFKDGKL
KISKEGHLLHNEDGTAISGDIRNSWAGVTTLQTLFVQEHNAVCDALKKENSDLEDEDLYR
HARLVTSAVIAKIHTIDWTVELLKTDTLLAGMRANWYGLLGKQFKDRFGHVGNSILSGFV
GMKRSENHGVPYSLTEEFATVYRMHPLLPDSLHLRDISASPGPNKSPPLIKEIPMNDLIGLQ
GEKTLLEIGNAKKLVSMGHQACGALELWNYPSWLRNLVPHNIDGTERSDHVDLAALEVY
RDRERNVARYNQFRRGLLLIPISKWEDLTDDEEAIKVLEEVYGDDVEELDVLVGLMAEKKI
KGFAISETAFVIFLLMASRRLEADRFFTSNFNEETYT

References

1. Kelley, L. A., Mezulis, S., Yates, C. M., Wass, M. N. & Sternberg, M. J. E. The Phyre2 web portal for protein modeling, prediction and analysis. *Nature Protocols* **10**, 845–858 (2015).
